# Supplementary material for: AAV gene therapy rescues hearing and balance in a model of CLIC5 deafness
Source: EMBO Mol Med. 2025 Aug 26;17(9):2233–57. doi: 10.1038/s44321-025-00275-7 (PMC12423326; doi:10.1038/s44321-025-00275-7)
Supplement: Supplementary file 1 — Table EV1 [file 44321_2025_275_MOESM1_ESM.docx]

| **Gene** | **CDS Size (bp)** | **Deafness form** | **Protein name** |
| --- | --- | --- | --- |
| TMIE | 471 | DFNB6 | Transmembrane inner ear expressed protein |
| CIB2 | 564 | DFNB48 | Calcium integrin binding protein 2 |
| MINAR2 | 573 | DFNB48 | Membrane integral notch-2 |
| MSRB3 | 579 | DFNB74 | Methionine sulfoxide reductase B3 |
| MPZL2 | 648 | DFNB111 | Myelin protein zero like-2 |
| CLDN9 | 654 | DFNB116 | Claudin-9 |
| GRAP | 654 | DFNB114 | GRB2-related adaptor protein |
| LHFPL5 | 660 | DFNB66/67 | LHFP-like protein 5 |
| CABP2 | 663 | DFNB93 | Calcium binding protein 2 |
| GJB2 | 681 | DFNB1 | Gap junction protein B2 (Connexin 26) |
| CLDN14 | 720 | DFNB29 | Claudin 14 |
| GRXCR2 | 747 | DFNB101 | Glutaredoxin domain-containing cysteine-rich protein 2 |
| GJB6 | 786 | DFNB1B | Gap junction beta 6 |
| WBP2 | 786 | DFNB107 | WW domain binding protein 2 |
| GRXCR1 | 873 | DFNB25 | Glutaredoxin domain-containing cysteine-rich protein 1 |
| LRTOMT | 876 | DFNB63 | Leucine-rich transmembrane O-methyltransferase |
| PRPS1 | 957 | DFNX1 | Ribose-phosphate pyrophosphokinase 1 |
| BSND | 963 | DFNB73 | Barttin |
| PJVK | 1059 | DFNB59 | Pejvakin |
| S1PR2 | 1062 | DFNB68 | Sphingosine-1-phosphate receptor |
| POU3F4 | 1086 | DFNX2 | POU domain, class 3, transcription factor 4 |
| LMX1A | 1149 | DFNA7 | LIM homeobox transcription factor 1-alpha |
| ELMOD3 | 1176 | DFNB88 | ELMO domain containing 3 |
| SERPINB6 | 1188 | DFNB91 | Serpin B6 |
| SYNE4 | 1215 | DFNB76 | Spectrin repeat-containing nuclear envelope |
| CEACAM16 | 1278 | DFNB113 | Carcinoembryonic antigen-related cell adhesion molecule 16 |
| TMPRSS3 | 1365 | DFNB8 | Transmembrane protease serine 3 |
| DCDC2 | 1431 | DFNB66 | Doublecortin domain-containing protein 2 |
| NARS2 | 1434 | DFNB94 | Asparaginyl-tRNA synthetase, mitochondrial |
| ESRRB | 1527 | DFNB35 | Estrogen related receptor beta |
| ILDR1 | 1641 | DFNB42 | Immunoglobulin like domain containing receptor 1 |
| SPNS2 | 1650 | DFNB115 | Sphingolipid transporter 2 |
| SLC22A4 | 1656 | DFNB60 | Solute carrier family 22 member 4 |
| MARVELD2 | 1677 | DFNB49 | MARVEL domain-containing protein 2 |
| TBC1D24 | 1680 | DFNB86 | TBC1 domain family member 4 |
| RDX | 1815 | DFNB24 | Radixin |
| AIFM1 | 1842 | DFNX5 | Apoptosis-inducing factor 1, mitochondrial |
| COCH | 1848 | DFNB110 | Cochlin |
| CDC14A | 1872 | DFNB32/105 | Cell division cycle 14 |
| KARS1 | 1878 | DFNB89 | Lysine-tRNA ligase |

**Table EV1. Human genes associated with hearing loss and vestibular dysfunction suitable for delivery via self-complementary AAV**
