# Supplementary material for: AAV gene therapy rescues hearing and balance in a model of CLIC5 deafness
Source: EMBO Mol Med. 2025 Aug 26;17(9):2233–57. doi: 10.1038/s44321-025-00275-7 (PMC12423326; doi:10.1038/s44321-025-00275-7)
Supplement: Supplementary file 2 — Appendix [file 44321_2025_275_MOESM2_ESM.pdf]

Appendix Table of Contents

Appendix Table S1: Exact p-values

Figures	Page
1A, 1B,1C	2
1E, 1G, 3B, 3C	3
3D, 4B, 4C	4
4D, 4E, 4G, 5B	5
5C, 5F, 5I, 5J, 5K, 5L	6
6E, 6F, 6G, 7D, 7E	7
7F, 7I, 7J	8
7K, 7L, 7N, 8B	9
8C, 8E, 8H, 8I, 8J, 8K	10
EV3B, EV3C, EV3D	11
EV4A, EV4B	12
EV4C, EV4D	13
EV5A ,EV5B, EV5C	14

1A (ABR 4w)
Adjusted P Value

Frequency (kHz)	Clic5 $^{+/+}$ vs. Clic5 $^{+/-}$	Clic5 $^{+/+}$ vs. Clic5 $^{-/-}$	Clic5 $^{+/-}$ vs. Clic5 $^{-/-}$
6	0.5336	<0.0001	<0.0001
12	0.1473	<0.0001	<0.0001
18	0.5336	<0.0001	<0.0001
24	0.1473	<0.0001	<0.0001
30	0.5336	<0.0001	<0.0001
35	0.0135	<0.0001	<0.0001

1B (ABR 8w)
Adjusted P Value

Frequency (kHz)	Clic5 $^{+/+}$ vs. Clic5 $^{+/-}$	Clic5 $^{+/+}$ vs. Clic5 $^{-/-}$	Clic5 $^{+/-}$ vs. Clic5 $^{-/-}$
6	0.6773	<0.0001	<0.0001
12	>0.9999	<0.0001	<0.0001
18	0.6773	<0.0001	<0.0001
24	0.5326	<0.0001	<0.0001
30	0.1463	<0.0001	<0.0001
35	0.001	<0.0001	<0.0001

1C (DPOAE 4w)
Adjusted P Value

Frequency (kHz)	Clic5 $^{+/+}$ vs. Clic5 $^{+/-}$	Clic5 $^{+/+}$ vs. Clic5 $^{-/-}$	Clic5 $^{+/-}$ vs. Clic5 $^{-/-}$
6	0.1745	0.1365	0.0052
12.4	0.1745	<0.0001	<0.0001
18	0.2271	<0.0001	<0.0001
24	0.8798	<0.0001	<0.0001
30.2	0.0243	<0.0001	<0.0001
35	0.7623	<0.0001	<0.0001

1E (Circling behavior)
Adjusted P Value

Clic5 $^{+/+}$ vs. Clic5 $^{+/-}$	Clic5 $^{+/+}$ vs. Clic5 $^{-/-}$	Clic5 $^{+/-}$ vs. Clic5 $^{-/-}$
0.7061	<0.0001	0.0006

1G (Rotarod 8w)
Adjusted P Value

Clic5 $^{+/+}$ vs. Clic5 $^{+/-}$	Clic5 $^{+/+}$ vs. Clic5 $^{-/-}$	Clic5 $^{+/-}$ vs. Clic5 $^{-/-}$
0.7813	0.0007	0.0037

3B (stereocilia length)
Adjusted P Value

Frequency (kHz)	Clic5 $^{+/-}$ vs. Clic5 $^{-/-}$	Clic5 $^{+/-}$ vs. Clic5 $^{-/-}$ +ssAAV	Clic5 $^{-/-}$ vs. Clic5 $^{-/-}$ +ssAAV
8	<0.0001	0.255	<0.0001
16	<0.0001	0.9243	<0.0001
32	<0.0001	0.9243	<0.0001

3C (OHC count)
Adjusted P Value

Frequency (kHz)	Clic5 $^{+/-}$ vs. Clic5 $^{-/-}$	Clic5 $^{+/-}$ vs. Clic5 $^{-/-}$ +ssAAV	Clic5 $^{-/-}$ vs. Clic5 $^{-/-}$ +ssAAV
8	0.0003	0.5085	0.0001
16	0.0006	0.194	<0.0001
32	<0.0001	0.0888	<0.0001

3D (IHC count)
Adjusted P Value

Frequency (kHz)	Clic5 $^{+/-}$ vs. Clic5 $^{-/-}$	Clic5 $^{+/-}$ vs. Clic5 $^{-/-}$ +ssAAV	Clic5 $^{-/-}$ vs. Clic5 $^{-/-}$ +ssAAV
8	0.1907	0.3979	0.0535
16	0.2719	>0.9999	0.2719
32	0.0055	0.3979	0.0012

4B (ABR 4w)
Adjusted P Value

Frequency (kHz)	Clic5 $^{+/-}$ vs. Clic5 $^{-/-}$	Clic5 $^{+/-}$ vs. Clic5 $^{-/-}$ +ssAAV	Clic5 $^{-/-}$ vs. Clic5 $^{-/-}$ +ssAAV
6	<0.0001	0.8146	<0.0001
12	<0.0001	0.2153	<0.0001
18	<0.0001	0.0289	<0.0001
24	<0.0001	<0.0001	<0.0001
30	<0.0001	<0.0001	<0.0001
35	<0.0001	<0.0001	0.0066

4C (ABR 8w)
Adjusted P Value

Frequency (kHz)	Clic5 $^{+/-}$ vs. Clic5 $^{-/-}$	Clic5 $^{+/-}$ vs. Clic5 $^{-/-}$ +ssAAV	Clic5 $^{-/-}$ vs. Clic5 $^{-/-}$ +ssAAV
6	<0.0001	0.2101	<0.0001
12	<0.0001	0.0119	<0.0001
18	<0.0001	<0.0001	<0.0001
24	<0.0001	<0.0001	0.0028
30	<0.0001	<0.0001	0.1355
35	<0.0001	<0.0001	0.6335

4D (ABR 12w)
Adjusted P Value

Frequency (kHz)	Clic5 $^{+/-}$ vs. Clic5 $^{-/-}$	Clic5 $^{+/-}$ vs. Clic5 $^{-/-}$ +ssAAV	Clic5 $^{-/-}$ vs. Clic5 $^{-/-}$ +ssAAV
6	<0.0001	0.1734	<0.0001
12	<0.0001	<0.0001	<0.0001
18	<0.0001	<0.0001	<0.0001
24	<0.0001	<0.0001	0.0311
30	<0.0001	<0.0001	0.7338
35	<0.0001	<0.0001	0.9447

4E (DPOAE 4w)
Adjusted P Value

Frequency (kHz)	Clic5 $^{+/-}$ vs. Clic5 $^{-/-}$	Clic5 $^{+/-}$ vs. Clic5 $^{-/-}$ +ssAAV	Clic5 $^{-/-}$ vs. Clic5 $^{-/-}$ +ssAAV
6	0.1166	0.7567	0.7567
12.4	<0.0001	0.0001	<0.0001
18	<0.0001	<0.0001	<0.0001
24	<0.0001	<0.0001	0.3579
30.2	<0.0001	<0.0001	0.6535
35	0.0002	0.004	0.9589

4G (Fear conditioning)
Adjusted P Value

Clic5 $^{+/-}$ vs. Clic5 $^{+/-}$ after	Clic5 $^{-/-}$ vs. Clic5 $^{-/-}$ after	Clic5 $^{-/-}$ + ssAAV before vs. Clic5 $^{-/-}$ +ssAAV after
<0.0001	0.385	0.0265

5B (stereocilia length)
Adjusted P Value

Clic5 $^{+/-}$ vs. Clic5 $^{-/-}$	Clic5 $^{+/-}$ vs. Clic5 $^{-/-}$ +ssAAV	Clic5 $^{-/-}$ vs. Clic5 $^{-/-}$ +ssAAV
<0.0001	0.053	<0.0001

5C (VHC count)
Adjusted P Value

Clic5 $^{+/-}$ vs. Clic5 $^{-/-}$	Clic5 $^{+/-}$ vs. Clic5 $^{-/-}$ +ssAAV	Clic5 $^{-/-}$ vs. Clic5 $^{-/-}$ +ssAAV
0.0126	0.9965	0.0138

5F (Rotarod)
Adjusted P Value

Clic5 $^{+/-}$ vs. Clic5 $^{-/-}$	Clic5 $^{+/-}$ vs. Clic5 $^{-/-}$ +ssAAV	Clic5 $^{-/-}$ vs. Clic5 $^{-/-}$ +ssAAV
0.0145	>0.9999	0.0181

5I (Traveled distance)
Adjusted P Value

Clic5 $^{+/-}$ vs. Clic5 $^{-/-}$	Clic5 $^{+/-}$ vs. Clic5 $^{-/-}$ +ssAAV	Clic5 $^{-/-}$ vs. Clic5 $^{-/-}$ +ssAAV
<0.0001	>0.9999	<0.0001

5J (Circling behavior)
Adjusted P Value

Clic5 $^{+/-}$ vs. Clic5 $^{-/-}$	Clic5 $^{+/-}$ vs. Clic5 $^{-/-}$ +ssAAV	Clic5 $^{-/-}$ vs. Clic5 $^{-/-}$ +ssAAV
<0.0001	0.9989	0.0002

5K (time in the center)
Adjusted P Value

Clic5 $^{+/-}$ vs. Clic5 $^{-/-}$	Clic5 $^{+/-}$ vs. Clic5 $^{-/-}$ +ssAAV	Clic5 $^{-/-}$ vs. Clic5 $^{-/-}$ +ssAAV
0.1872	0.8286	0.6457

5L (Duration to frequency)
Adjusted P Value

Clic5 $^{+/-}$ vs. Clic5 $^{-/-}$	Clic5 $^{+/-}$ vs. Clic5 $^{-/-}$ +ssAAV	Clic5 $^{-/-}$ vs. Clic5 $^{-/-}$ +ssAAV
0.003	0.9019	0.0045

6E (GFP positive HCs)
Adjusted P Value

	ssAAV.GFP vs. scAAV.GFP
IHC	0.0226
OHC	0.6454

6F (OHC intensity)
Adjusted P Value

ssAAV.GFP vs. scAAV.GFP
<0.0001

6G (IHC intensity)
Adjusted P Value

ssAAV.GFP vs. scAAV.GFP
<0.0001

7D (Stereocilia length)
Adjusted P Value

Frequency (kHz)	Clic5 $^{+/-}$ vs. Clic5 $^{-/-}$	Clic5 $^{+/-}$ vs. Clic5 $^{-/-}$ +scAAV	Clic5 $^{-/-}$ vs. Clic5 $^{-/-}$ +scAAV
8	<0.0001	0.8375	<0.0001
16	<0.0001	0.8451	<0.0001
32	<0.0001	0.9357	<0.0001

7E (OHC count)
Adjusted P Value

Frequency (kHz)	Clic5 $^{+/-}$ vs. Clic5 $^{-/-}$	Clic5 $^{+/-}$ vs. Clic5 $^{-/-}$ +scAAV	Clic5 $^{-/-}$ vs. Clic5 $^{-/-}$ +scAAV
8	0.0005	0.8236	0.0005
16	0.0005	0.1916	<0.0001
32	<0.0001	0.0872	<0.0001

7F (IHC count)
Adjusted P Value

Frequency (kHz)	Clic5 $^{+/-}$ vs. Clic5 $^{-/-}$	Clic5 $^{+/-}$ vs. Clic5 $^{-/-}$ +scAAV	Clic5 $^{-/-}$ vs. Clic5 $^{-/-}$ +scAAV
8	0.1907	0.3979	0.0535
16	0.2719	>0.9999	0.2719
32	0.0055	0.3979	0.0012

7I (ABR 4w)
Adjusted P Value

Frequency (kHz)	Clic5 $^{+/-}$ vs. Clic5 $^{-/-}$	Clic5 $^{+/-}$ vs. Clic5 $^{-/-}$ +scAAV	Clic5 $^{-/-}$ vs. Clic5 $^{-/-}$ +scAAV
6	<0.0001	0.6599	<0.0001
12	<0.0001	0.0084	<0.0001
18	<0.0001	<0.0001	<0.0001
24	<0.0001	<0.0001	<0.0001
30	<0.0001	<0.0001	0.0346
35	<0.0001	<0.0001	0.1773

7J (ABR 8w)
Adjusted P Value

Frequency (kHz)	Clic5 $^{+/-}$ vs. Clic5 $^{-/-}$	Clic5 $^{+/-}$ vs. Clic5 $^{-/-}$ +scAAV	Clic5 $^{-/-}$ vs. Clic5 $^{-/-}$ +scAAV
6	<0.0001	0.2101	<0.0001
12	<0.0001	0.163	<0.0001
18	<0.0001	<0.0001	<0.0001
24	<0.0001	<0.0001	0.0002
30	<0.0001	<0.0001	0.1355
35	<0.0001	<0.0001	0.2536

7K (ABR 12w)
Adjusted P Value

Frequency (kHz)	Clic5 $^{+/-}$ vs. Clic5 $^{-/-}$	Clic5 $^{+/-}$ vs. Clic5 $^{-/-}$ +scAAV	Clic5 $^{-/-}$ vs. Clic5 $^{-/-}$ +scAAV
6	<0.0001	0.0213	<0.0001
12	<0.0001	<0.0001	<0.0001
18	<0.0001	<0.0001	<0.0001
24	<0.0001	<0.0001	0.3372
30	<0.0001	<0.0001	0.8183
35	<0.0001	<0.0001	0.9447

7L (DPOAE 4w)
Adjusted P Value

Frequency (kHz)	Clic5 $^{+/-}$ vs. Clic5 $^{-/-}$	Clic5 $^{+/-}$ vs. Clic5 $^{-/-}$ +scAAV	Clic5 $^{-/-}$ vs. Clic5 $^{-/-}$ +scAAV
6	0.1166	0.7262	0.7567
12.4	<0.0001	<0.0001	<0.0001
18	<0.0001	<0.0001	<0.0001
24	<0.0001	<0.0001	0.7995
30.2	<0.0001	<0.0001	>0.9999
35	0.0002	0.0004	>0.9999

7N (Fear conditioning)
Adjusted P Value

Clic5 $^{+/-}$ vs. Clic5 $^{+/-}$ after	Clic5 $^{-/-}$ vs. Clic5 $^{-/-}$ after	Clic5 $^{-/-}$ + scAAV before vs. Clic5 $^{-/-}$ +scAAV after
<0.0001	0.4237	<0.0001

8B (stereocilia length)
Adjusted P Value

Clic5 $^{+/-}$ vs. Clic5 $^{-/-}$	Clic5 $^{+/-}$ vs. Clic5 $^{-/-}$ +scAAV	Clic5 $^{-/-}$ vs. Clic5 $^{-/-}$ +scAAV
<0.0001	0.6098	<0.0001

8C (VHC count)
Adjusted P Value

Clic5 $^{+/-}$ vs. Clic5 $^{-/-}$	Clic5 $^{+/-}$ vs. Clic5 $^{-/-}$ +scAAV	Clic5 $^{-/-}$ vs. Clic5 $^{-/-}$ +scAAV
0.0187	0.2514	0.0057

8E (Rotarod)
Adjusted P Value

Clic5 $^{+/-}$ vs. Clic5 $^{-/-}$	Clic5 $^{+/-}$ vs. Clic5 $^{-/-}$ +scAAV	Clic5 $^{-/-}$ vs. Clic5 $^{-/-}$ +scAAV
0.0145	0.9677	0.0047

8H (Traveled distance)
Adjusted P Value

Clic5 $^{+/-}$ vs. Clic5 $^{-/-}$	Clic5 $^{+/-}$ vs. Clic5 $^{-/-}$ +scAAV	Clic5 $^{-/-}$ vs. Clic5 $^{-/-}$ +scAAV
<0.0001	0.8067	<0.0001

8I (Circling behavior)
Adjusted P Value

Clic5 $^{+/-}$ vs. Clic5 $^{-/-}$	Clic5 $^{+/-}$ vs. Clic5 $^{-/-}$ +scAAV	Clic5 $^{-/-}$ vs. Clic5 $^{-/-}$ +scAAV
<0.0001	0.9997	<0.0001

8J (time in the center)
Adjusted P Value

Clic5 $^{+/-}$ vs. Clic5 $^{-/-}$	Clic5 $^{+/-}$ vs. Clic5 $^{-/-}$ +scAAV	Clic5 $^{-/-}$ vs. Clic5 $^{-/-}$ +scAAV
0.1977	0.8354	0.5811

8K (Duration to frequency)
Adjusted P Value

Clic5 $^{+/-}$ vs. Clic5 $^{-/-}$	Clic5 $^{+/-}$ vs. Clic5 $^{-/-}$ +scAAV	Clic5 $^{-/-}$ vs. Clic5 $^{-/-}$ +scAAV
0.0141	0.7047	0.0037

EV3B (Traveled distance)
Adjusted P Value

Clic5 $^{+/-}$ vs. Clic5 $^{-/-}$	Clic5 $^{+/-}$ vs. Clic5 $^{-/-}$ +ssAAV.GFP	Clic5 $^{+/-}$ vs. Clic5 $^{-/-}$ +scAAV.GFP	Clic5 $^{-/-}$ vs. Clic5 $^{-/-}$ +ssAAV.GFP	Clic5 $^{-/-}$ vs. Clic5 $^{-/-}$ +scAAV.GFP	Clic5 $^{-/-}$ +ssAAV.GFP vs. Clic5 $^{-/-}$ +scAAV.GFP
0.0009	0.0507	0.3104	>0.9999	0.8038	0.8822

EV3C (Circling behavior)
Adjusted P Value

Clic5 $^{+/-}$ vs. Clic5 $^{-/-}$	Clic5 $^{+/-}$ vs. Clic5 $^{-/-}$ +ssAAV.GFP	Clic5 $^{+/-}$ vs. Clic5 $^{-/-}$ +scAAV.GFP	Clic5 $^{-/-}$ vs. Clic5 $^{-/-}$ +ssAAV.GFP	Clic5 $^{-/-}$ vs. Clic5 $^{-/-}$ +scAAV.GFP	Clic5 $^{-/-}$ +ssAAV.GFP vs. Clic5 $^{-/-}$ +scAAV.GFP
<0.0001	0.0136	0.0503	0.9825	0.8029	0.9743

EV3D (Rotarod)
Adjusted P Value

Clic5 $^{+/-}$ vs. Clic5 $^{-/-}$	Clic5 $^{+/-}$ vs. Clic5 $^{-/-}$ +ssAAV.GFP	Clic5 $^{+/-}$ vs. Clic5 $^{-/-}$ +scAAV.GFP	Clic5 $^{-/-}$ vs. Clic5 $^{-/-}$ +ssAAV.GFP	Clic5 $^{-/-}$ vs. Clic5 $^{-/-}$ +scAAV.GFP	Clic5 $^{-/-}$ +ssAAV.GFP vs. Clic5 $^{-/-}$ +scAAV.GFP
0.0065	0.0676	0.0425	0.9993	0.9976	>0.9999

EV4A (ABR 4w)
Adjusted P Value

Frequency (kHz)	Clic5$^{+/-}$ vs. Clic5$^{-/-}$	Clic5$^{+/-}$ vs. Clic5$^{-/-}$ +ssAAV	Clic5$^{+/-}$ vs. Clic5$^{-/-}$ +scAAV	Clic5$^{-/-}$ vs. Clic5$^{-/-}$ +ssAAV	Clic5$^{-/-}$ vs. Clic5$^{-/-}$ +scAAV	Clic5$^{-/-}$ +ssAAV vs. Clic5$^{-/-}$ +scAAV
6	<0.0001	0.8146	0.6599	<0.0001	<0.0001	0.8146
12	<0.0001	0.2153	0.0084	<0.0001	<0.0001	0.2198
18	<0.0001	0.0289	<0.0001	<0.0001	<0.0001	0.0976
24	<0.0001	<0.0001	<0.0001	<0.0001	<0.0001	0.0352
30	<0.0001	<0.0001	<0.0001	<0.0001	0.0346	0.0213
35	<0.0001	<0.0001	<0.0001	0.0066	0.1773	0.1345

EV4B (ABR 8w)
Adjusted P Value

Frequency (kHz)	Clic5$^{+/-}$ vs. Clic5$^{-/-}$	Clic5$^{+/-}$ vs. Clic5$^{-/-}$ +ssAAV	Clic5$^{+/-}$ vs. Clic5$^{-/-}$ +scAAV	Clic5$^{-/-}$ vs. Clic5$^{-/-}$ +ssAAV	Clic5$^{-/-}$ vs. Clic5$^{-/-}$ +scAAV	Clic5$^{-/-}$ +ssAAV vs. Clic5$^{-/-}$ +scAAV
6	<0.0001	0.2101	0.2101	<0.0001	<0.0001	0.9469
12	<0.0001	0.0119	0.163	<0.0001	<0.0001	0.249
18	<0.0001	<0.0001	<0.0001	<0.0001	<0.0001	0.4775
24	<0.0001	<0.0001	<0.0001	0.0028	0.0002	0.5197
30	<0.0001	<0.0001	<0.0001	0.1355	0.1355	0.9823
35	<0.0001	<0.0001	<0.0001	0.6335	0.2536	0.6335

EV4C (ABR 12w)
Adjusted P Value

Frequency (kHz)	Clic5 $^{+/-}$ vs. Clic5 $^{-/-}$	Clic5 $^{+/-}$ vs. Clic5 $^{-/-}$ +ssAAV	Clic5 $^{+/-}$ vs. Clic5 $^{-/-}$ +scAAV	Clic5 $^{-/-}$ vs. Clic5 $^{-/-}$ +ssAAV	Clic5 $^{-/-}$ vs. Clic5 $^{-/-}$ +scAAV	Clic5 $^{-/-}$ +ssAAV vs. Clic5 $^{-/-}$ +scAAV
6	<0.0001	0.1734	0.0213	<0.0001	<0.0001	0.4058
12	<0.0001	<0.0001	<0.0001	<0.0001	<0.0001	0.7504
18	<0.0001	<0.0001	<0.0001	<0.0001	<0.0001	0.9805
24	<0.0001	<0.0001	<0.0001	0.0311	0.3372	0.3372
30	<0.0001	<0.0001	<0.0001	0.7338	0.8183	0.8183
35	<0.0001	<0.0001	<0.0001	0.9447	0.9447	0.9447

EV4D (DPOAE 4w)
Adjusted P Value

Frequency (kHz)	Clic5 $^{+/-}$ vs. Clic5 $^{-/-}$	Clic5 $^{+/-}$ vs. Clic5 $^{-/-}$ +ssAAV	Clic5 $^{+/-}$ vs. Clic5 $^{-/-}$ +scAAV	Clic5 $^{-/-}$ vs. Clic5 $^{-/-}$ +ssAAV	Clic5 $^{-/-}$ vs. Clic5 $^{-/-}$ +scAAV	Clic5 $^{-/-}$ +ssAAV vs. Clic5 $^{-/-}$ +scAAV
6	0.1166	0.7567	0.7262	0.7567	0.7567	0.9315
12.4	<0.0001	0.0001	<0.0001	<0.0001	<0.0001	0.7309
18	<0.0001	<0.0001	<0.0001	<0.0001	<0.0001	0.2766
24	<0.0001	<0.0001	<0.0001	0.3579	0.7995	0.406
30.2	<0.0001	<0.0001	<0.0001	0.6535	>0.9999	0.6535
35	0.0002	0.004	0.0004	0.9589	>0.9999	0.9589

EV5A (Traveled distance)
Adjusted P Value

Clic5 $^{+/-}$ vs. Clic5 $^{-/-}$	Clic5 $^{+/-}$ vs. Clic5 $^{-/-}$ +ssAAV	Clic5 $^{+/-}$ vs. Clic5 $^{-/-}$ +scAAV	Clic5 $^{-/-}$ vs. Clic5 $^{-/-}$ +ssAAV	Clic5 $^{-/-}$ vs. Clic5 $^{-/-}$ +scAAV	Clic5 $^{-/-}$ +ssAAV vs. Clic5 $^{-/-}$ +scAAV
<0.0001	>0.9999	0.8067	<0.0001	<0.0001	0.8695

EV5B (Circling behavior)
Adjusted P Value

Clic5 $^{+/-}$ vs. Clic5 $^{-/-}$	Clic5 $^{+/-}$ vs. Clic5 $^{-/-}$ +ssAAV	Clic5 $^{+/-}$ vs. Clic5 $^{-/-}$ +scAAV	Clic5 $^{-/-}$ vs. Clic5 $^{-/-}$ +ssAAV	Clic5 $^{-/-}$ vs. Clic5 $^{-/-}$ +scAAV	Clic5 $^{-/-}$ +ssAAV vs. Clic5 $^{-/-}$ +scAAV
<0.0001	0.9989	0.9997	0.0002	<0.0001	0.9962

EV5C (Rotarod)
Adjusted P Value

Clic5 $^{+/-}$ vs. Clic5 $^{-/-}$	Clic5 $^{+/-}$ vs. Clic5 $^{-/-}$ +ssAAV	Clic5 $^{+/-}$ vs. Clic5 $^{-/-}$ +scAAV	Clic5 $^{-/-}$ vs. Clic5 $^{-/-}$ +ssAAV	Clic5 $^{-/-}$ vs. Clic5 $^{-/-}$ +scAAV	Clic5 $^{-/-}$ +ssAAV vs. Clic5 $^{-/-}$ +scAAV
0.0145	>0.9999	0.9677	0.0181	0.0047	0.974
