# Supplementary figures and images for: AAV gene therapy rescues hearing and balance in a model of CLIC5 deafness
Source: EMBO Mol Med. 2025 Aug 26;17(9):2233–57. doi: 10.1038/s44321-025-00275-7 (PMC12423326; doi:10.1038/s44321-025-00275-7)

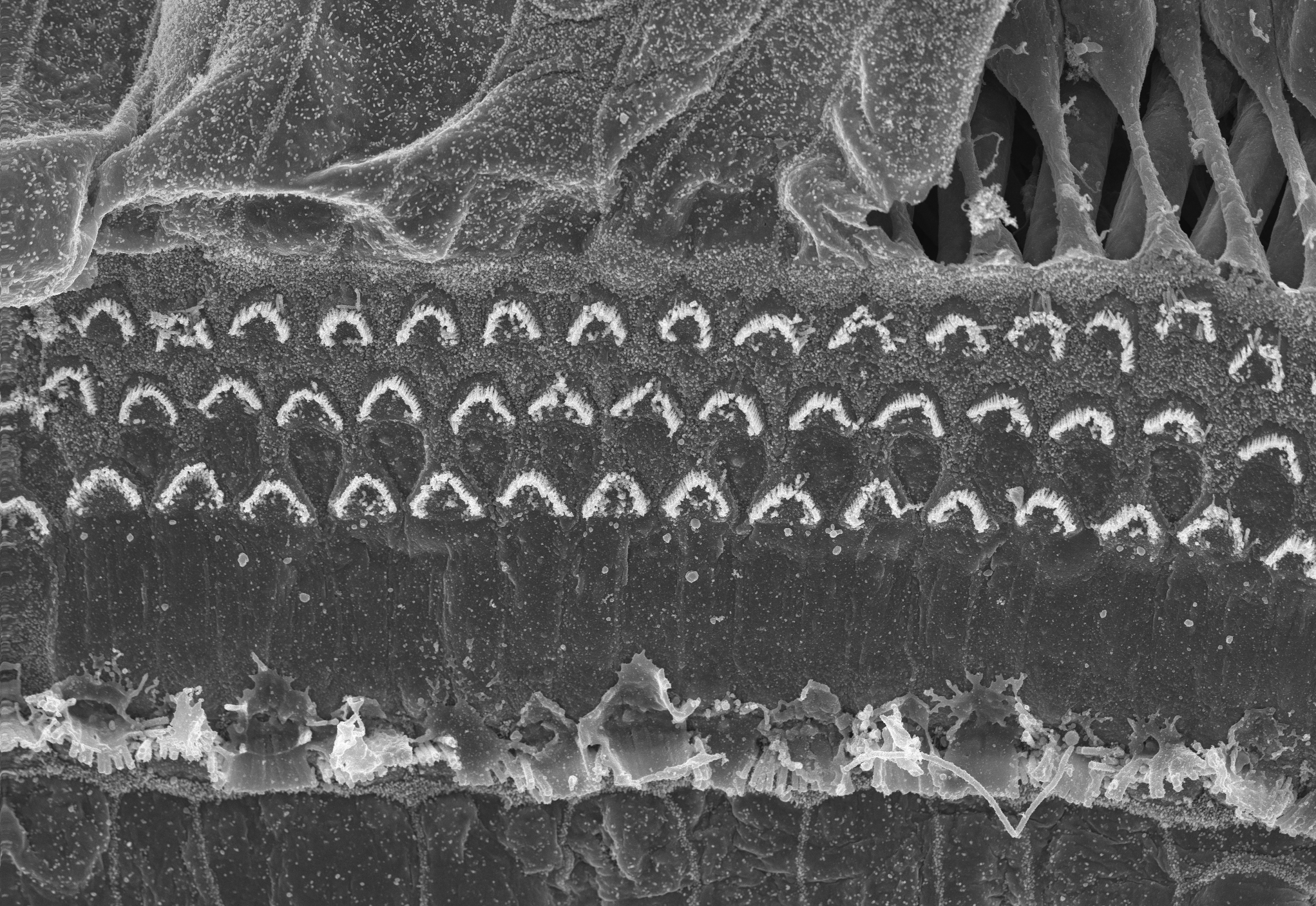

Supplement: Supplementary file 4 — Source data Fig. 1 [file 44321_2025_275_MOESM4_ESM.zip › Manuscript_EMM-2025-21431_SourceDataForFigure1/1H/Middle.tif]

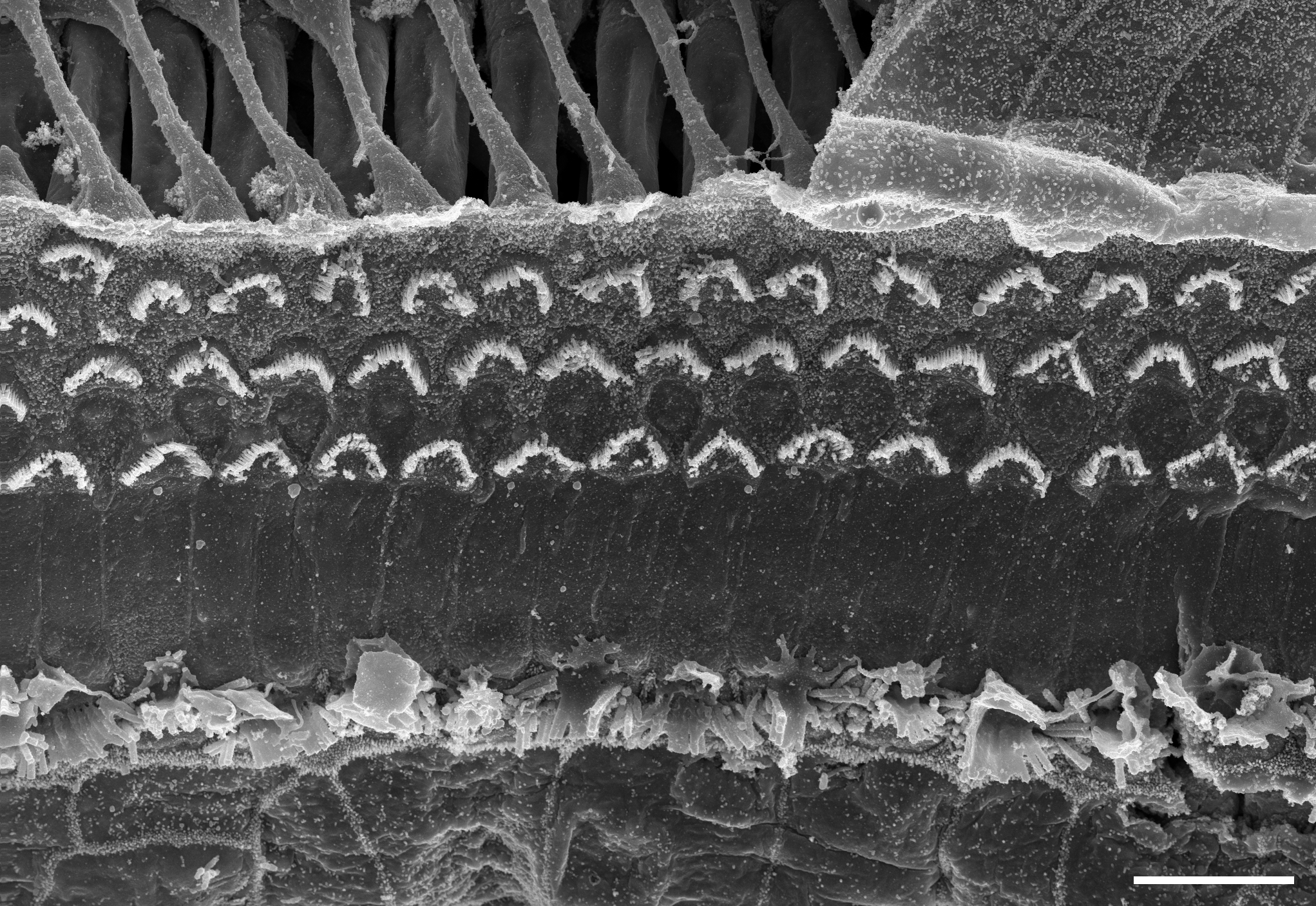

Supplement: Supplementary file 4 — Source data Fig. 1 [file 44321_2025_275_MOESM4_ESM.zip › Manuscript_EMM-2025-21431_SourceDataForFigure1/1H/Base.tif]

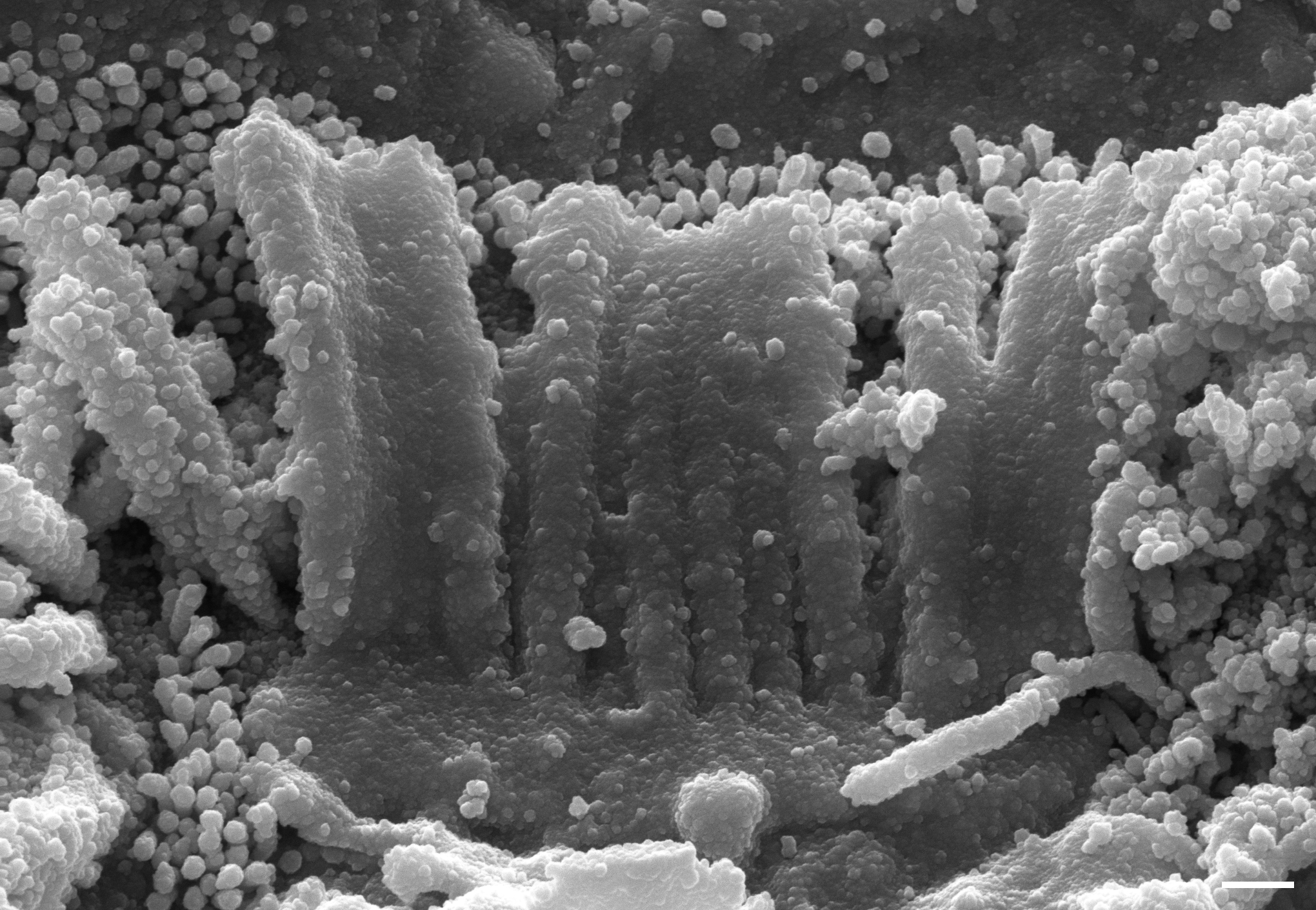

Supplement: Supplementary file 4 — Source data Fig. 1 [file 44321_2025_275_MOESM4_ESM.zip › Manuscript_EMM-2025-21431_SourceDataForFigure1/1I/IHC_p17.tif]

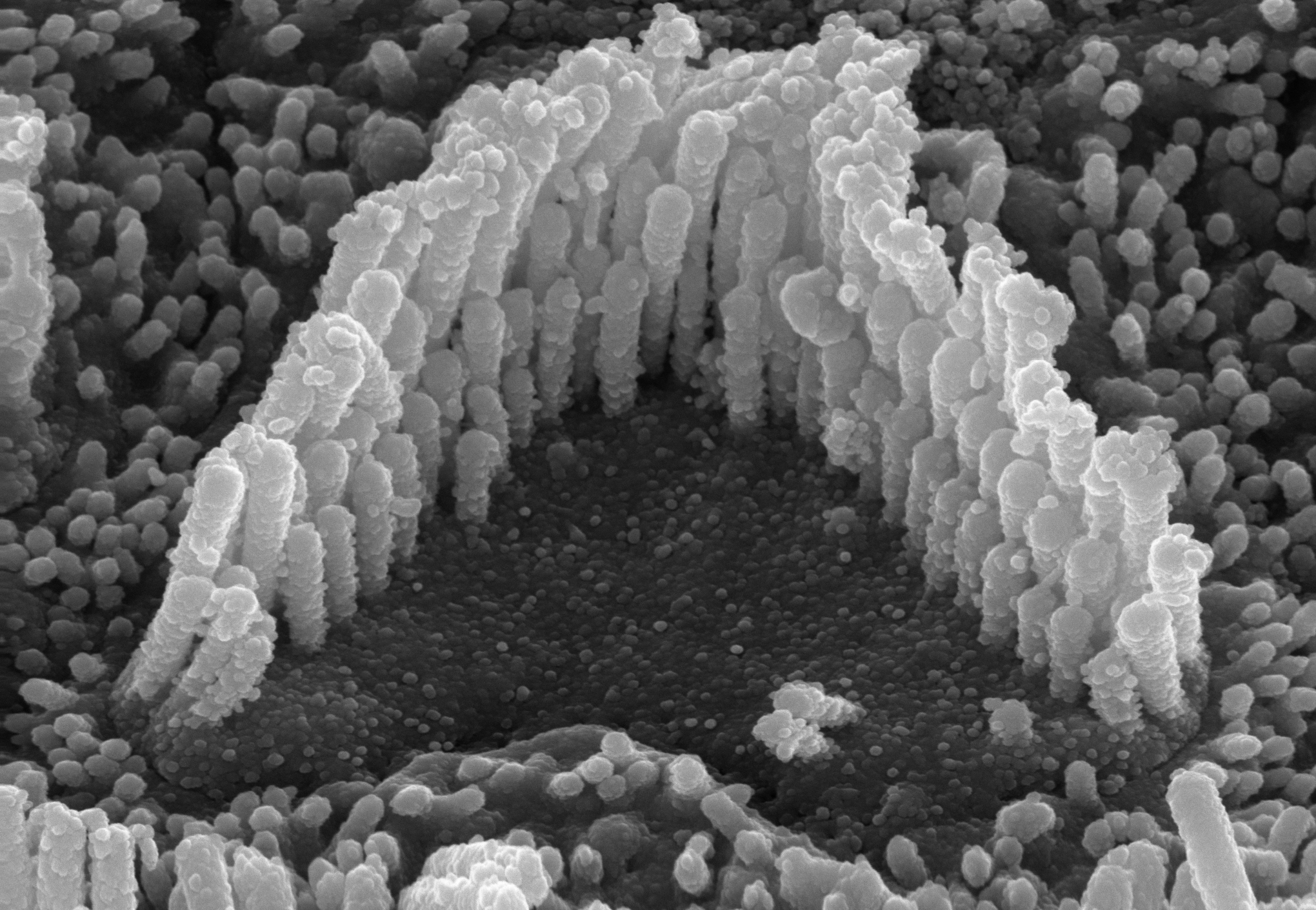

Supplement: Supplementary file 4 — Source data Fig. 1 [file 44321_2025_275_MOESM4_ESM.zip › Manuscript_EMM-2025-21431_SourceDataForFigure1/1I/OHC_p14.tif]

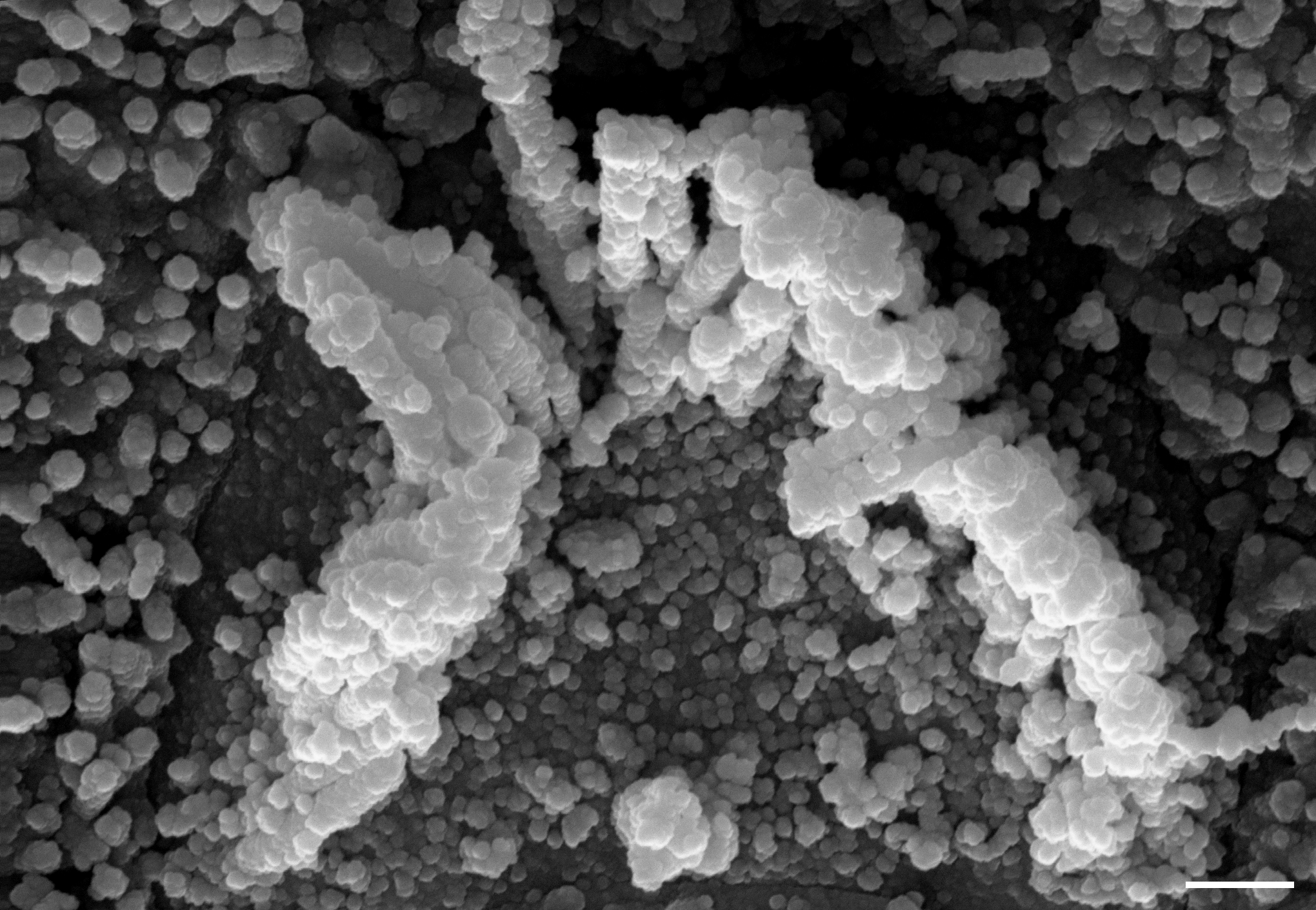

Supplement: Supplementary file 4 — Source data Fig. 1 [file 44321_2025_275_MOESM4_ESM.zip › Manuscript_EMM-2025-21431_SourceDataForFigure1/1I/OHC_p17.tif]

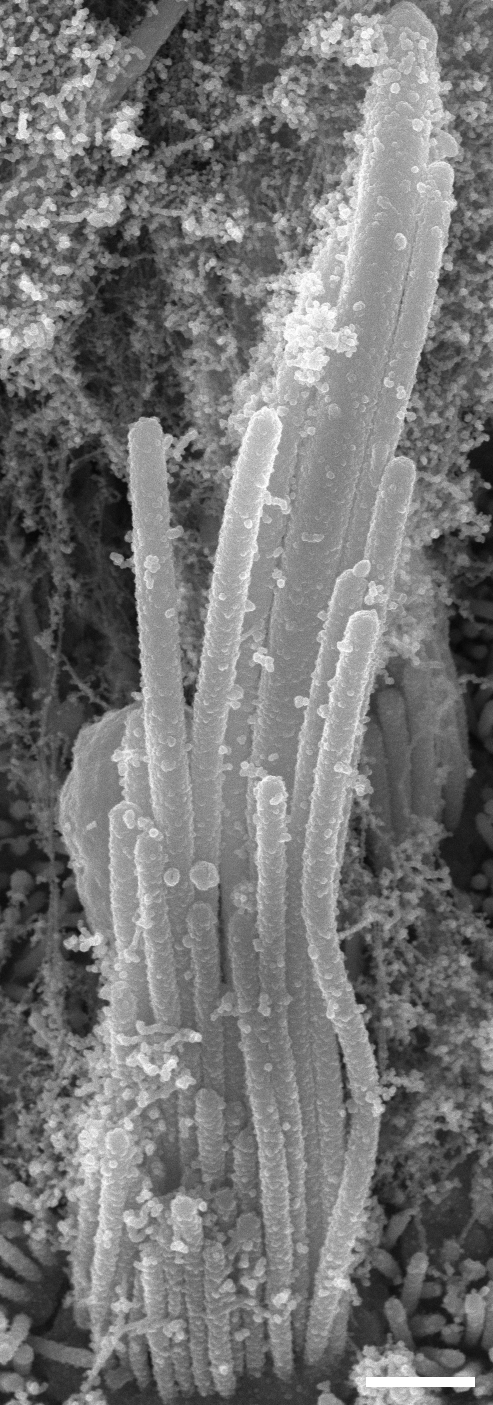

Supplement: Supplementary file 4 — Source data Fig. 1 [file 44321_2025_275_MOESM4_ESM.zip › Manuscript_EMM-2025-21431_SourceDataForFigure1/1J/VHC_p17.tif]

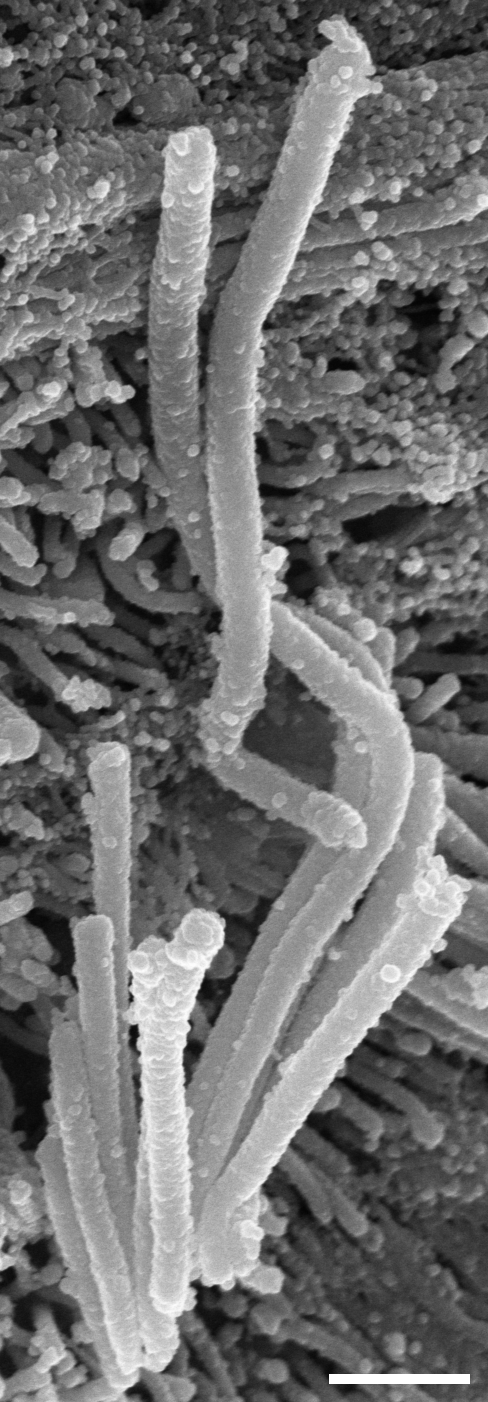

Supplement: Supplementary file 4 — Source data Fig. 1 [file 44321_2025_275_MOESM4_ESM.zip › Manuscript_EMM-2025-21431_SourceDataForFigure1/1J/VHC_p15.tif]

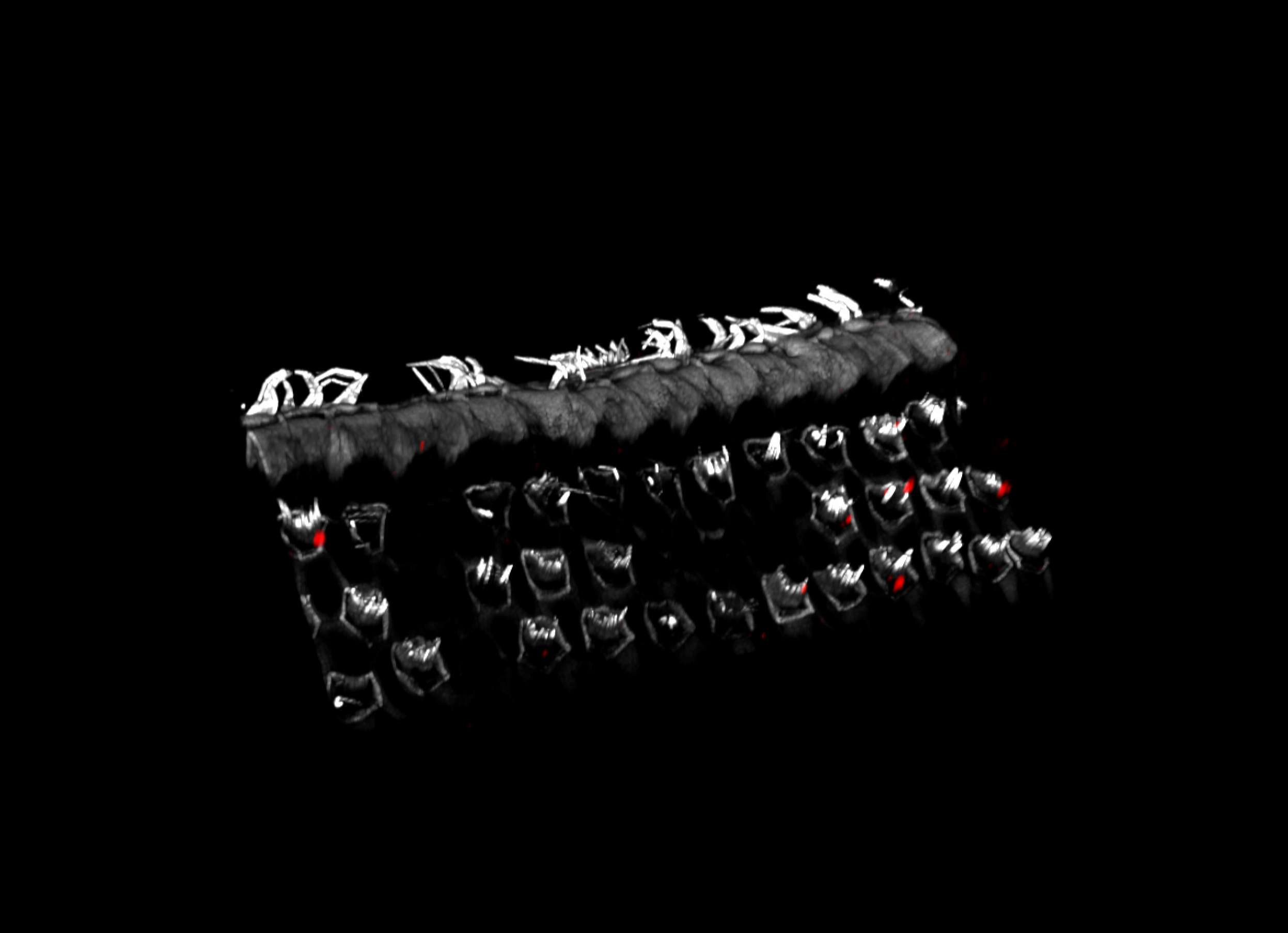

Supplement: Supplementary file 5 — Source data Fig. 2 [file 44321_2025_275_MOESM5_ESM.zip › Manuscript_EMM-2025-21431_SourceDataForFigure2/2A/Hom.tif]

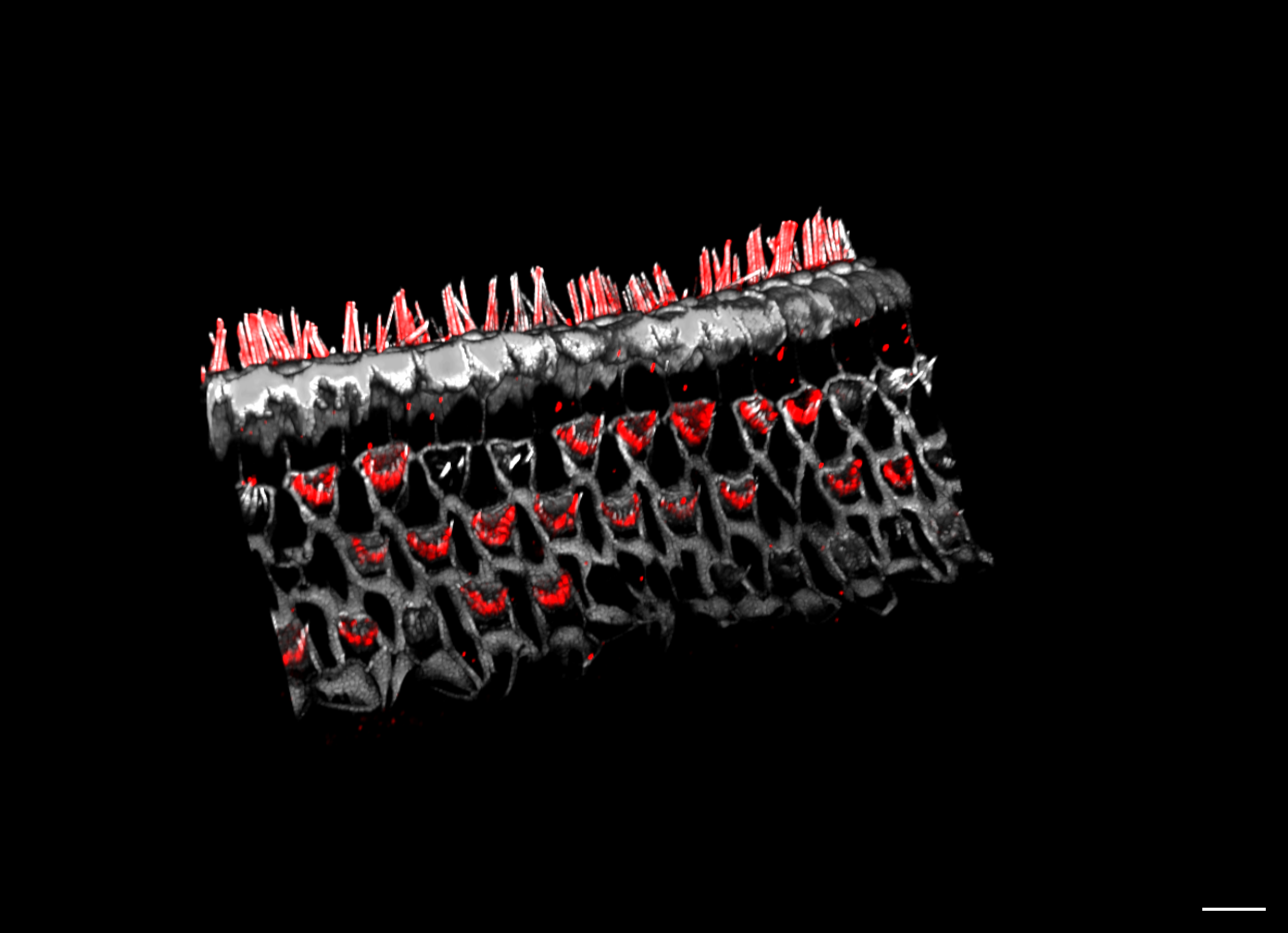

Supplement: Supplementary file 5 — Source data Fig. 2 [file 44321_2025_275_MOESM5_ESM.zip › Manuscript_EMM-2025-21431_SourceDataForFigure2/2A/Hom +ssAAV Clic5.tif]

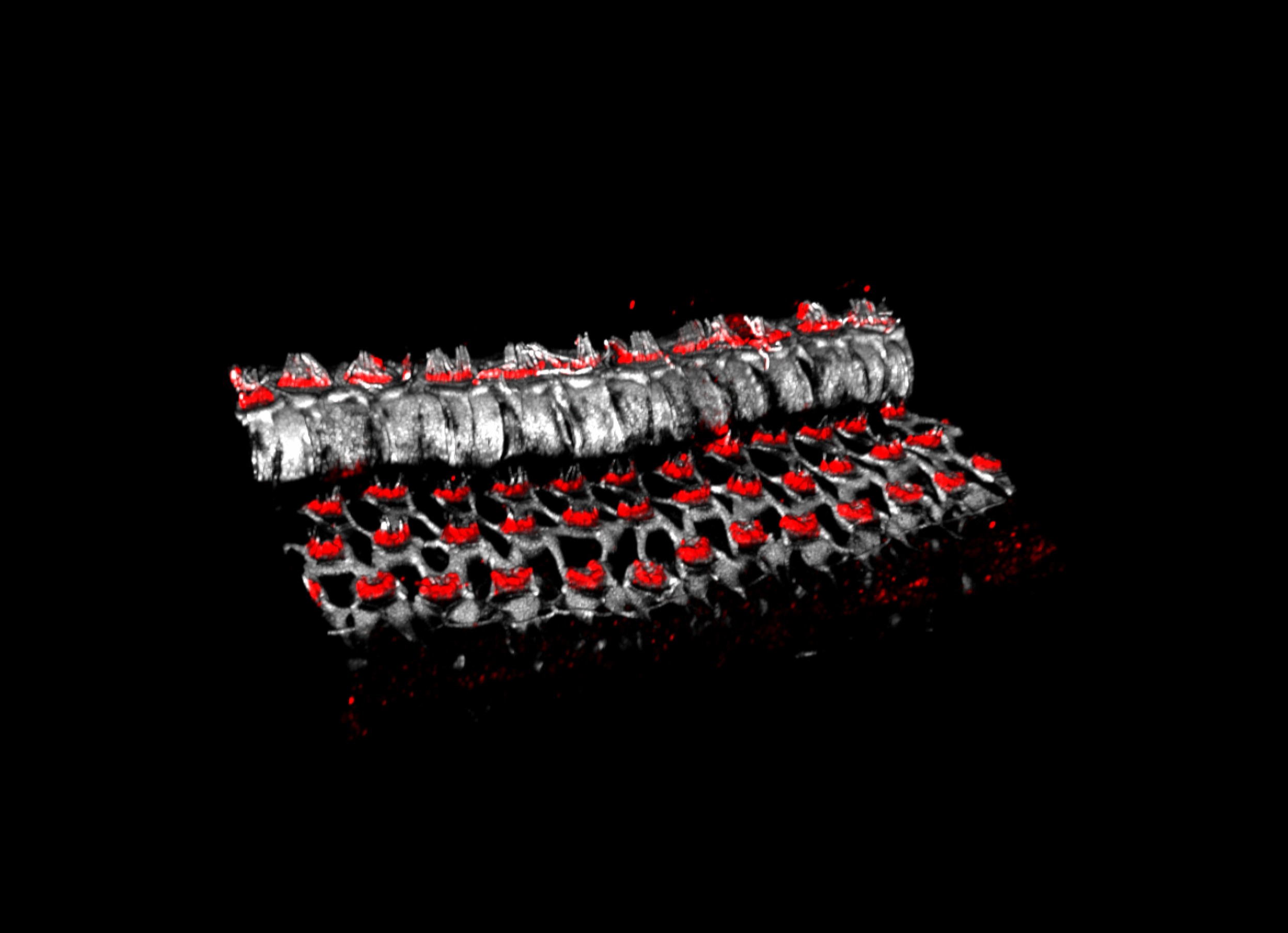

Supplement: Supplementary file 5 — Source data Fig. 2 [file 44321_2025_275_MOESM5_ESM.zip › Manuscript_EMM-2025-21431_SourceDataForFigure2/2A/Het.tif]

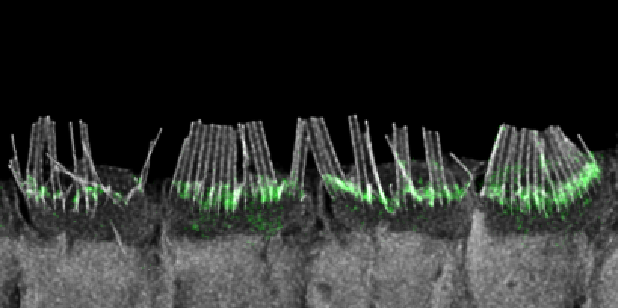

Supplement: Supplementary file 5 — Source data Fig. 2 [file 44321_2025_275_MOESM5_ESM.zip › Manuscript_EMM-2025-21431_SourceDataForFigure2/2C/Hom ssAAV_Flag.tif]

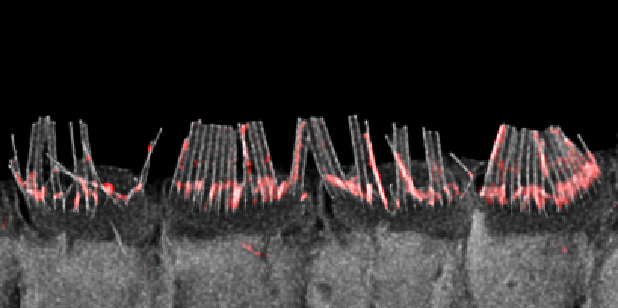

Supplement: Supplementary file 5 — Source data Fig. 2 [file 44321_2025_275_MOESM5_ESM.zip › Manuscript_EMM-2025-21431_SourceDataForFigure2/2C/Hom ssAAV_Clic5.tif]

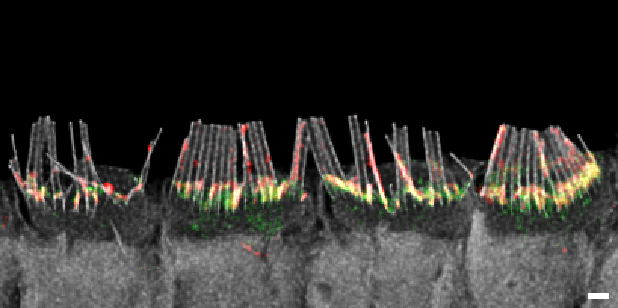

Supplement: Supplementary file 5 — Source data Fig. 2 [file 44321_2025_275_MOESM5_ESM.zip › Manuscript_EMM-2025-21431_SourceDataForFigure2/2C/Hom ssAAV_merge.tif]

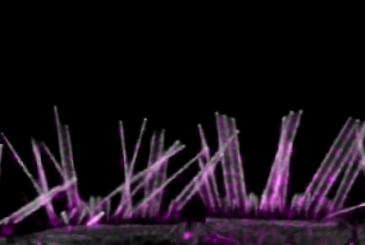

Supplement: Supplementary file 5 — Source data Fig. 2 [file 44321_2025_275_MOESM5_ESM.zip › Manuscript_EMM-2025-21431_SourceDataForFigure2/2D/WT_RDX.tif]

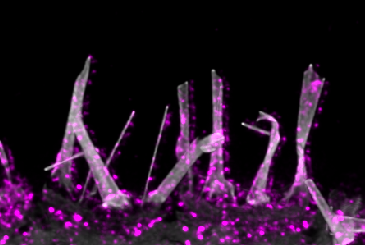

Supplement: Supplementary file 5 — Source data Fig. 2 [file 44321_2025_275_MOESM5_ESM.zip › Manuscript_EMM-2025-21431_SourceDataForFigure2/2D/Hom_RDX.tif]

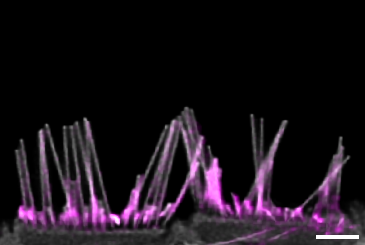

Supplement: Supplementary file 5 — Source data Fig. 2 [file 44321_2025_275_MOESM5_ESM.zip › Manuscript_EMM-2025-21431_SourceDataForFigure2/2D/Hom+ssAAV_RDX.tif]

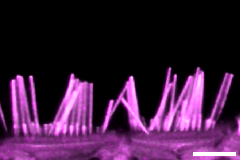

Supplement: Supplementary file 5 — Source data Fig. 2 [file 44321_2025_275_MOESM5_ESM.zip › Manuscript_EMM-2025-21431_SourceDataForFigure2/2E/Hom +ssAAV_TPRN.tif]

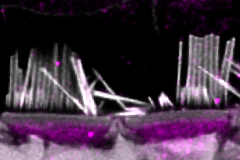

Supplement: Supplementary file 5 — Source data Fig. 2 [file 44321_2025_275_MOESM5_ESM.zip › Manuscript_EMM-2025-21431_SourceDataForFigure2/2E/WT_TPRN.tif]

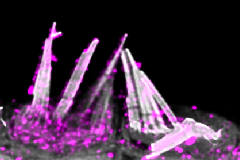

Supplement: Supplementary file 5 — Source data Fig. 2 [file 44321_2025_275_MOESM5_ESM.zip › Manuscript_EMM-2025-21431_SourceDataForFigure2/2E/Hom_TPRN.tif]

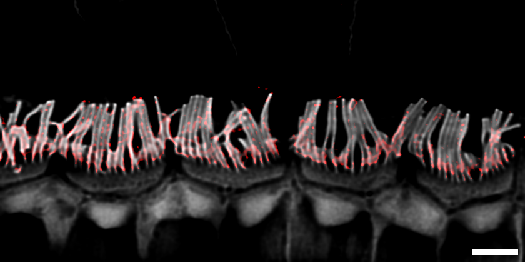

Supplement: Supplementary file 5 — Source data Fig. 2 [file 44321_2025_275_MOESM5_ESM.zip › Manuscript_EMM-2025-21431_SourceDataForFigure2/2B/Het_Clic5.tif]

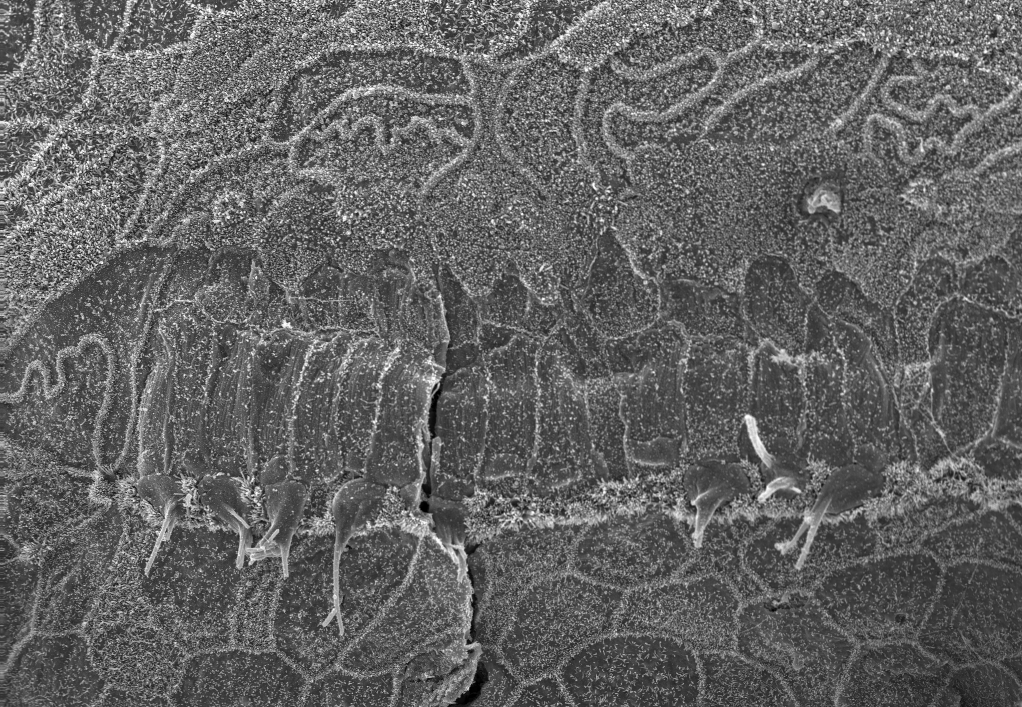

Supplement: Supplementary file 6 — Source data Fig. 3 [file 44321_2025_275_MOESM6_ESM.zip › Manuscript_EMM-2025-21431_SourceDataForFigure3/3E/Hom_Base.tif]

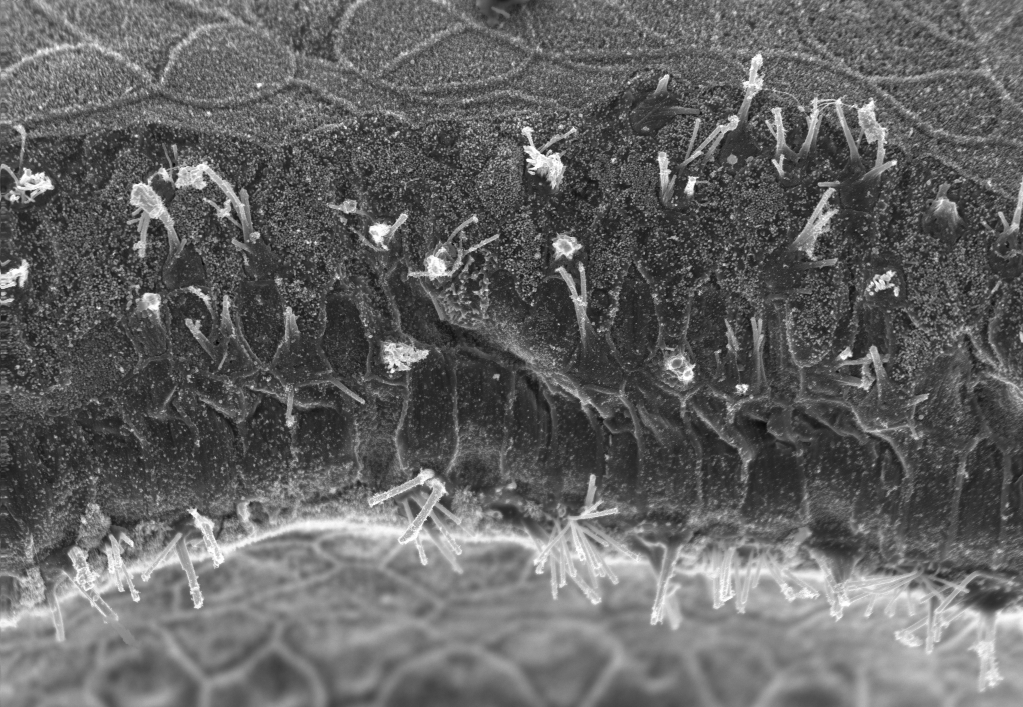

Supplement: Supplementary file 6 — Source data Fig. 3 [file 44321_2025_275_MOESM6_ESM.zip › Manuscript_EMM-2025-21431_SourceDataForFigure3/3E/Hom_Apex.tif]

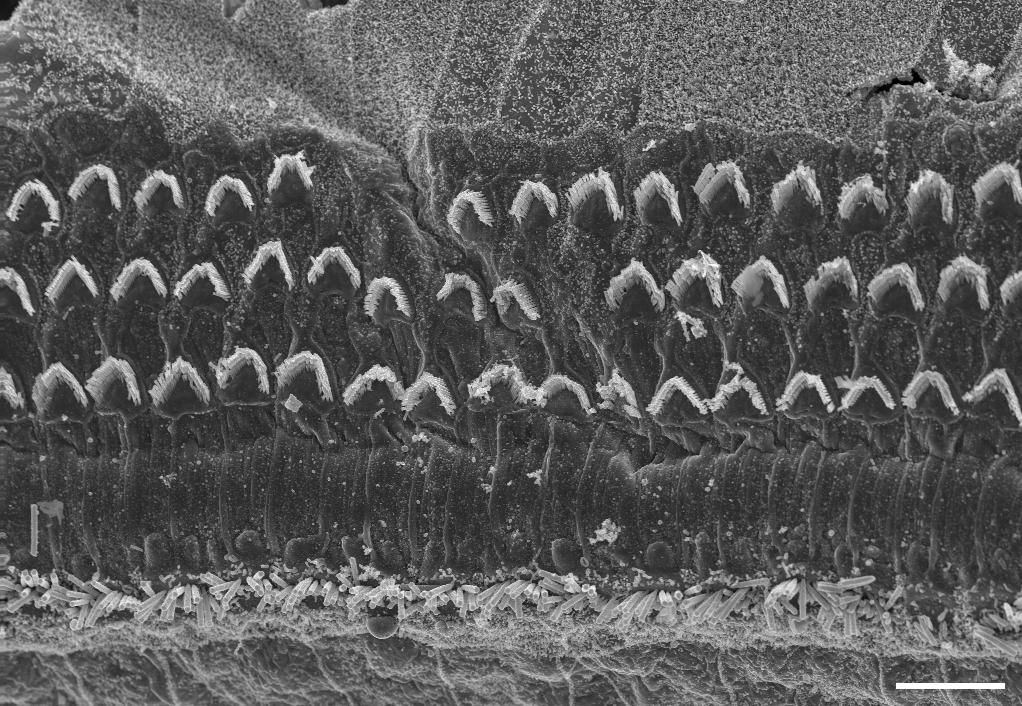

Supplement: Supplementary file 6 — Source data Fig. 3 [file 44321_2025_275_MOESM6_ESM.zip › Manuscript_EMM-2025-21431_SourceDataForFigure3/3E/Hom+ ssAAV_Base.tif]

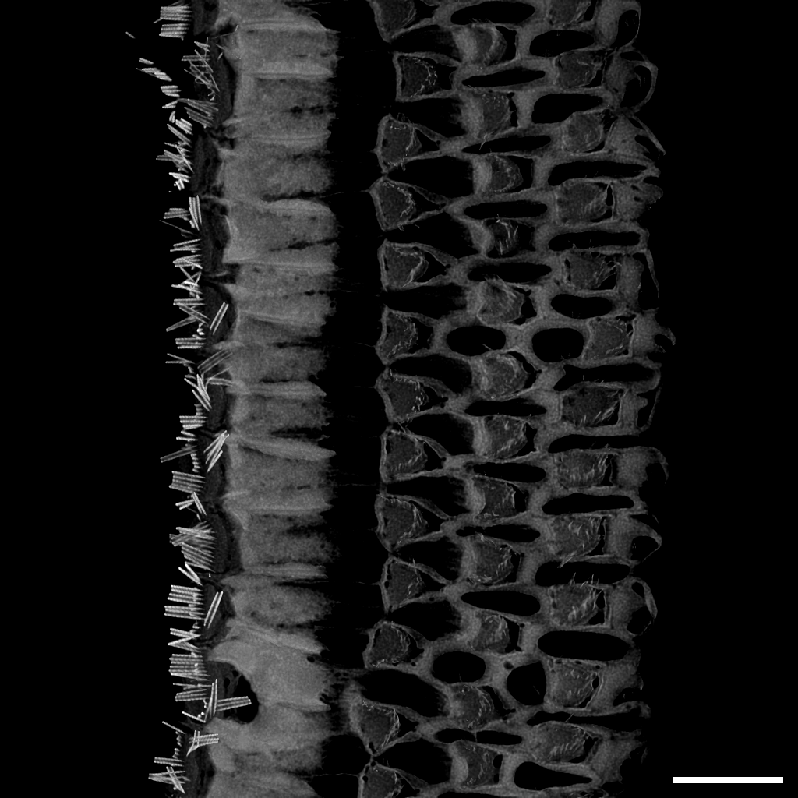

Supplement: Supplementary file 6 — Source data Fig. 3 [file 44321_2025_275_MOESM6_ESM.zip › Manuscript_EMM-2025-21431_SourceDataForFigure3/3A/Hom + ssAAV.tif]

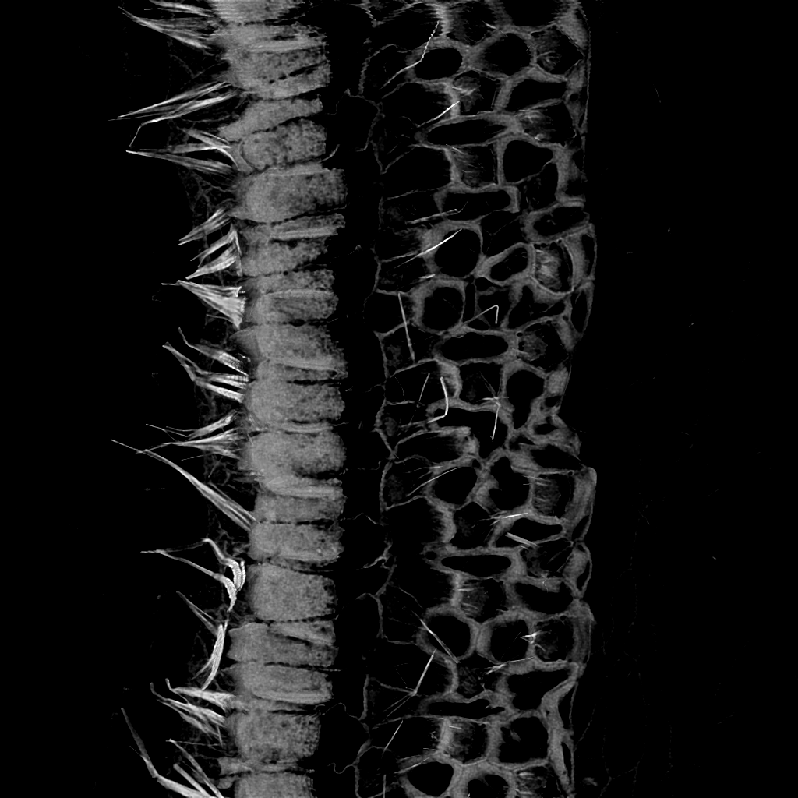

Supplement: Supplementary file 6 — Source data Fig. 3 [file 44321_2025_275_MOESM6_ESM.zip › Manuscript_EMM-2025-21431_SourceDataForFigure3/3A/Hom.tif]

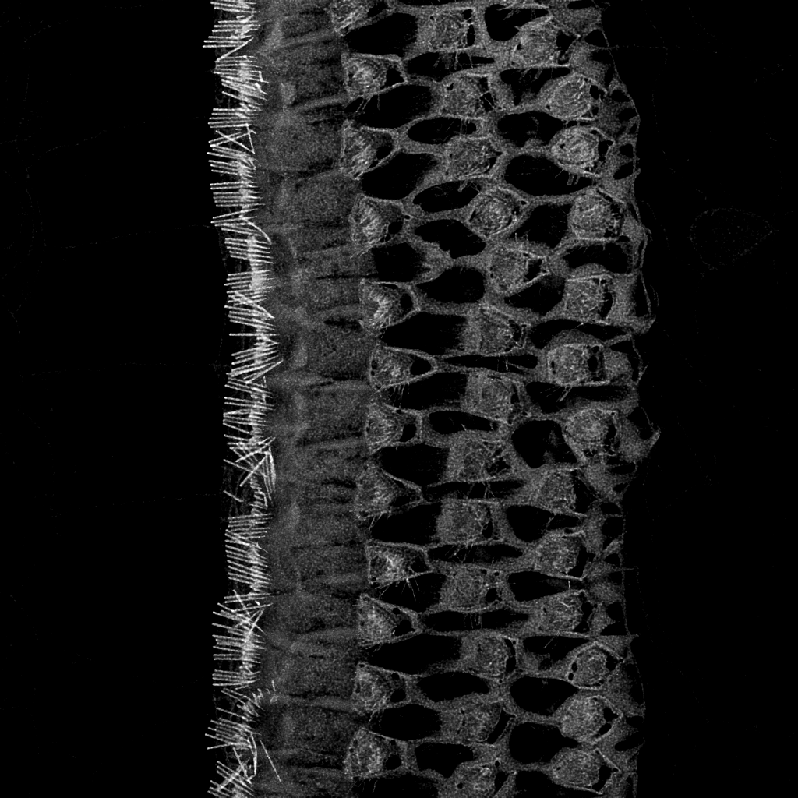

Supplement: Supplementary file 6 — Source data Fig. 3 [file 44321_2025_275_MOESM6_ESM.zip › Manuscript_EMM-2025-21431_SourceDataForFigure3/3A/Het.tif]

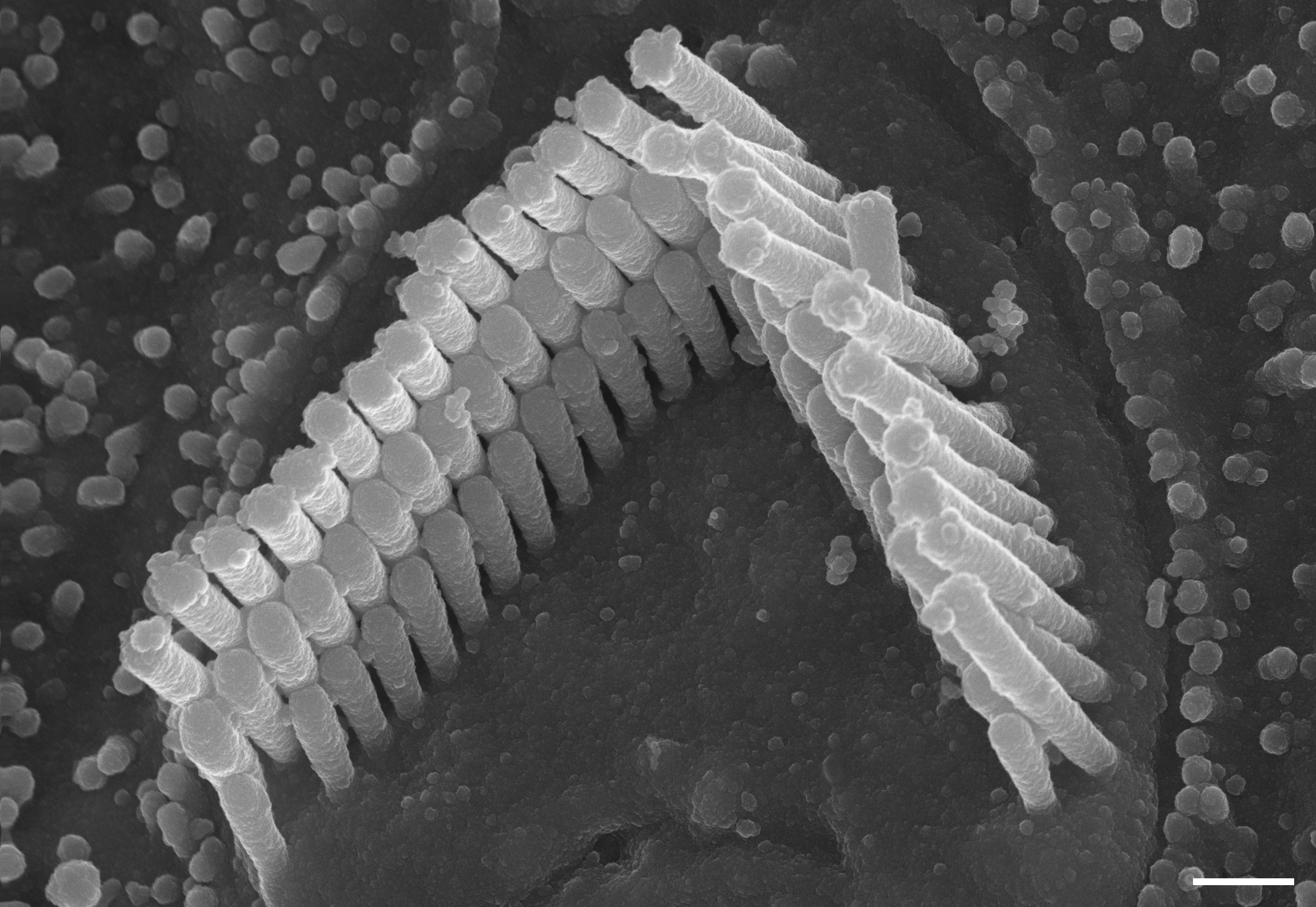

Supplement: Supplementary file 6 — Source data Fig. 3 [file 44321_2025_275_MOESM6_ESM.zip › Manuscript_EMM-2025-21431_SourceDataForFigure3/3F/OHC_Hom+ ssAAV.tif]

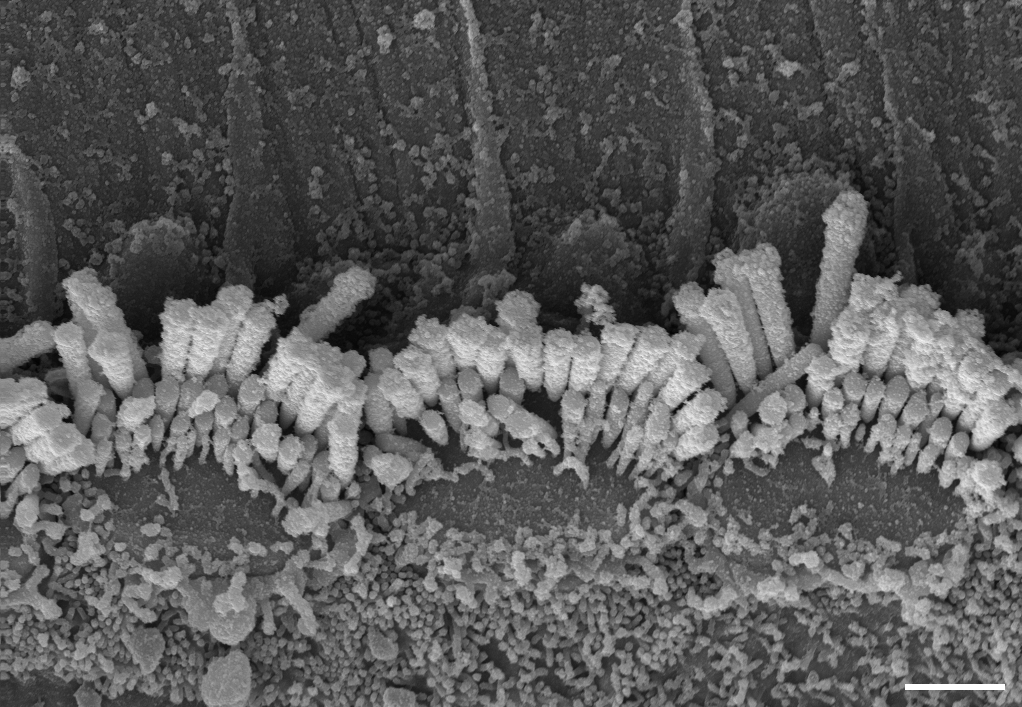

Supplement: Supplementary file 6 — Source data Fig. 3 [file 44321_2025_275_MOESM6_ESM.zip › Manuscript_EMM-2025-21431_SourceDataForFigure3/3F/IHC_Hom+ ssAAV.tif]

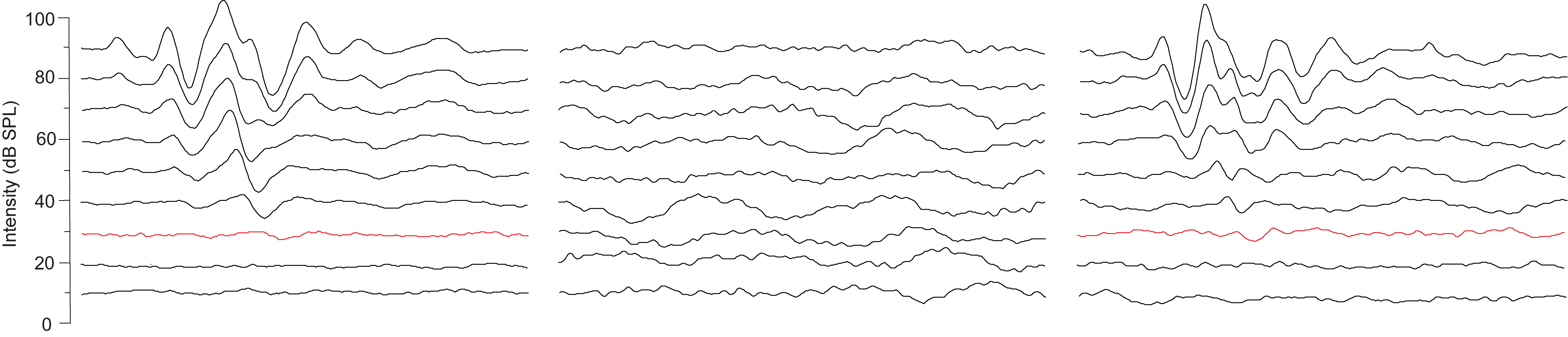

Supplement: Supplementary file 7 — Source data Fig. 4 [file 44321_2025_275_MOESM7_ESM.zip › Manuscript_EMM-2025-21431_SourceDataForFigure4/4A/ABR waves.tif]

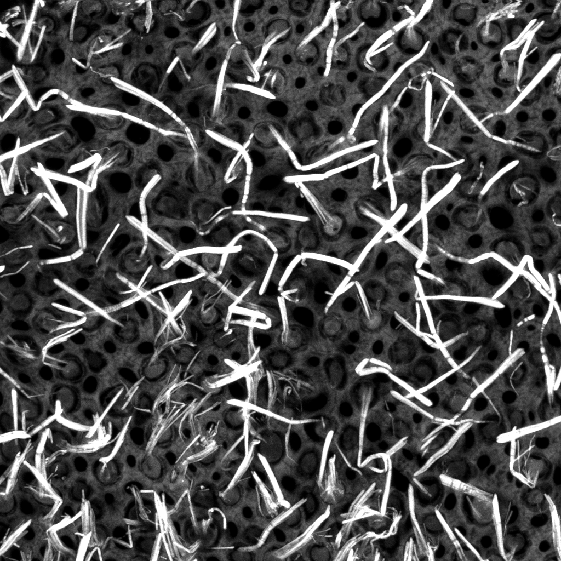

Supplement: Supplementary file 8 — Source data Fig. 5 [file 44321_2025_275_MOESM8_ESM.zip › Manuscript_EMM-2025-21431_SourceDataForFigure5/5A/Hom.tif]

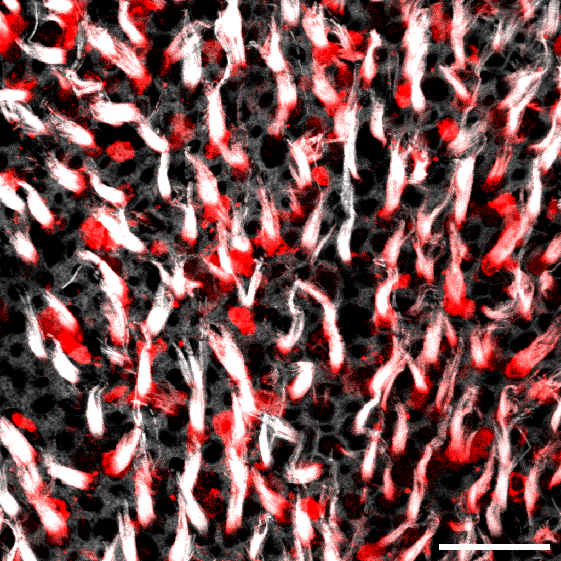

Supplement: Supplementary file 8 — Source data Fig. 5 [file 44321_2025_275_MOESM8_ESM.zip › Manuscript_EMM-2025-21431_SourceDataForFigure5/5A/Hom+ ssAAV.tif]

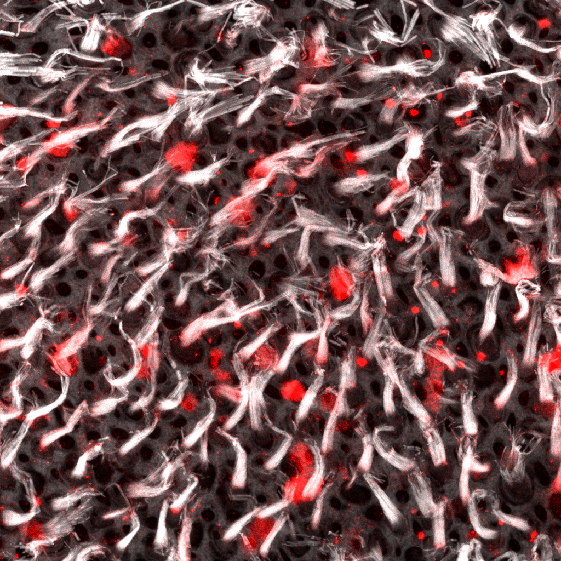

Supplement: Supplementary file 8 — Source data Fig. 5 [file 44321_2025_275_MOESM8_ESM.zip › Manuscript_EMM-2025-21431_SourceDataForFigure5/5A/Het.tif]

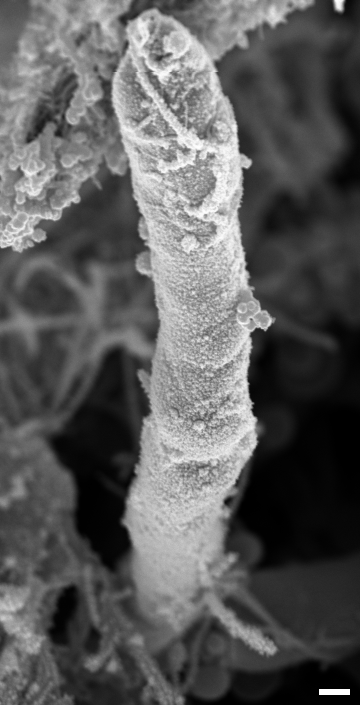

Supplement: Supplementary file 8 — Source data Fig. 5 [file 44321_2025_275_MOESM8_ESM.zip › Manuscript_EMM-2025-21431_SourceDataForFigure5/5D/Hom.tif]

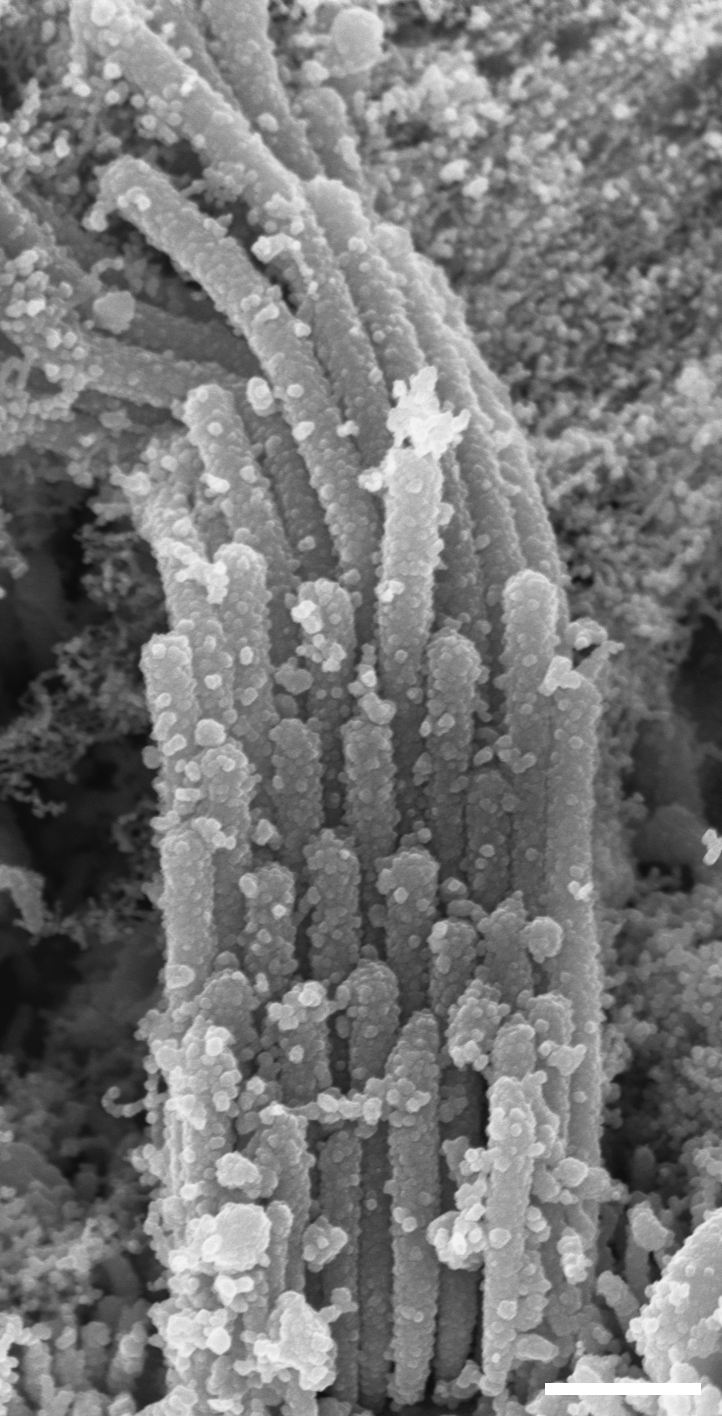

Supplement: Supplementary file 8 — Source data Fig. 5 [file 44321_2025_275_MOESM8_ESM.zip › Manuscript_EMM-2025-21431_SourceDataForFigure5/5D/Hom+ ssAAV.tif]

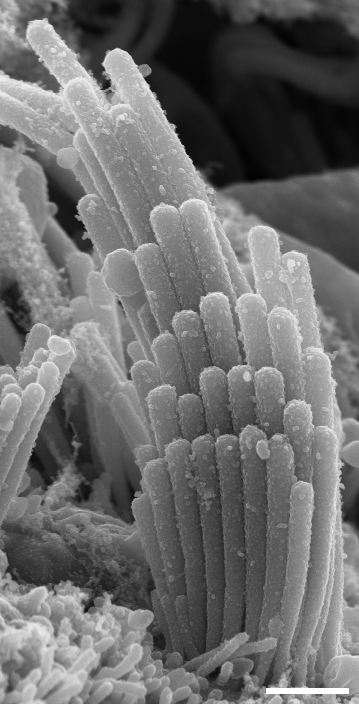

Supplement: Supplementary file 8 — Source data Fig. 5 [file 44321_2025_275_MOESM8_ESM.zip › Manuscript_EMM-2025-21431_SourceDataForFigure5/5D/Het.tif]

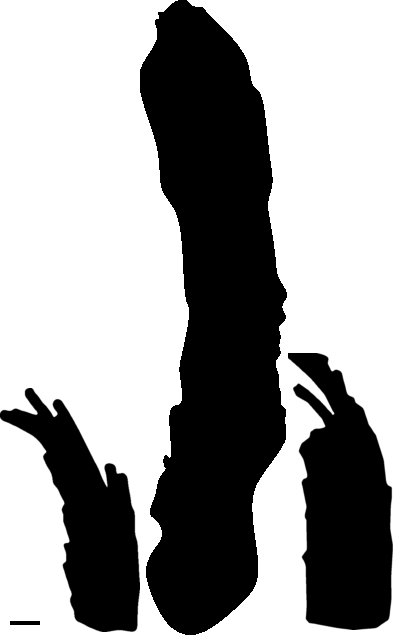

Supplement: Supplementary file 8 — Source data Fig. 5 [file 44321_2025_275_MOESM8_ESM.zip › Manuscript_EMM-2025-21431_SourceDataForFigure5/5E/5E.tif]

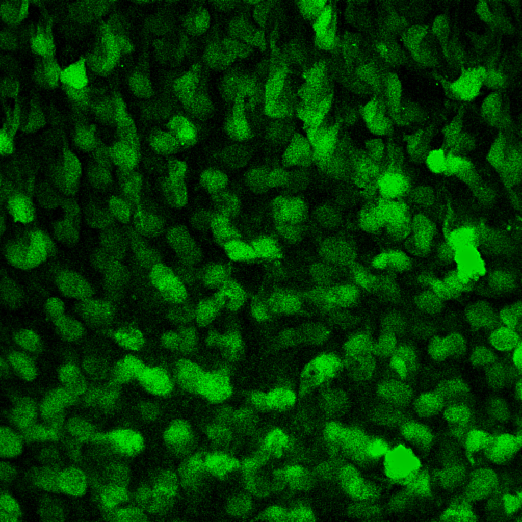

Supplement: Supplementary file 9 — Source data Fig. 6 [file 44321_2025_275_MOESM9_ESM.zip › Manuscript_EMM-2025-21431_SourceDataForFigure6/6A/scAAV.GFP_gfp.tif]

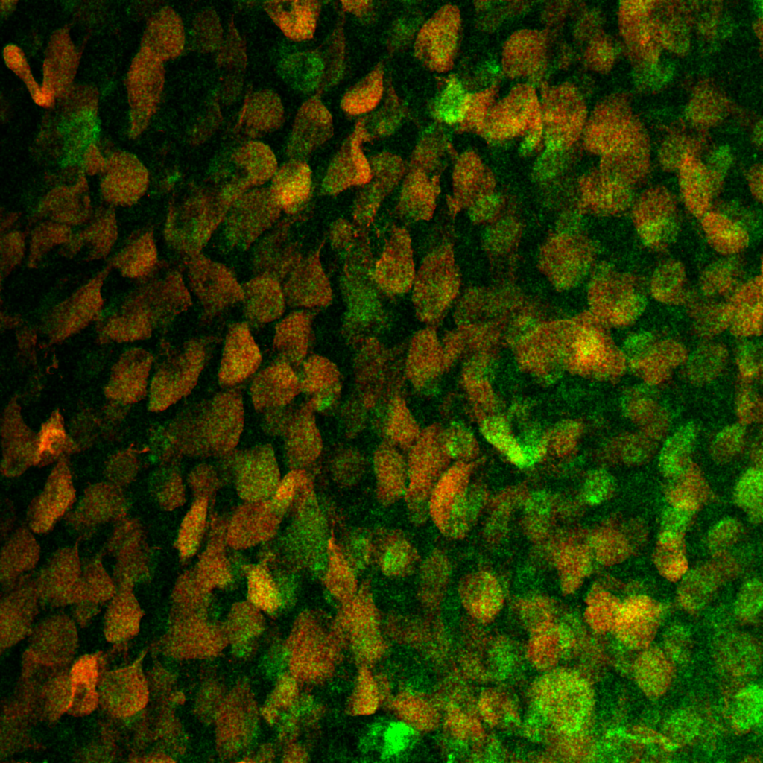

Supplement: Supplementary file 9 — Source data Fig. 6 [file 44321_2025_275_MOESM9_ESM.zip › Manuscript_EMM-2025-21431_SourceDataForFigure6/6A/ssAAV.GFP_merge.tif]

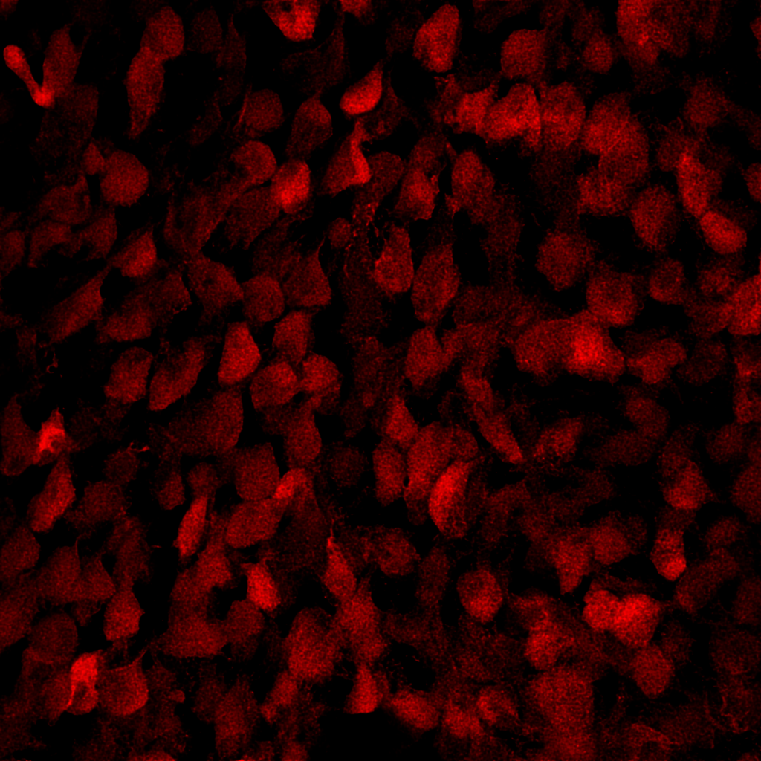

Supplement: Supplementary file 9 — Source data Fig. 6 [file 44321_2025_275_MOESM9_ESM.zip › Manuscript_EMM-2025-21431_SourceDataForFigure6/6A/ssAAV.GFP_myo7a.tif]

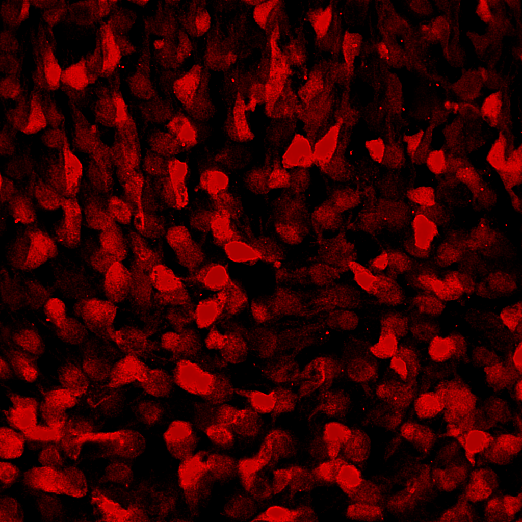

Supplement: Supplementary file 9 — Source data Fig. 6 [file 44321_2025_275_MOESM9_ESM.zip › Manuscript_EMM-2025-21431_SourceDataForFigure6/6A/scAAV.GFP_myo7a.tif]

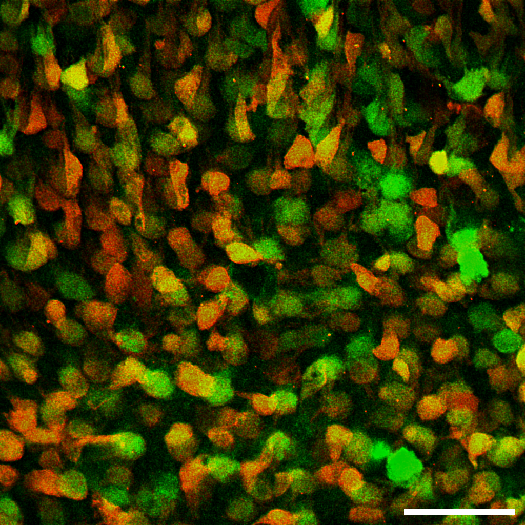

Supplement: Supplementary file 9 — Source data Fig. 6 [file 44321_2025_275_MOESM9_ESM.zip › Manuscript_EMM-2025-21431_SourceDataForFigure6/6A/scAAV.GFP_merge_10 micron scale.tif]

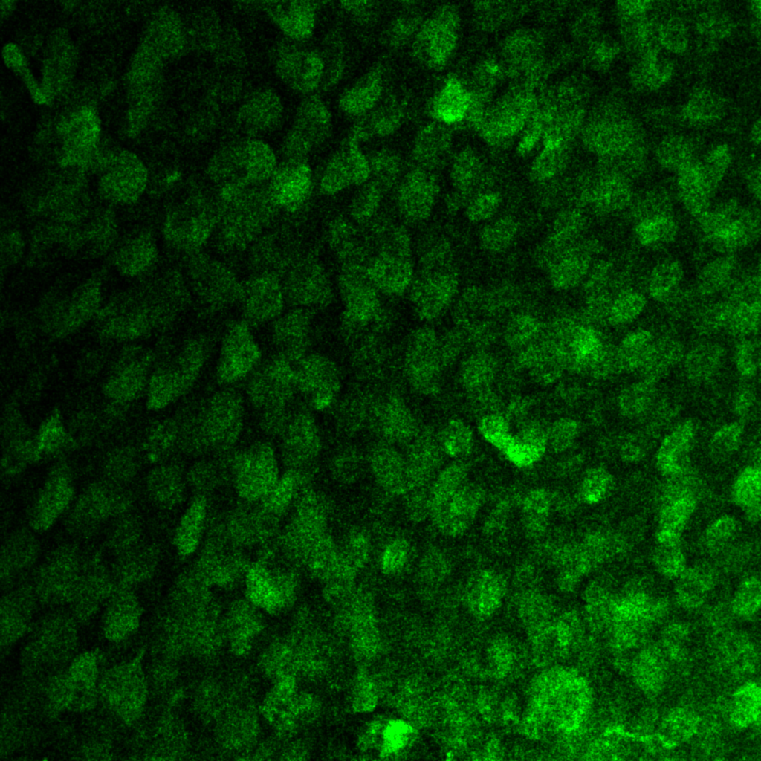

Supplement: Supplementary file 9 — Source data Fig. 6 [file 44321_2025_275_MOESM9_ESM.zip › Manuscript_EMM-2025-21431_SourceDataForFigure6/6A/ssAAV.GFP_gfp.tif]

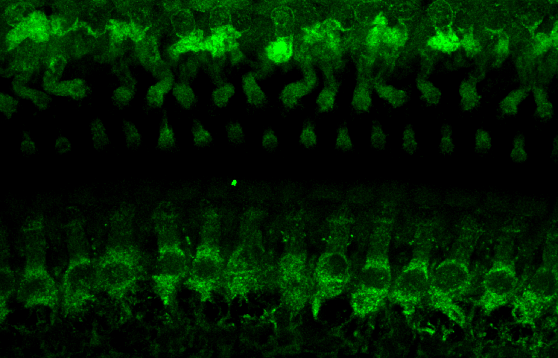

Supplement: Supplementary file 9 — Source data Fig. 6 [file 44321_2025_275_MOESM9_ESM.zip › Manuscript_EMM-2025-21431_SourceDataForFigure6/6D/ssAAV.GFP_16 kHz.tif.tif]

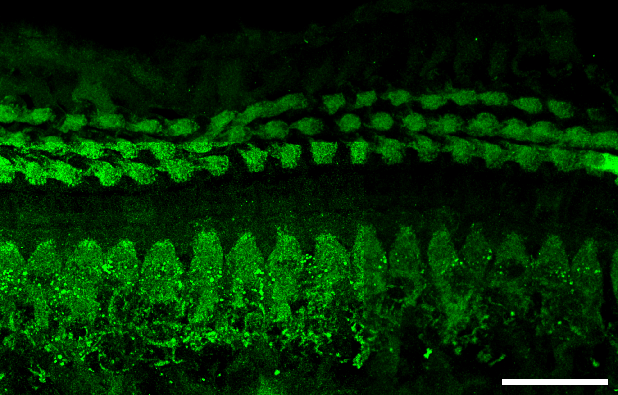

Supplement: Supplementary file 9 — Source data Fig. 6 [file 44321_2025_275_MOESM9_ESM.zip › Manuscript_EMM-2025-21431_SourceDataForFigure6/6D/scAAV.GFP_32 kHz_scale 5 micron.tif]

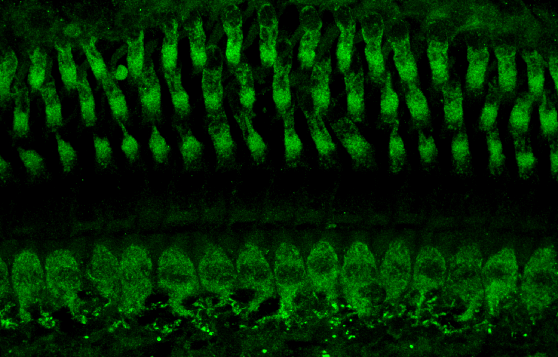

Supplement: Supplementary file 9 — Source data Fig. 6 [file 44321_2025_275_MOESM9_ESM.zip › Manuscript_EMM-2025-21431_SourceDataForFigure6/6D/scAAV.GFP_16 kHz.tif]

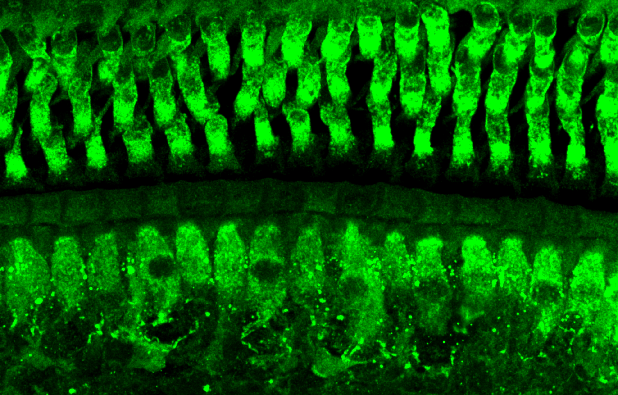

Supplement: Supplementary file 9 — Source data Fig. 6 [file 44321_2025_275_MOESM9_ESM.zip › Manuscript_EMM-2025-21431_SourceDataForFigure6/6D/scAAV.GFP_8 kHz.tif]

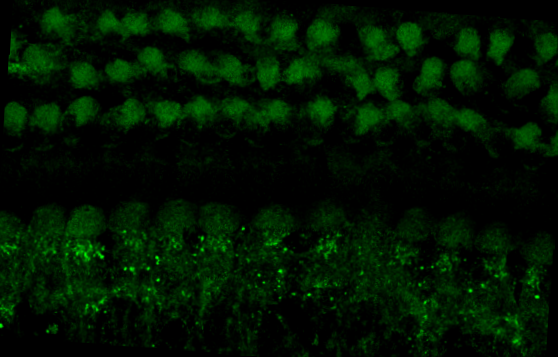

Supplement: Supplementary file 9 — Source data Fig. 6 [file 44321_2025_275_MOESM9_ESM.zip › Manuscript_EMM-2025-21431_SourceDataForFigure6/6D/ssAAV.GFP_8 kHz.tif]

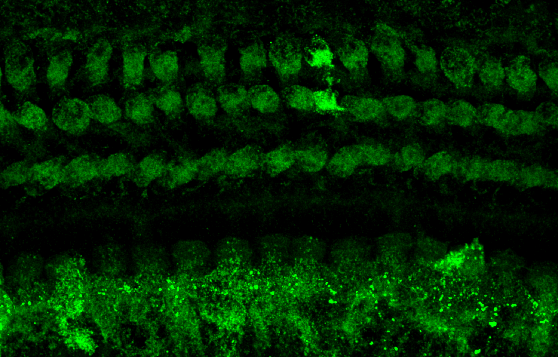

Supplement: Supplementary file 9 — Source data Fig. 6 [file 44321_2025_275_MOESM9_ESM.zip › Manuscript_EMM-2025-21431_SourceDataForFigure6/6D/ssAAV.GFP_32 kHz.tif]

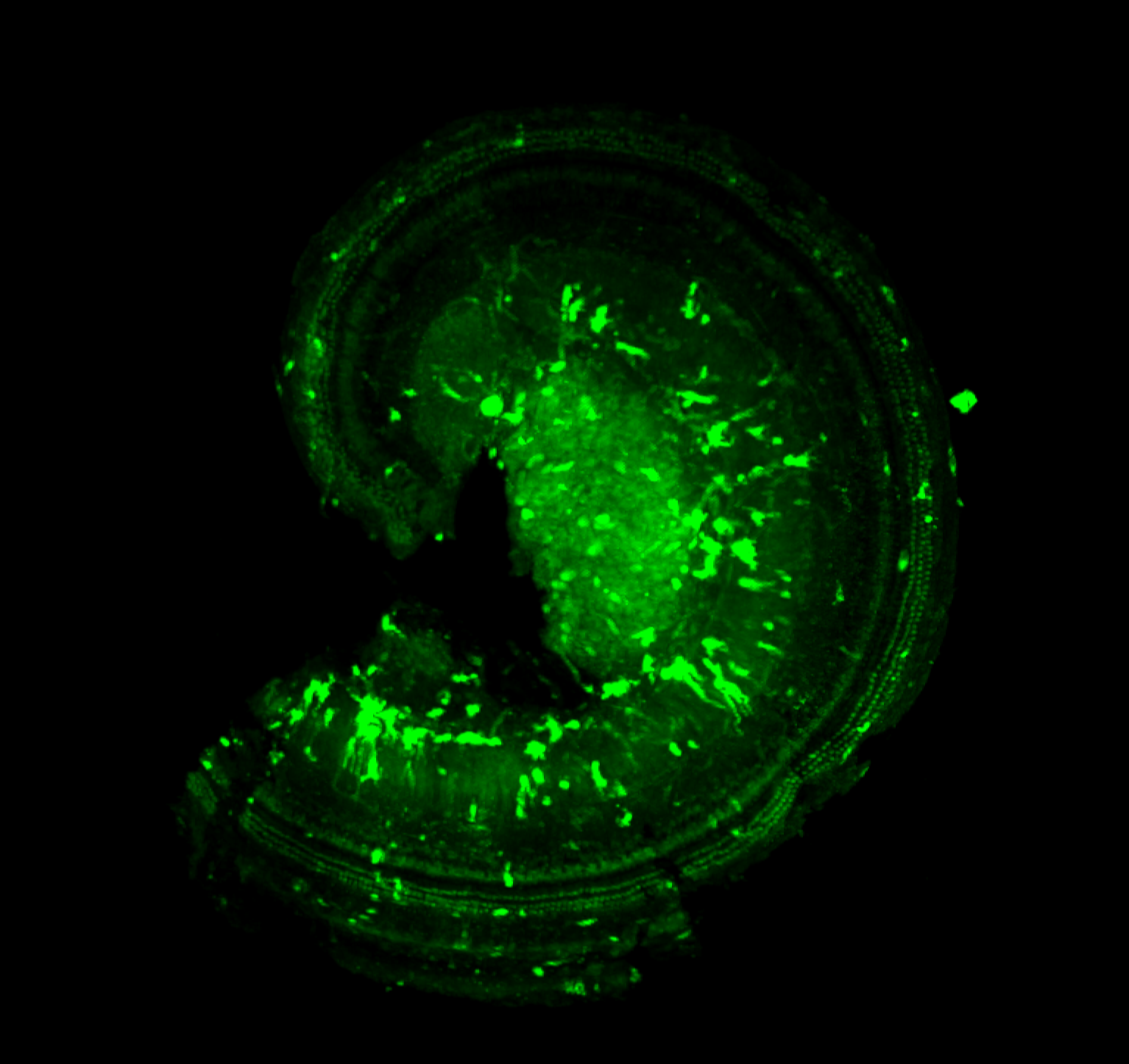

Supplement: Supplementary file 9 — Source data Fig. 6 [file 44321_2025_275_MOESM9_ESM.zip › Manuscript_EMM-2025-21431_SourceDataForFigure6/6C/ssAAV GFP.tif]

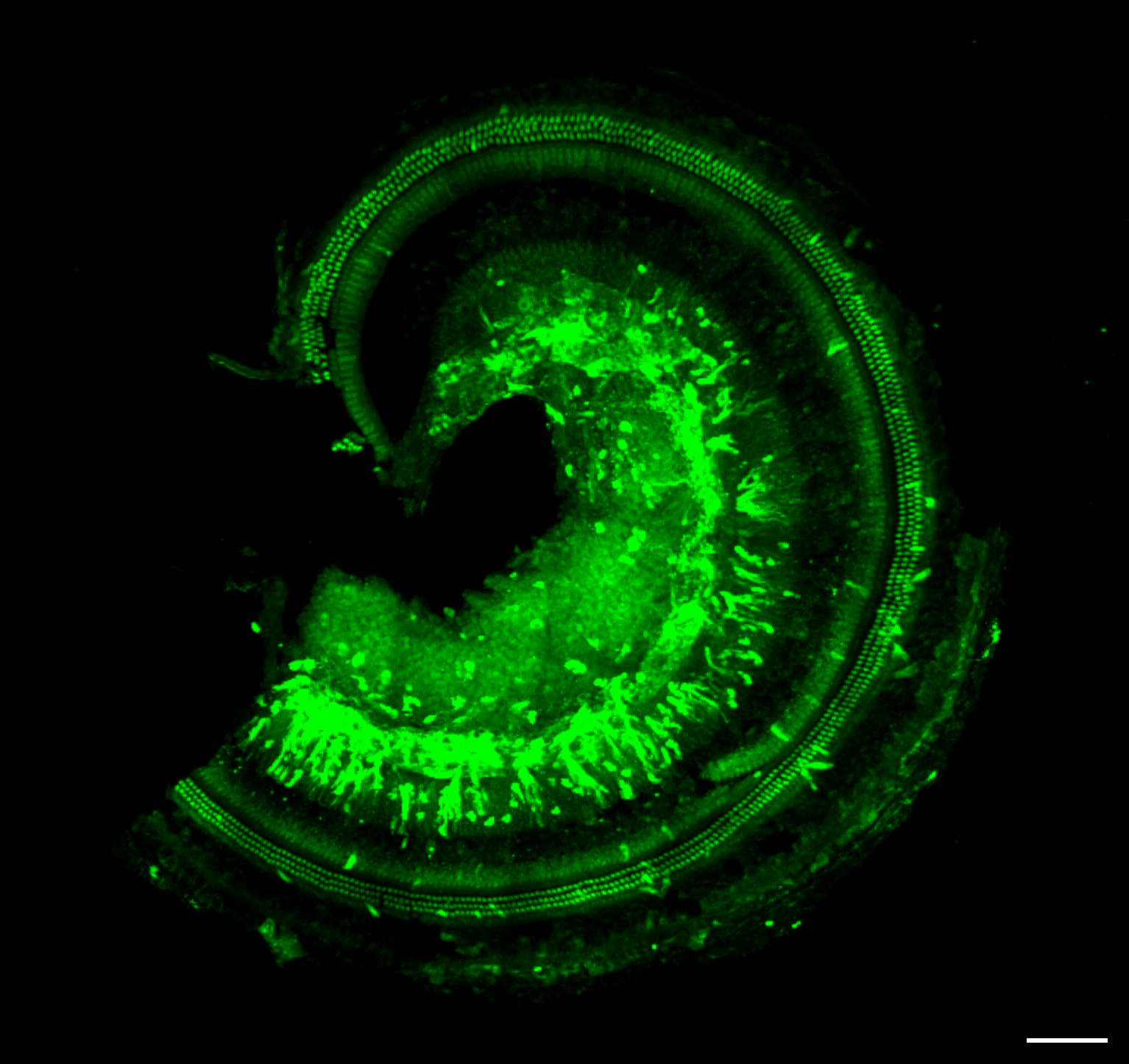

Supplement: Supplementary file 9 — Source data Fig. 6 [file 44321_2025_275_MOESM9_ESM.zip › Manuscript_EMM-2025-21431_SourceDataForFigure6/6C/scAAV GFP_100 microm scale.tif]

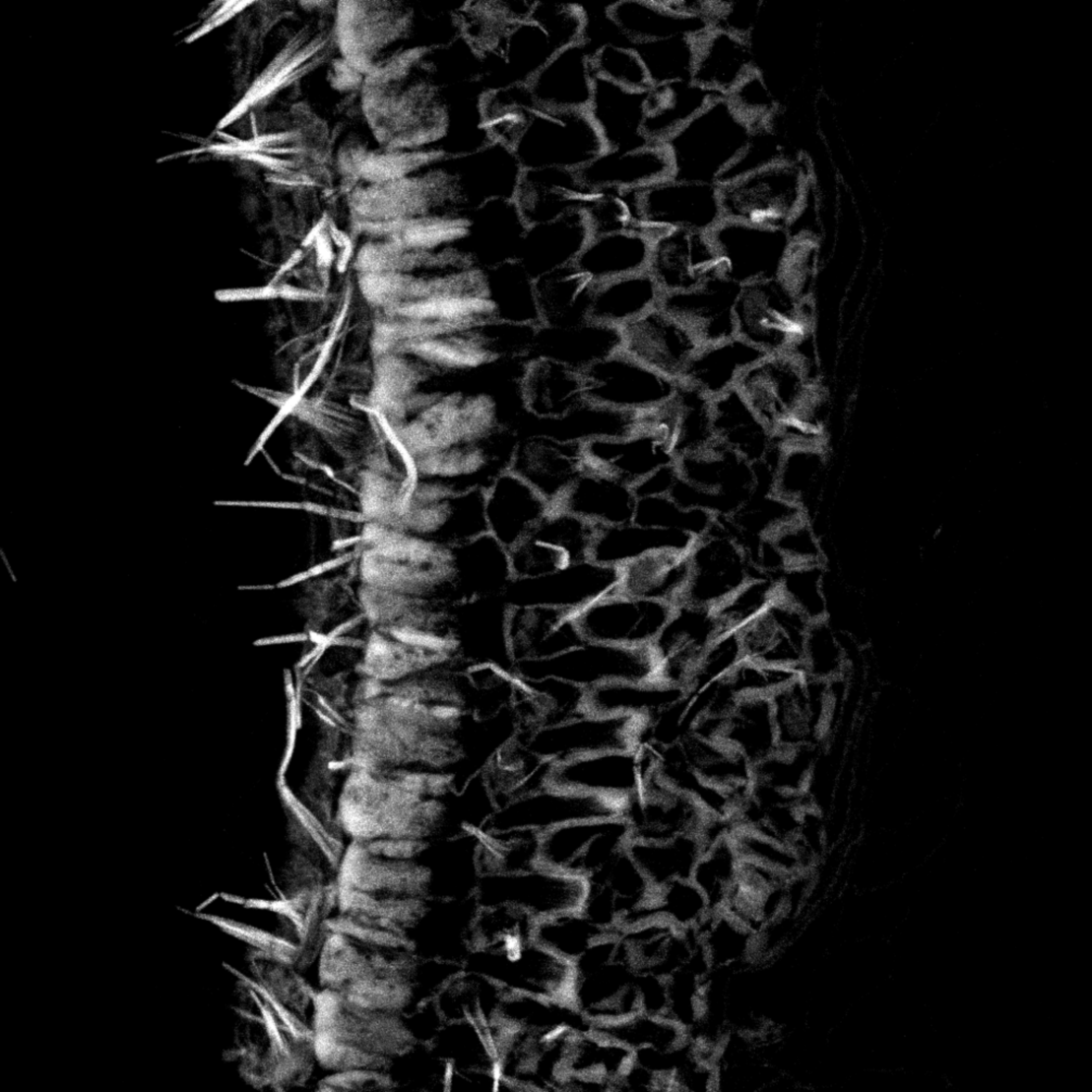

Supplement: Supplementary file 10 — Source data Fig. 7 [file 44321_2025_275_MOESM10_ESM.zip › Manuscript_EMM-2025-21431_SourceDataForFigure7/7C/Hom.tif]

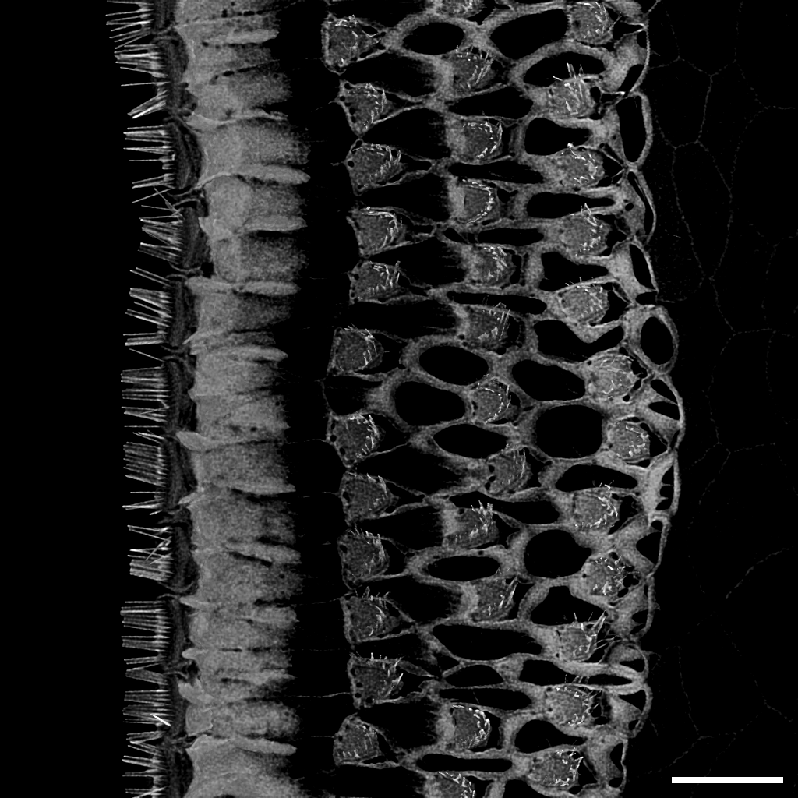

Supplement: Supplementary file 10 — Source data Fig. 7 [file 44321_2025_275_MOESM10_ESM.zip › Manuscript_EMM-2025-21431_SourceDataForFigure7/7C/Hom+ scAAV.tif]

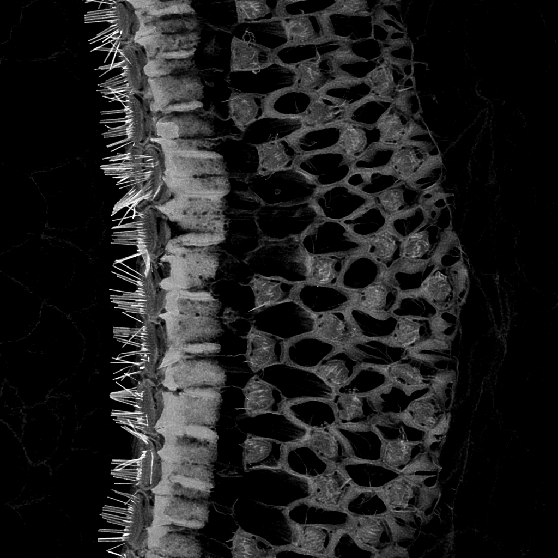

Supplement: Supplementary file 10 — Source data Fig. 7 [file 44321_2025_275_MOESM10_ESM.zip › Manuscript_EMM-2025-21431_SourceDataForFigure7/7C/Het.tif]

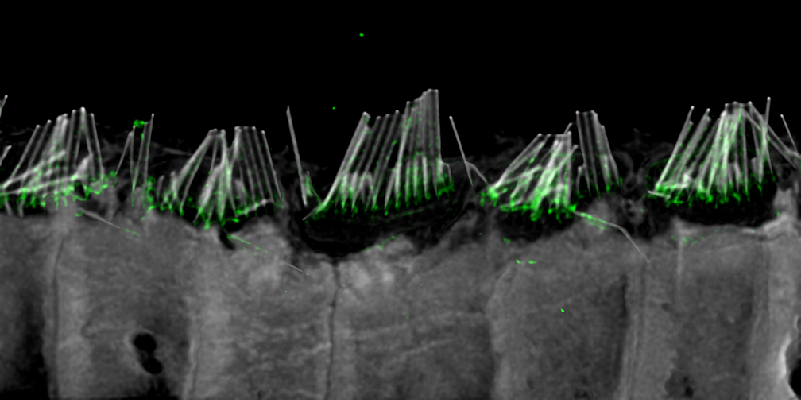

Supplement: Supplementary file 10 — Source data Fig. 7 [file 44321_2025_275_MOESM10_ESM.zip › Manuscript_EMM-2025-21431_SourceDataForFigure7/7B/Hom+ scAAV_Flag.tif]

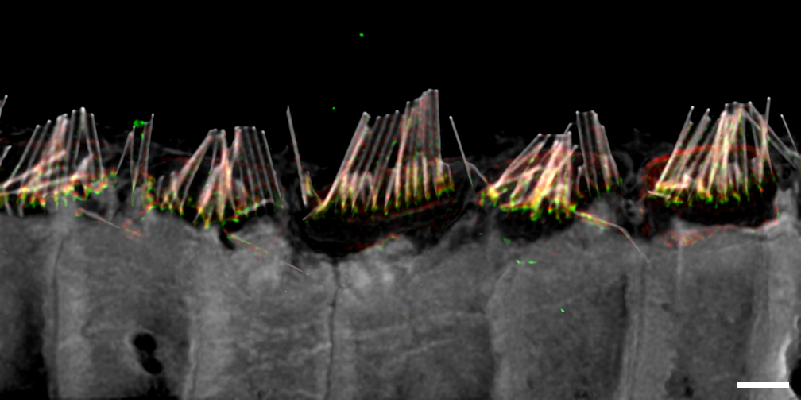

Supplement: Supplementary file 10 — Source data Fig. 7 [file 44321_2025_275_MOESM10_ESM.zip › Manuscript_EMM-2025-21431_SourceDataForFigure7/7B/Hom+ scAAV_merge.tif]

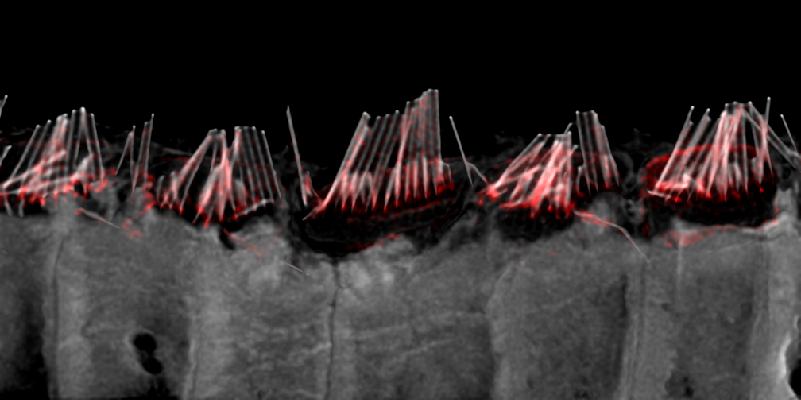

Supplement: Supplementary file 10 — Source data Fig. 7 [file 44321_2025_275_MOESM10_ESM.zip › Manuscript_EMM-2025-21431_SourceDataForFigure7/7B/Hom+ scAAV_Clic5.tif]

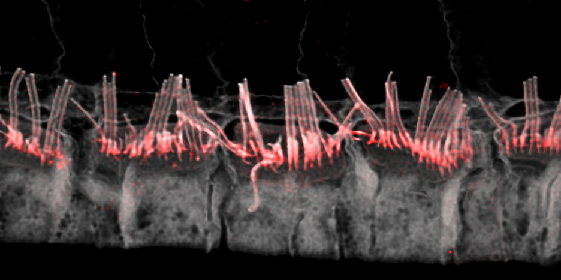

Supplement: Supplementary file 10 — Source data Fig. 7 [file 44321_2025_275_MOESM10_ESM.zip › Manuscript_EMM-2025-21431_SourceDataForFigure7/7B/Het_Clic5.tif]

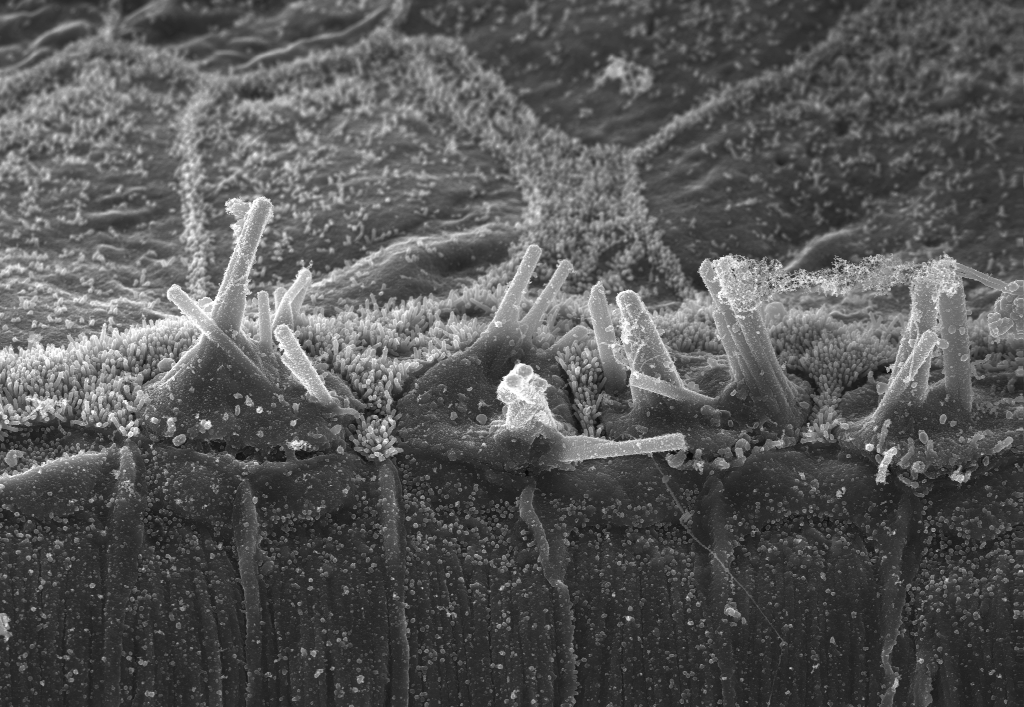

Supplement: Supplementary file 10 — Source data Fig. 7 [file 44321_2025_275_MOESM10_ESM.zip › Manuscript_EMM-2025-21431_SourceDataForFigure7/7G/IHC_Hom.tif]

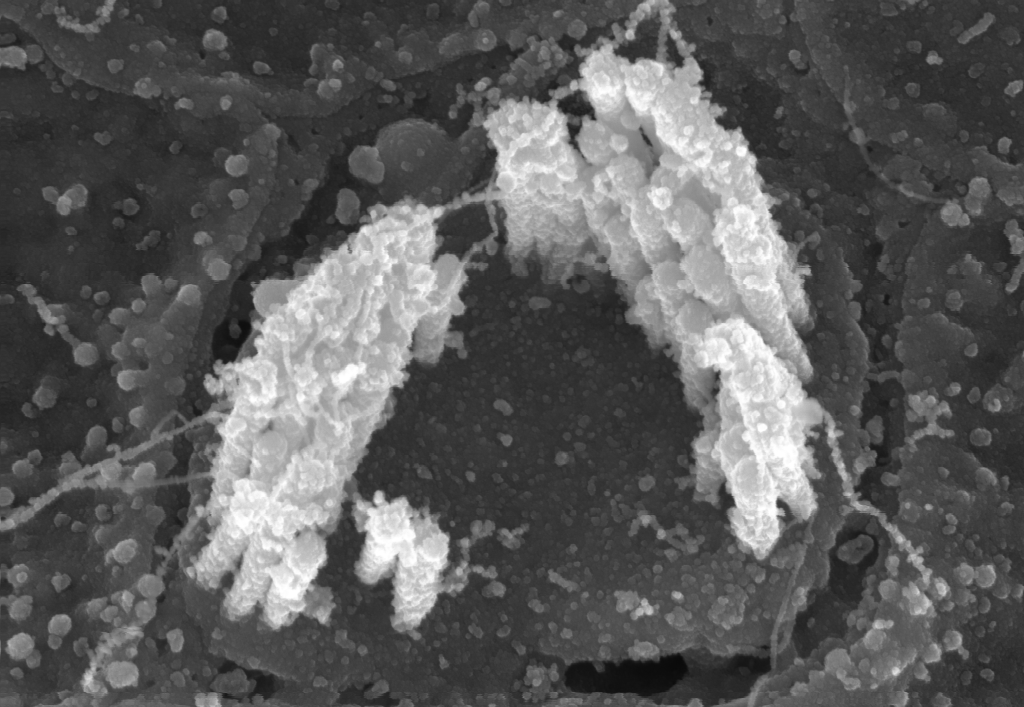

Supplement: Supplementary file 10 — Source data Fig. 7 [file 44321_2025_275_MOESM10_ESM.zip › Manuscript_EMM-2025-21431_SourceDataForFigure7/7G/OHC_Hom.tif]

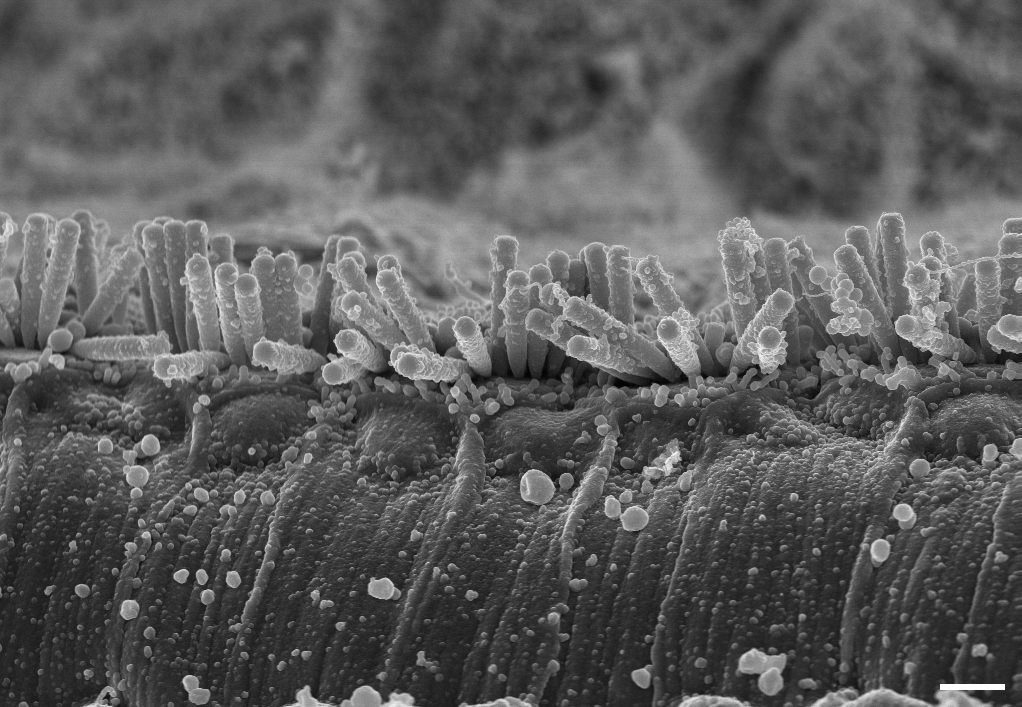

Supplement: Supplementary file 10 — Source data Fig. 7 [file 44321_2025_275_MOESM10_ESM.zip › Manuscript_EMM-2025-21431_SourceDataForFigure7/7G/IHC_Hom+ scAAV.tif]

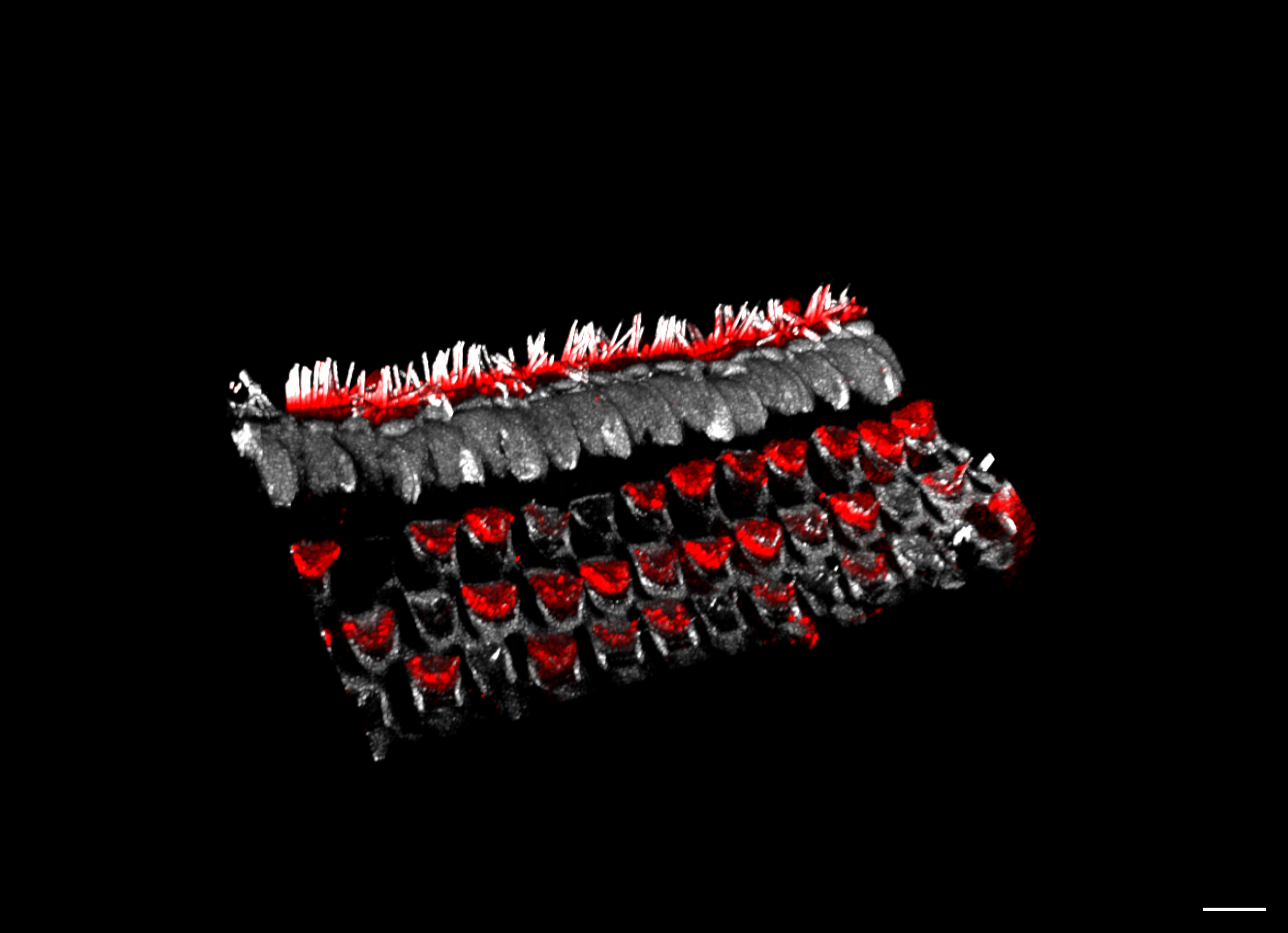

Supplement: Supplementary file 10 — Source data Fig. 7 [file 44321_2025_275_MOESM10_ESM.zip › Manuscript_EMM-2025-21431_SourceDataForFigure7/7A/Hom + scAAV.tif]

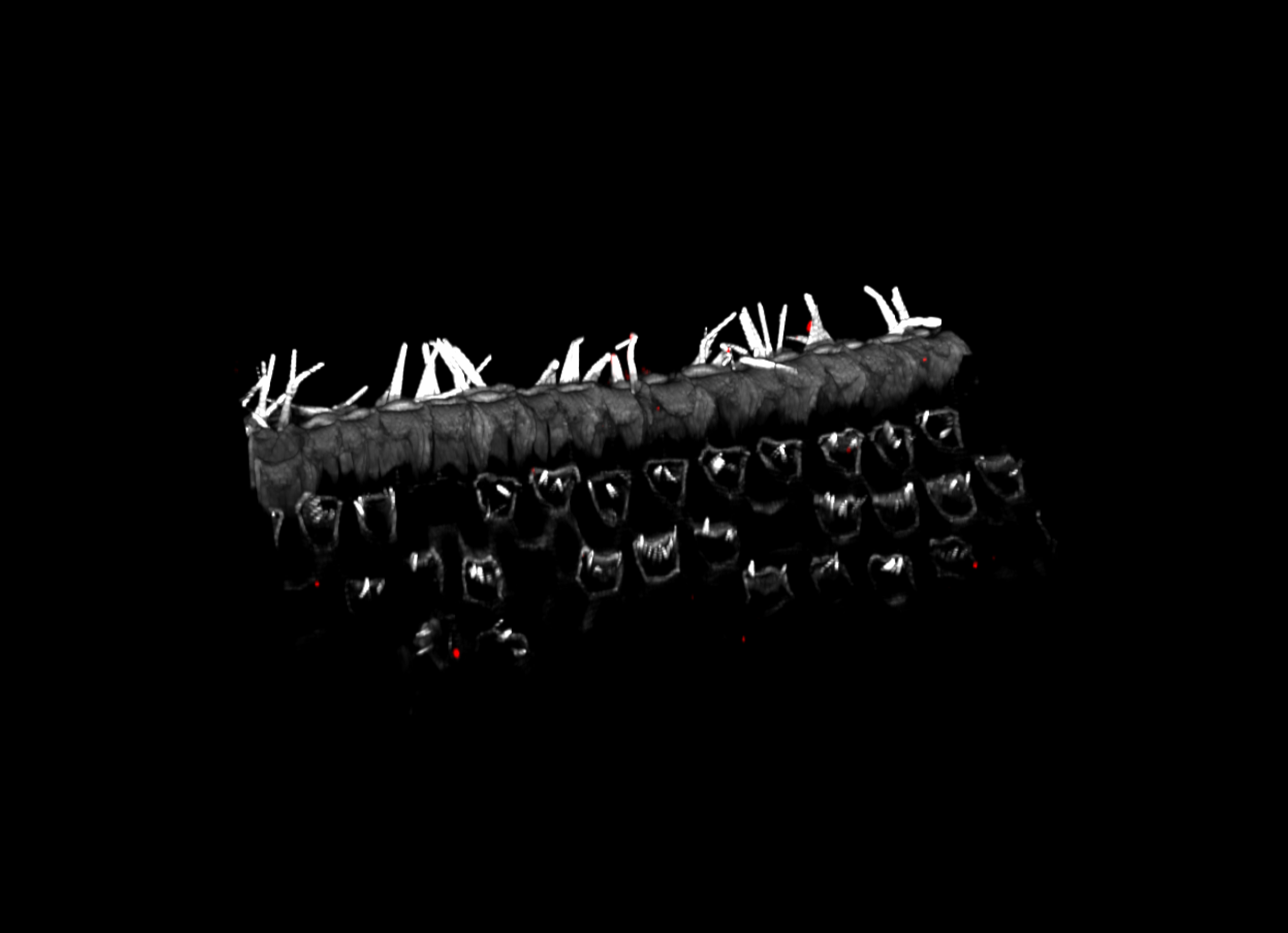

Supplement: Supplementary file 10 — Source data Fig. 7 [file 44321_2025_275_MOESM10_ESM.zip › Manuscript_EMM-2025-21431_SourceDataForFigure7/7A/Hom.tif]

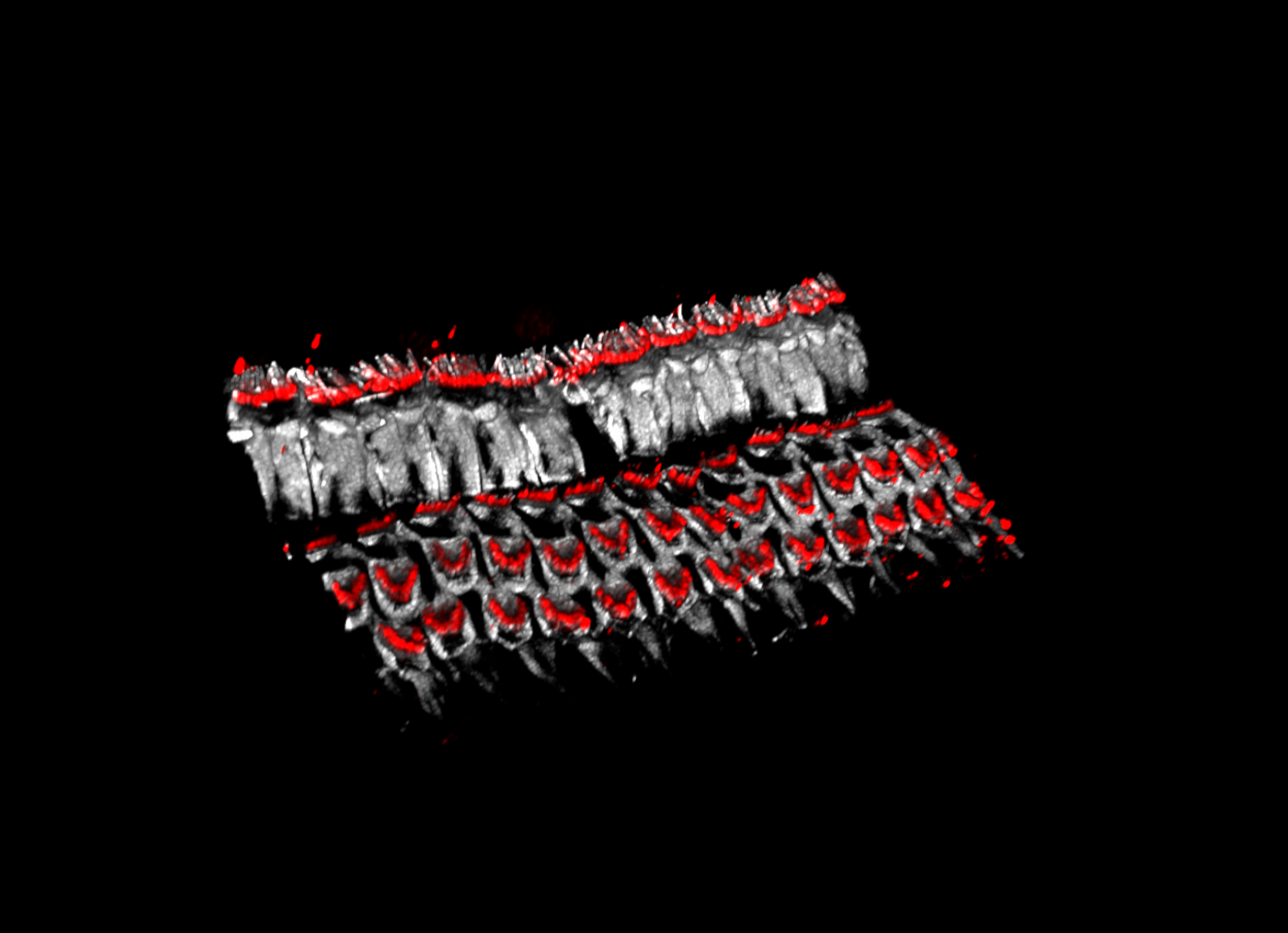

Supplement: Supplementary file 10 — Source data Fig. 7 [file 44321_2025_275_MOESM10_ESM.zip › Manuscript_EMM-2025-21431_SourceDataForFigure7/7A/Het.tif]

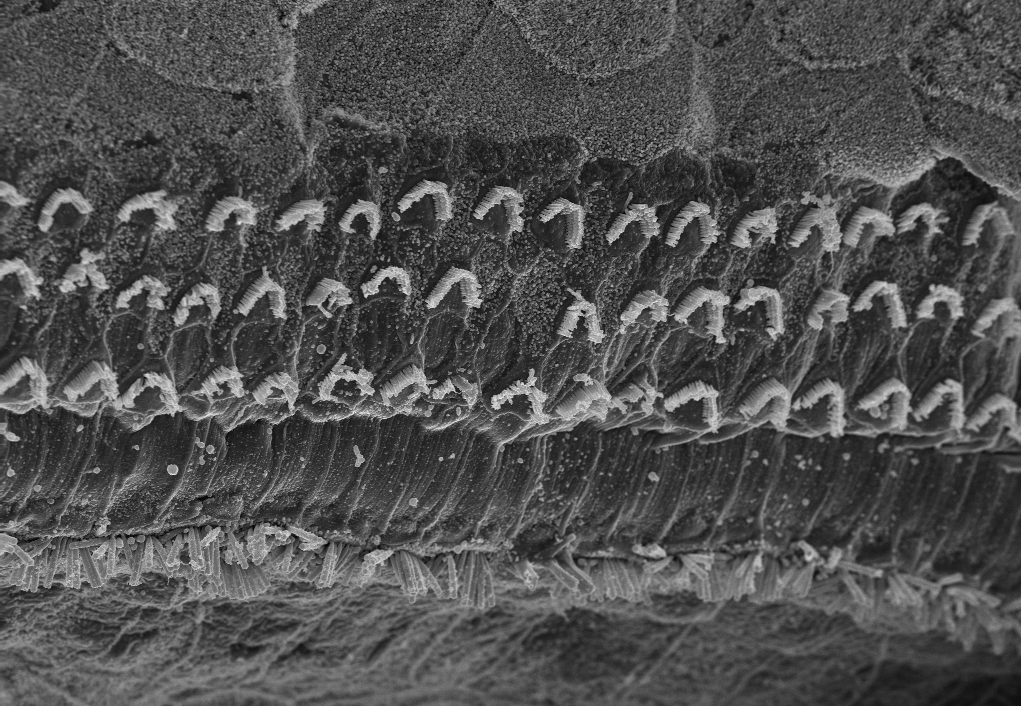

Supplement: Supplementary file 10 — Source data Fig. 7 [file 44321_2025_275_MOESM10_ESM.zip › Manuscript_EMM-2025-21431_SourceDataForFigure7/7H/Apex_Hom+ scAAV.tif]

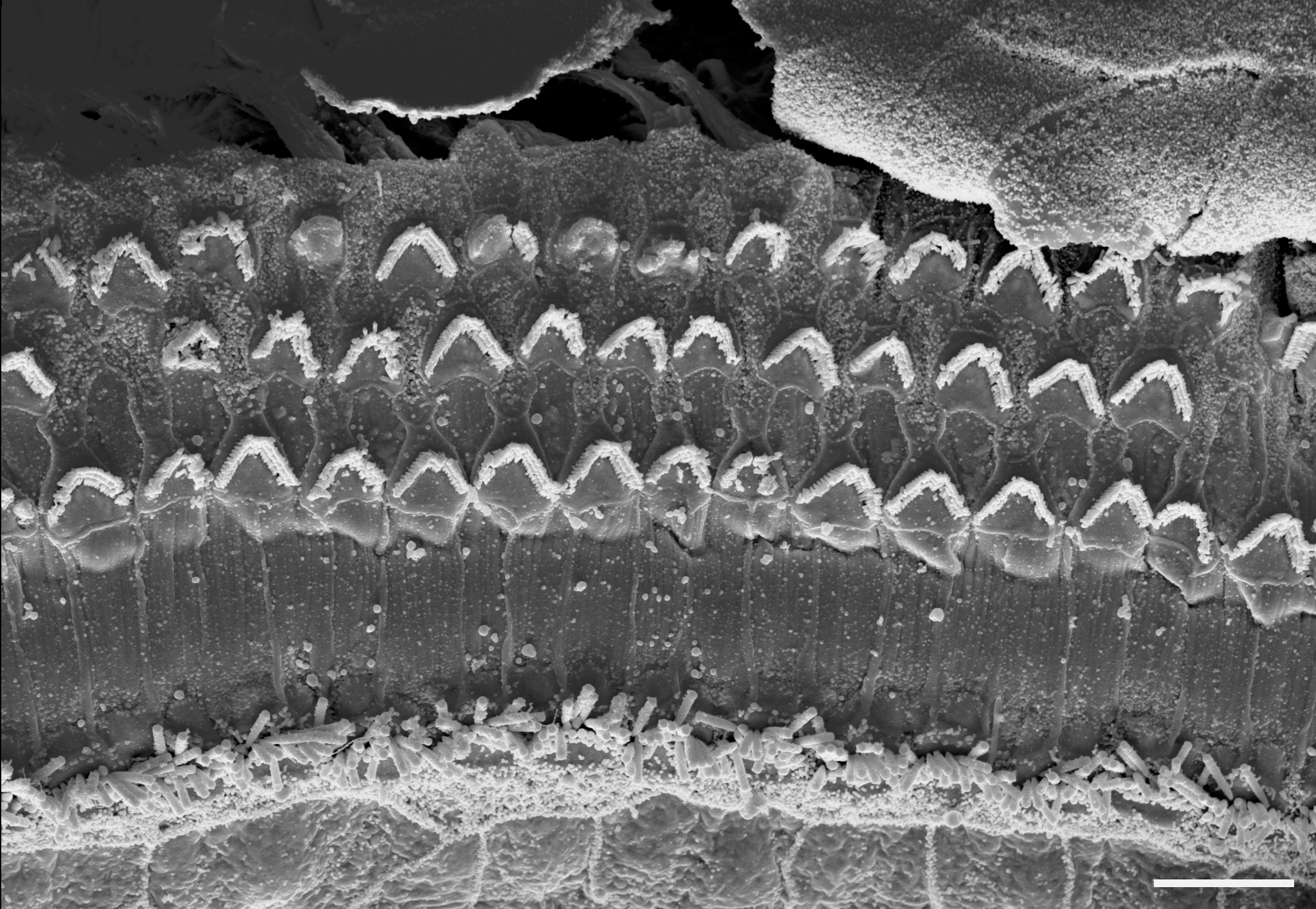

Supplement: Supplementary file 10 — Source data Fig. 7 [file 44321_2025_275_MOESM10_ESM.zip › Manuscript_EMM-2025-21431_SourceDataForFigure7/7H/Base_Hom+ scAAV.tif]

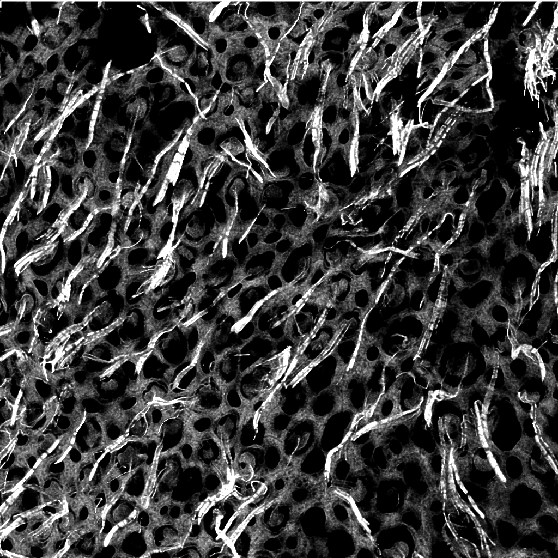

Supplement: Supplementary file 11 — Source data Fig. 8 [file 44321_2025_275_MOESM11_ESM.zip › Manuscript_EMM-2025-21431_SourceDataForFigure8/8A/Hom.tif]

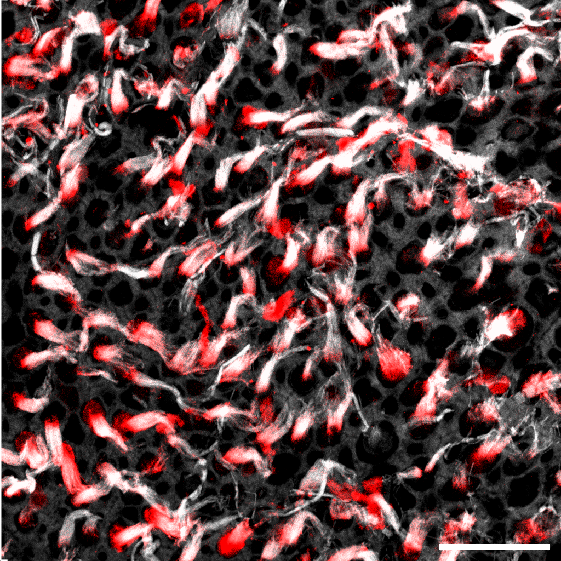

Supplement: Supplementary file 11 — Source data Fig. 8 [file 44321_2025_275_MOESM11_ESM.zip › Manuscript_EMM-2025-21431_SourceDataForFigure8/8A/Hom+ scAAV.tif]

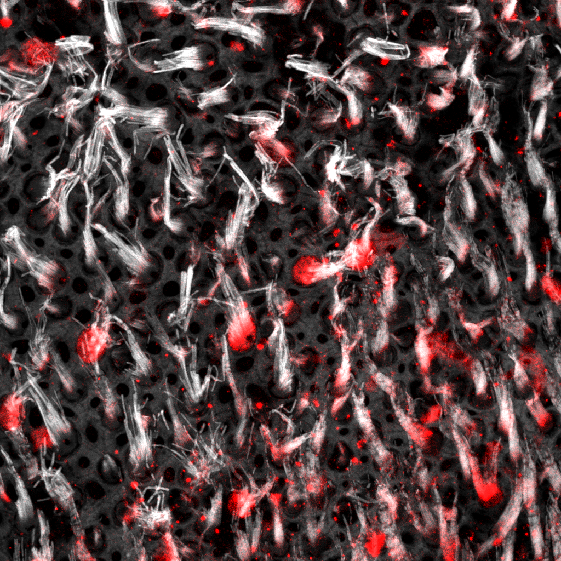

Supplement: Supplementary file 11 — Source data Fig. 8 [file 44321_2025_275_MOESM11_ESM.zip › Manuscript_EMM-2025-21431_SourceDataForFigure8/8A/Het.tif]

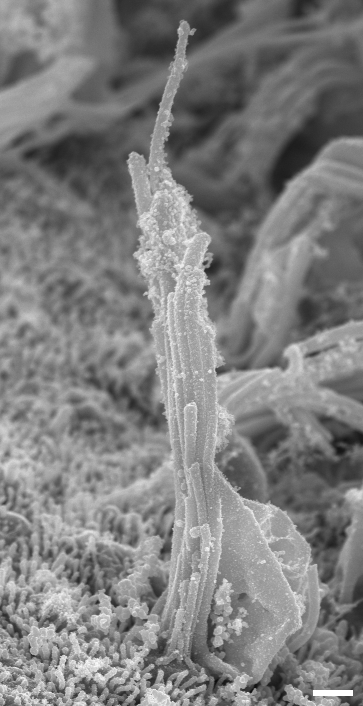

Supplement: Supplementary file 11 — Source data Fig. 8 [file 44321_2025_275_MOESM11_ESM.zip › Manuscript_EMM-2025-21431_SourceDataForFigure8/8D/Hom.tif]

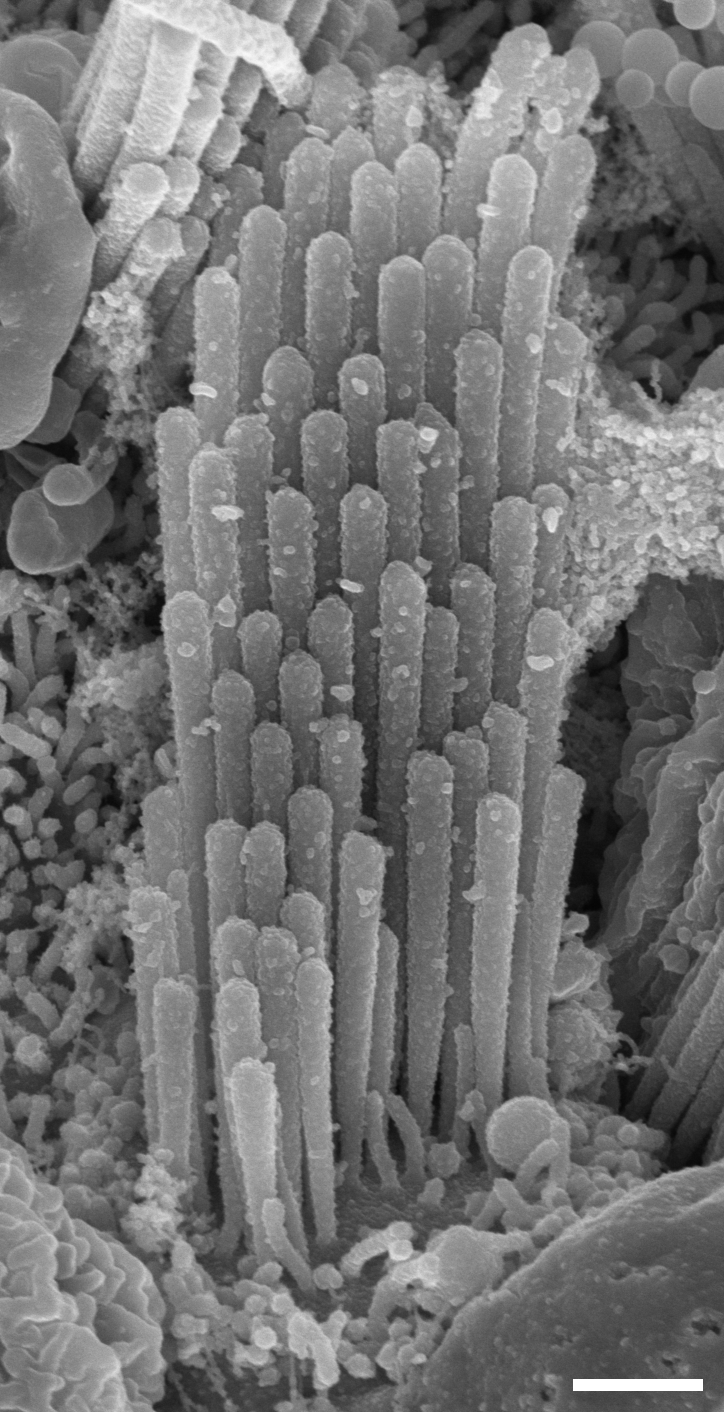

Supplement: Supplementary file 11 — Source data Fig. 8 [file 44321_2025_275_MOESM11_ESM.zip › Manuscript_EMM-2025-21431_SourceDataForFigure8/8D/Hom+ scAAV.tif]

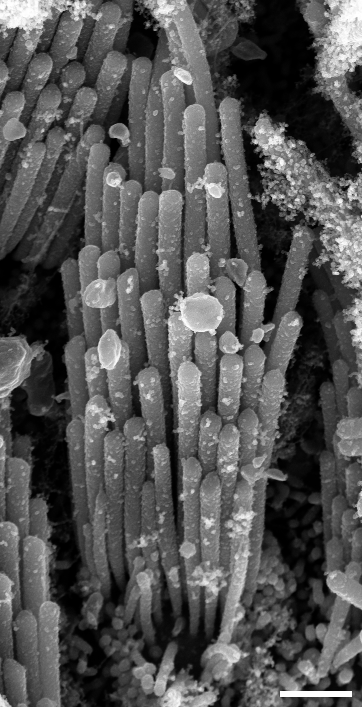

Supplement: Supplementary file 11 — Source data Fig. 8 [file 44321_2025_275_MOESM11_ESM.zip › Manuscript_EMM-2025-21431_SourceDataForFigure8/8D/Het.tif]

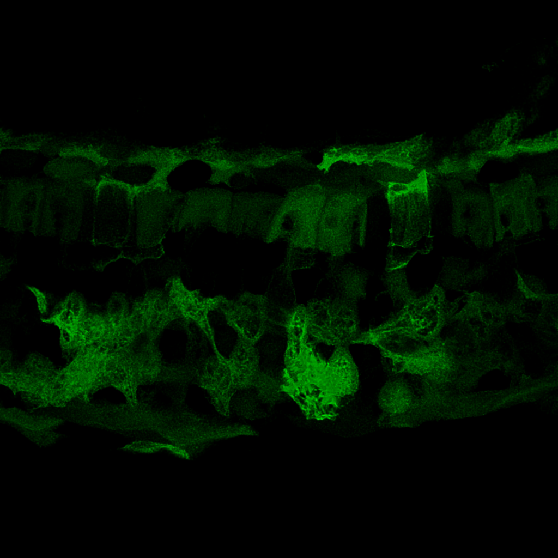

Supplement: Supplementary file 12 — Figure EV1 Source Data [file 44321_2025_275_MOESM12_ESM.zip › Manuscript_EMM-2025-21431_SourceDataForFigureEV1/EV1A/scAAV.GFP_gfp.tif]

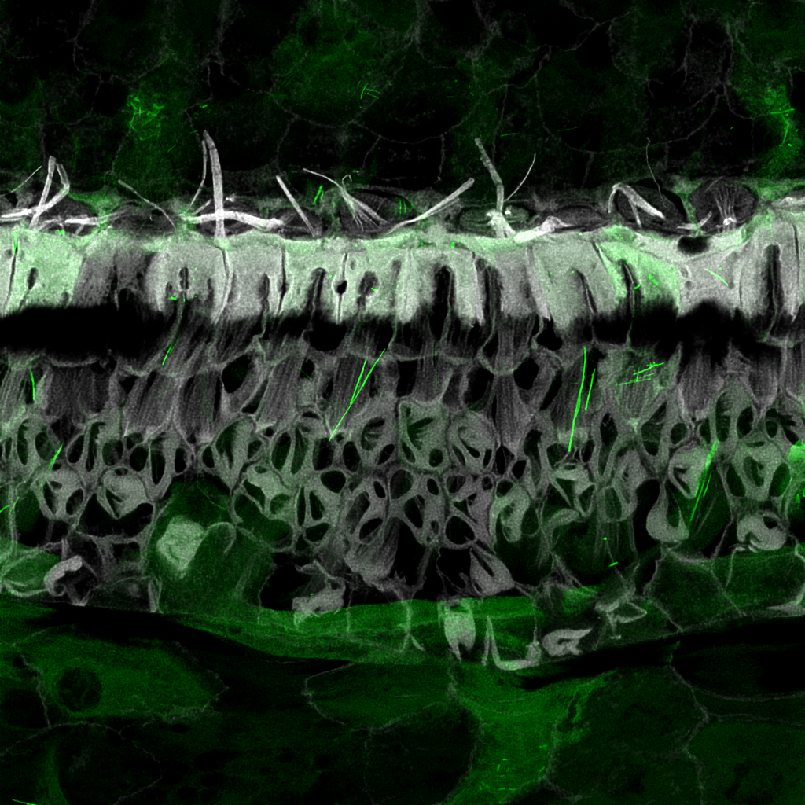

Supplement: Supplementary file 12 — Figure EV1 Source Data [file 44321_2025_275_MOESM12_ESM.zip › Manuscript_EMM-2025-21431_SourceDataForFigureEV1/EV1A/ssAAV.GFP_merge.tif]

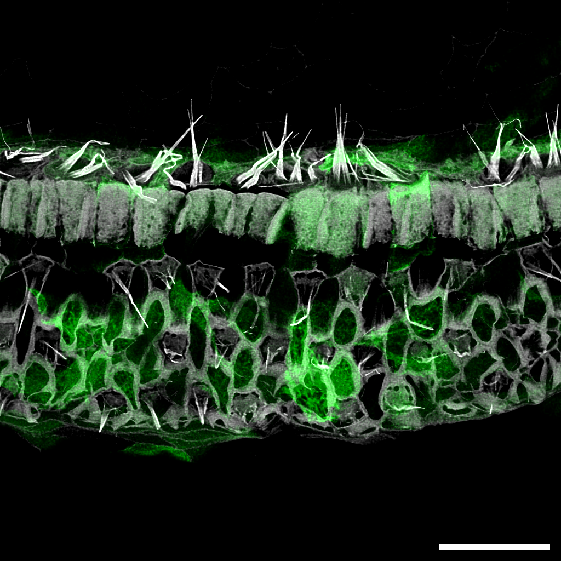

Supplement: Supplementary file 12 — Figure EV1 Source Data [file 44321_2025_275_MOESM12_ESM.zip › Manuscript_EMM-2025-21431_SourceDataForFigureEV1/EV1A/scAAV.GFP_merge.tif]

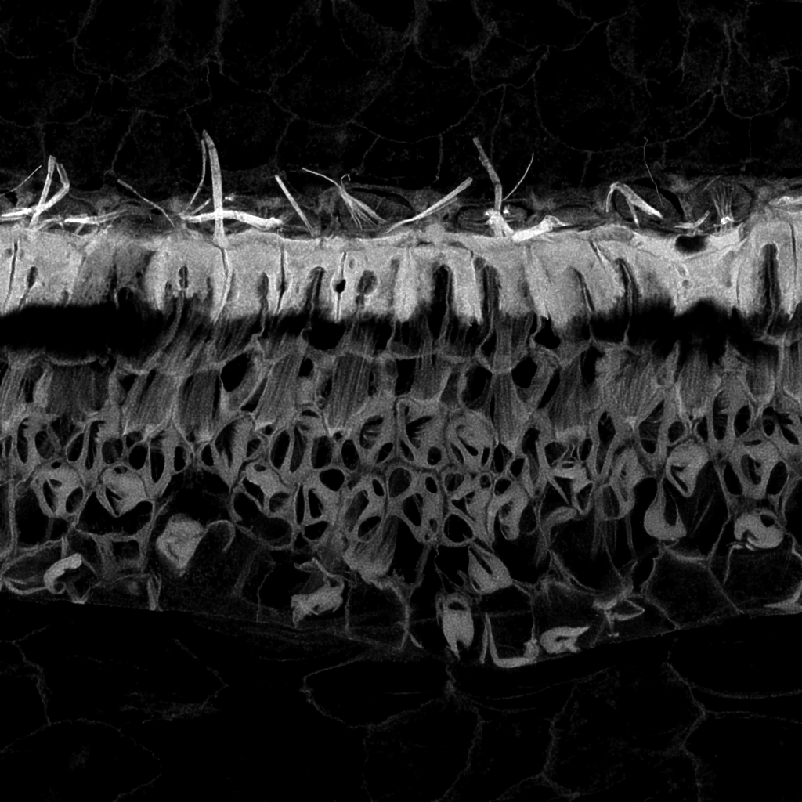

Supplement: Supplementary file 12 — Figure EV1 Source Data [file 44321_2025_275_MOESM12_ESM.zip › Manuscript_EMM-2025-21431_SourceDataForFigureEV1/EV1A/ssAAV.GFP_actin.tif]

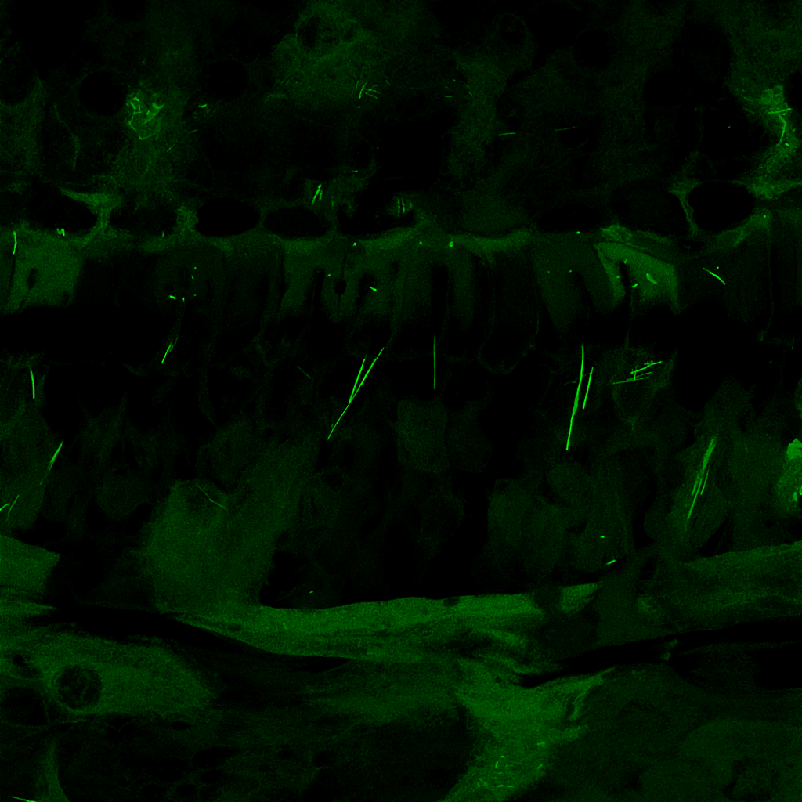

Supplement: Supplementary file 12 — Figure EV1 Source Data [file 44321_2025_275_MOESM12_ESM.zip › Manuscript_EMM-2025-21431_SourceDataForFigureEV1/EV1A/ssAAV.GFP_gfp.tif]

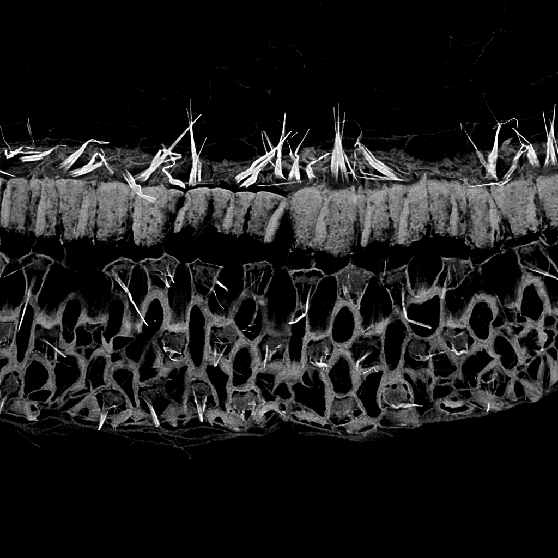

Supplement: Supplementary file 12 — Figure EV1 Source Data [file 44321_2025_275_MOESM12_ESM.zip › Manuscript_EMM-2025-21431_SourceDataForFigureEV1/EV1A/scAAV.GFP_actin.tif]

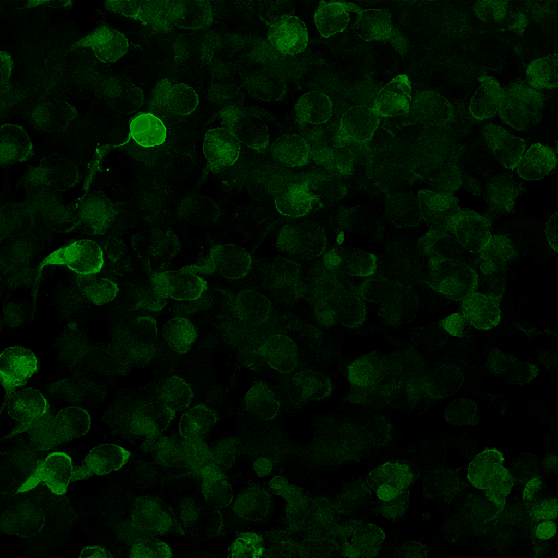

Supplement: Supplementary file 14 — Figure EV3 Source Data [file 44321_2025_275_MOESM14_ESM.zip › Manuscript_EMM-2025-21431_SourceDataForFigureEV3/EV3A/scAAV.GFP_gfp.tif]

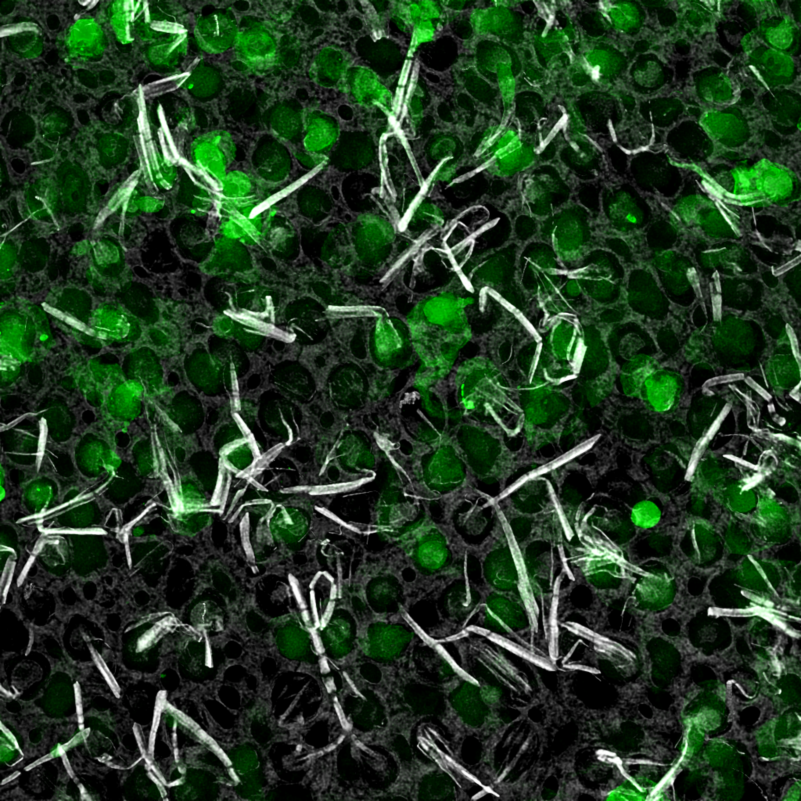

Supplement: Supplementary file 14 — Figure EV3 Source Data [file 44321_2025_275_MOESM14_ESM.zip › Manuscript_EMM-2025-21431_SourceDataForFigureEV3/EV3A/ssAAV.GFP_merge.tif]

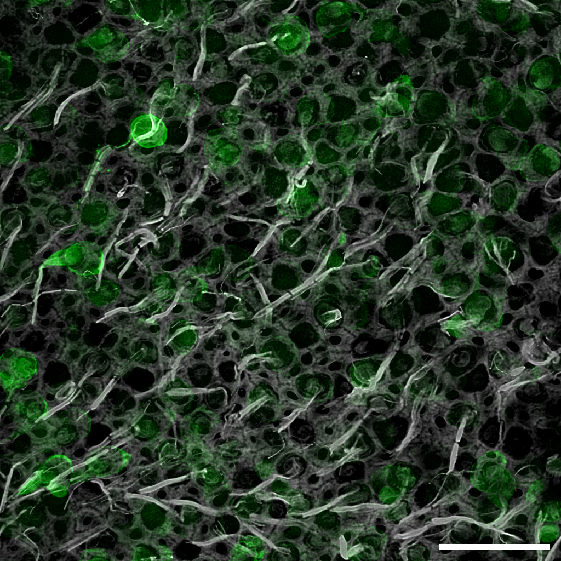

Supplement: Supplementary file 14 — Figure EV3 Source Data [file 44321_2025_275_MOESM14_ESM.zip › Manuscript_EMM-2025-21431_SourceDataForFigureEV3/EV3A/scAAV.GFP_merge.tif]

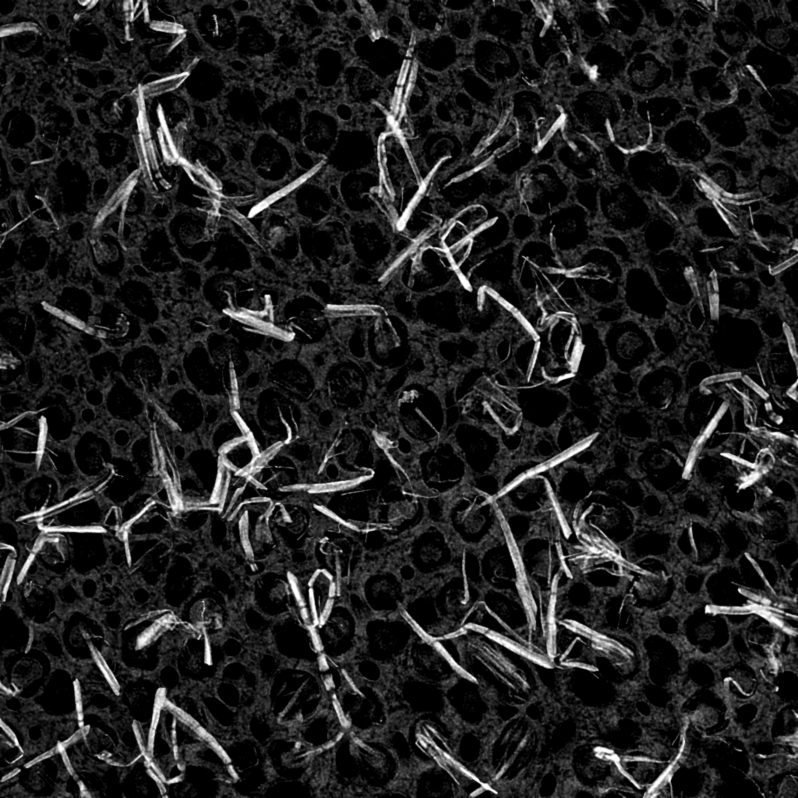

Supplement: Supplementary file 14 — Figure EV3 Source Data [file 44321_2025_275_MOESM14_ESM.zip › Manuscript_EMM-2025-21431_SourceDataForFigureEV3/EV3A/ssAAV.GFP_actin.tif]

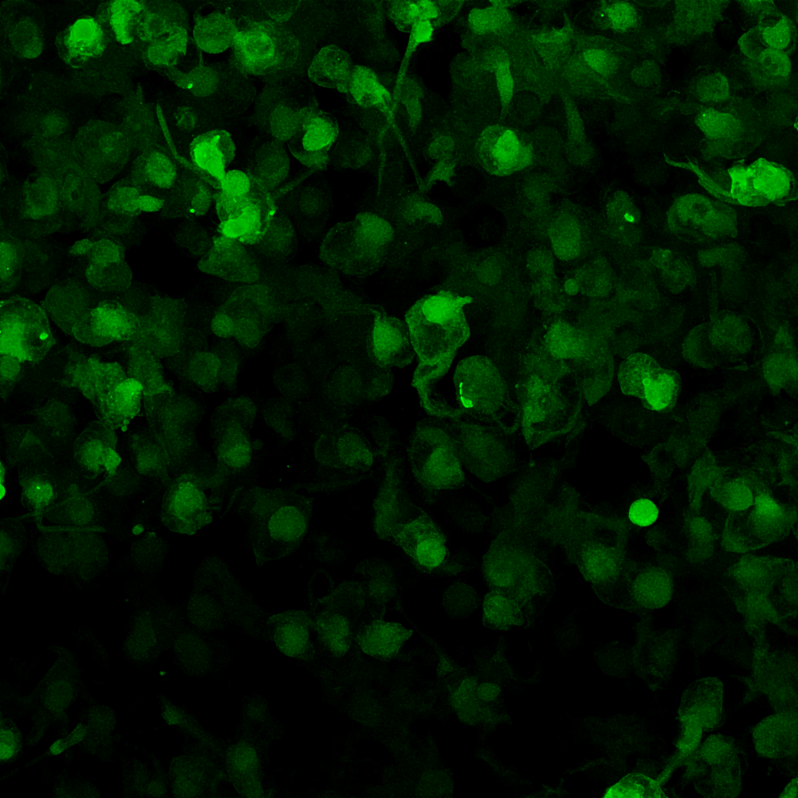

Supplement: Supplementary file 14 — Figure EV3 Source Data [file 44321_2025_275_MOESM14_ESM.zip › Manuscript_EMM-2025-21431_SourceDataForFigureEV3/EV3A/ssAAV.GFP_gfp.tif]

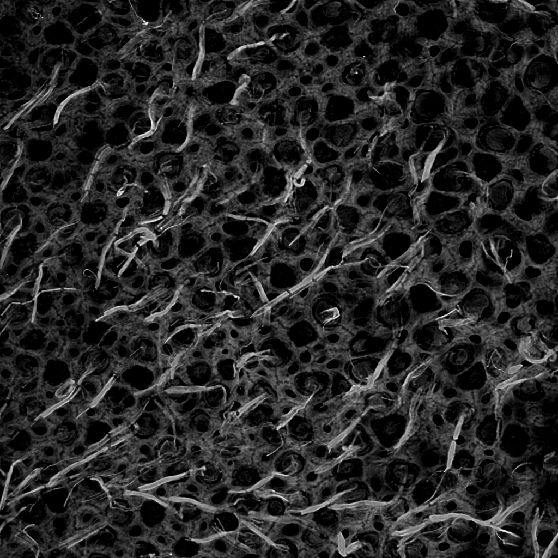

Supplement: Supplementary file 14 — Figure EV3 Source Data [file 44321_2025_275_MOESM14_ESM.zip › Manuscript_EMM-2025-21431_SourceDataForFigureEV3/EV3A/scAAV.GFP_actin.tif]
