# Supplementary material for: AAV gene therapy rescues hearing and balance in a model of CLIC5 deafness
Source: EMBO Mol Med. 2025 Aug 26;17(9):2233–57. doi: 10.1038/s44321-025-00275-7 (PMC12423326; doi:10.1038/s44321-025-00275-7)
Supplement: Supplementary file 17 — Expanded View Figures [file 44321_2025_275_MOESM17_ESM.pdf]

Expanded View Figures

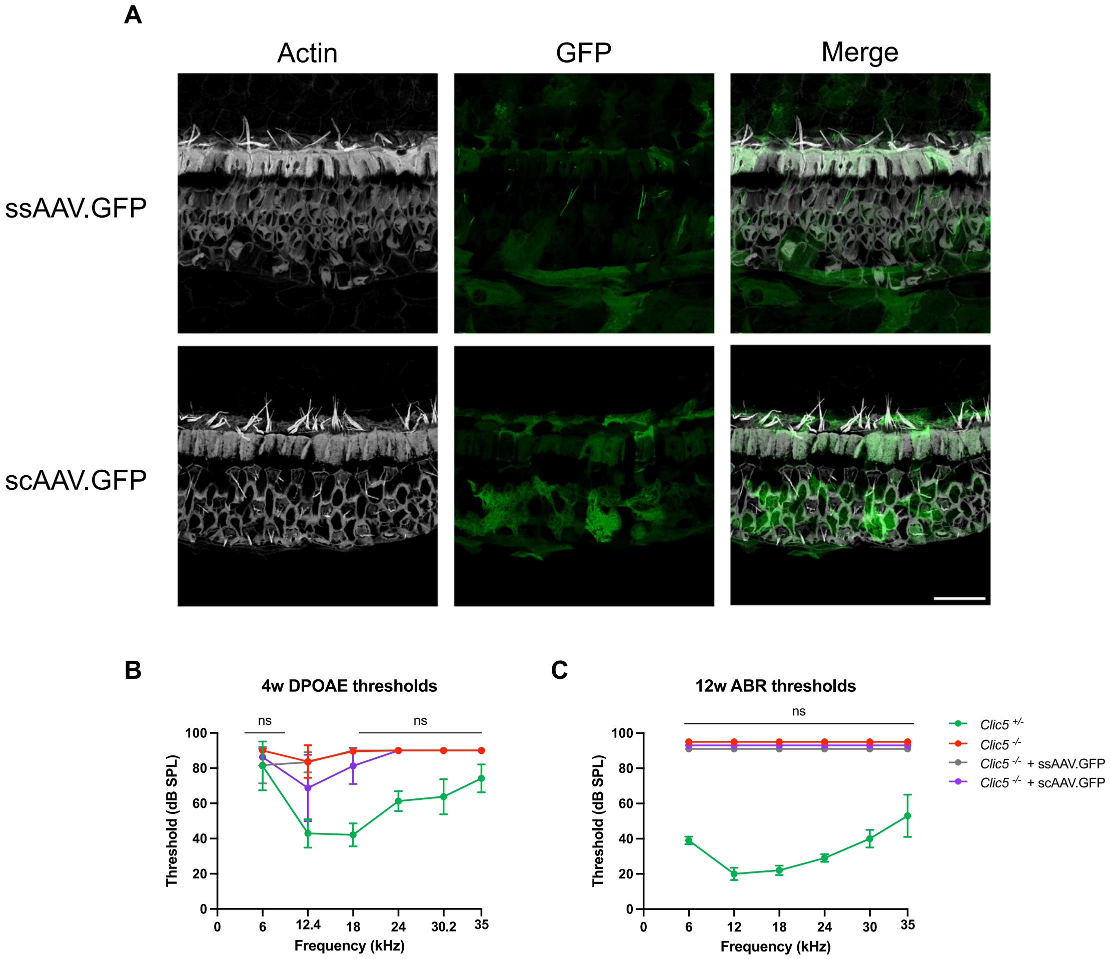

Figure EV1. Single-stranded or self-complementary AAV.GFP does not rescue the morphology or function of the auditory system.

(**A**) Apical region of the cochlea of 12 weeks *Clic5*$^{-/-}$ injected with ssAAV.GFP or scAAV.GFP. (**B**) DPOAE thresholds at 4 weeks of *Clic5*$^{+/-}$ ($n = 12$), *Clic5*$^{-/-}$ ($n = 12$), *Clic5*$^{-/-}$ injected with ssAAV.GFP ($n = 3$) or scAAV.GFP. ($n = 4$). (**C**) ABR thresholds at 12 weeks of *Clic5*$^{+/-}$ ($n = 5$), *Clic5*$^{-/-}$ ($n = 10$), *Clic5*$^{-/-}$ injected with ssAAV.GFP ($n = 3$) or scAAV.GFP. ($n = 4$). Data information: The statistics test was two-way ANOVA with Holm-Sidak correction for multiple comparisons. Plots show mean ± SD. ns = not significant. Scale bar = 10 µm. Source data are available online for this figure.

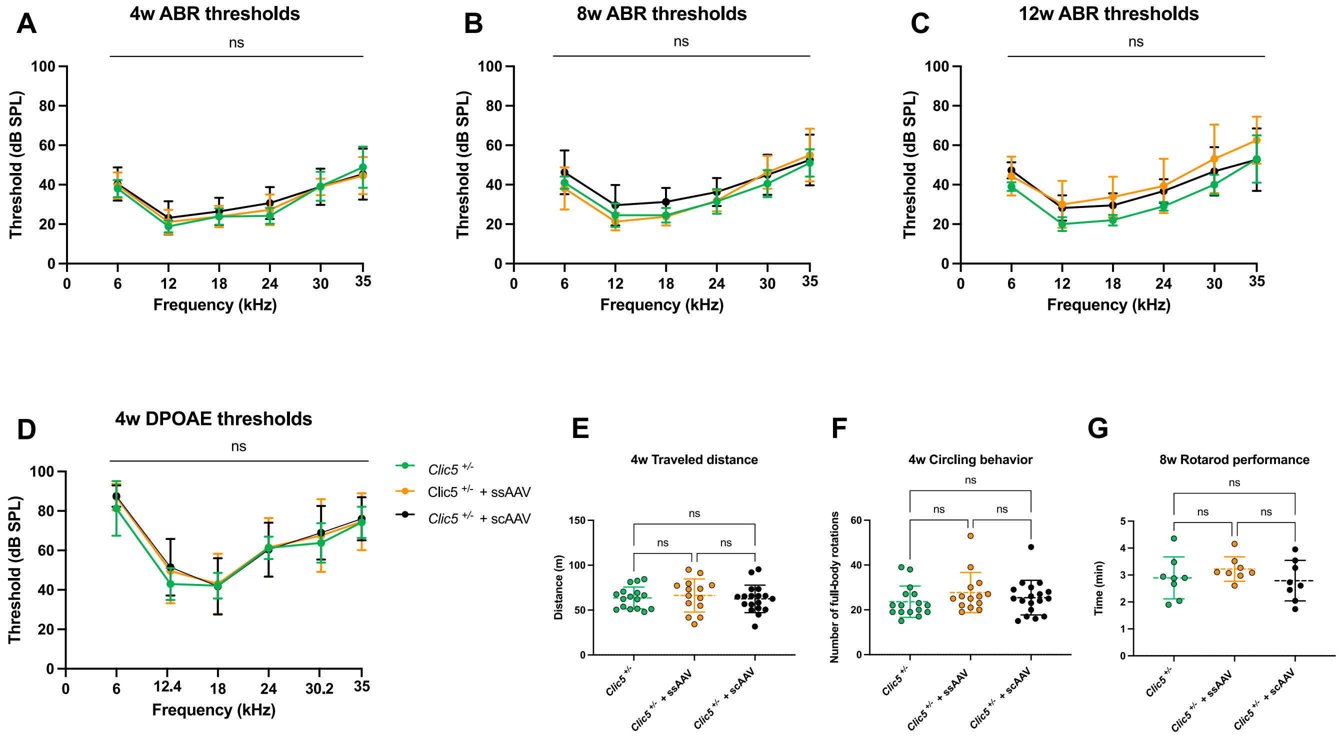

Figure EV2. Single-stranded or self-complementary AAV.*Clic5* does not affect hearing and vestibular function of control mice.

(**A**) ABR thresholds at 4 weeks of *Clic5*$^{+/-}$ ($n = 13$), *Clic5*$^{+/-}$ injected with ssAAV.*Clic5* ($n = 13$) or with scAAV.*Clic5* ($n = 14$). (**B**) ABR thresholds at 8 weeks of *Clic5*$^{+/-}$ ($n = 10$), *Clic5*$^{+/-}$ injected with ssAAV.*Clic5* ($n = 8$) or with scAAV.*Clic5* ($n = 12$). (**C**) ABR thresholds at 12 weeks of *Clic5*$^{+/-}$ ($n = 5$), *Clic5*$^{+/-}$ injected with ssAAV.*Clic5* ($n = 8$) or with scAAV.*Clic5* ($n = 11$). (**D**) DPOAE thresholds at 4 weeks of *Clic5*$^{+/-}$ ($n = 12$), *Clic5*$^{+/-}$ injected with ssAAV.*Clic5* ($n = 10$) or with scAAV.*Clic5* ($n = 14$). (**E**) Distance traveled in the open-field test of *Clic5*$^{+/-}$ ($n = 16$) and *Clic5*$^{+/-}$ injected with ssAAV.*Clic5* ($n = 14$) or scAAV.*Clic5* ($n = 18$), performed at 4 weeks. (**F**) Quantification of circling behavior during the open field test of *Clic5*$^{+/-}$ ($n = 16$) and *Clic5*$^{+/-}$ injected with ssAAV.*Clic5* ($n = 14$) or scAAV.*Clic5* ($n = 18$), performed at 4 weeks. (**G**) Quantification of the average duration on the rotarod of *Clic5*$^{+/-}$ ($n = 8$) and *Clic5*$^{+/-}$ injected with ssAAV.*Clic5* ($n = 8$) or scAAV.*Clic5* ($n = 8$), performed at 8 weeks. Data information: The statistical tests were two-way ANOVA with Holm–Sidak correction for multiple comparisons for (**A–D**) and one-way ANOVA followed by Tukey correction for multiple comparisons for (**E–G**). Plots show mean ± SD. ns = not significant. Source data are available online for this figure.

 © The Author(s)

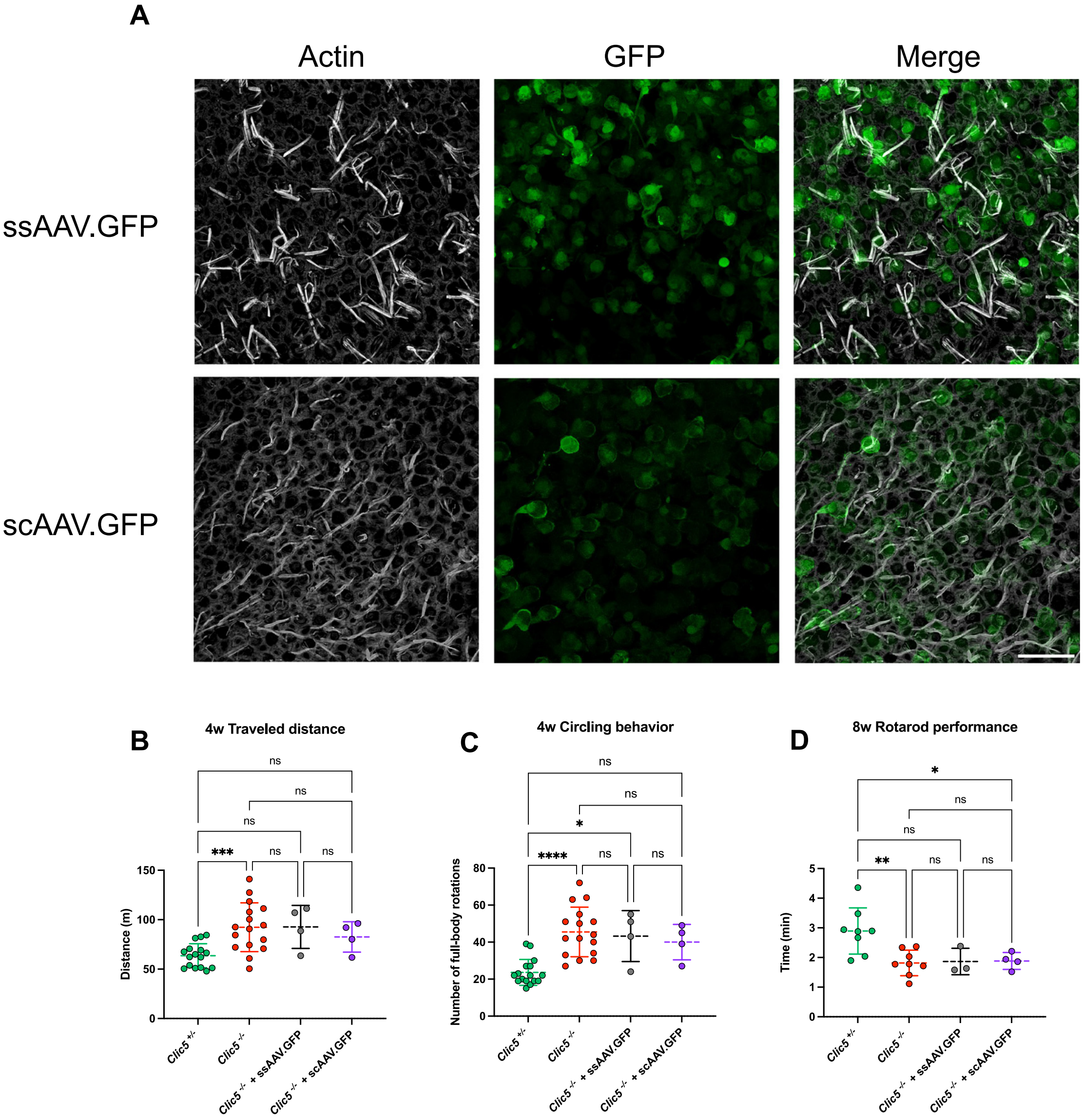

Figure EV3. Single-stranded or self-complementary AAV.GFP does not rescue the morphology or function of the vestibular system.

(**A**) High-magnification image of utricular hair cells of 12 weeks *Clic5*$^{-/-}$ injected with ssAAV.GFP or scAAV.GFP. (**B**) Distance traveled in the open-field test of *Clic5*$^{+/-}$ ($n = 16$), *Clic5*$^{-/-}$ ($n = 16$), *Clic5*$^{-/-}$ injected with ssAAV.GFP ($n = 4$), or scAAV.GFP ($n = 4$), performed at 4 weeks. (**C**) Quantification of circling behavior during the open field test of *Clic5*$^{+/-}$($n = 16$), *Clic5*$^{-/-}$ ($n = 16$), *Clic5*$^{-/-}$ injected with ssAAV.GFP ($n = 4$), or scAAV.GFP ($n = 4$), performed at 4 weeks. (**D**) Quantification of the average duration on the rotarod of *Clic5*$^{+/-}$ ($n = 8$), *Clic5*$^{-/-}$ ($n = 8$), *Clic5*$^{-/-}$ injected with ssAAV.GFP ($n = 3$), or scAAV.GFP ($n = 4$), performed at 8 weeks. Data information: The statistics test was one-way ANOVA followed by Tukey correction for multiple comparisons. Plots show mean ± SD. ns = not significant, *$P < 0.05$, **$P < 0.01$, ***$P < 0.001$, ****$P < 0.0001$ (Exact *P* values are provided in Appendix Table S1). Scale bar = 10 μm. Source data are available online for this figure.

© The Author(s)

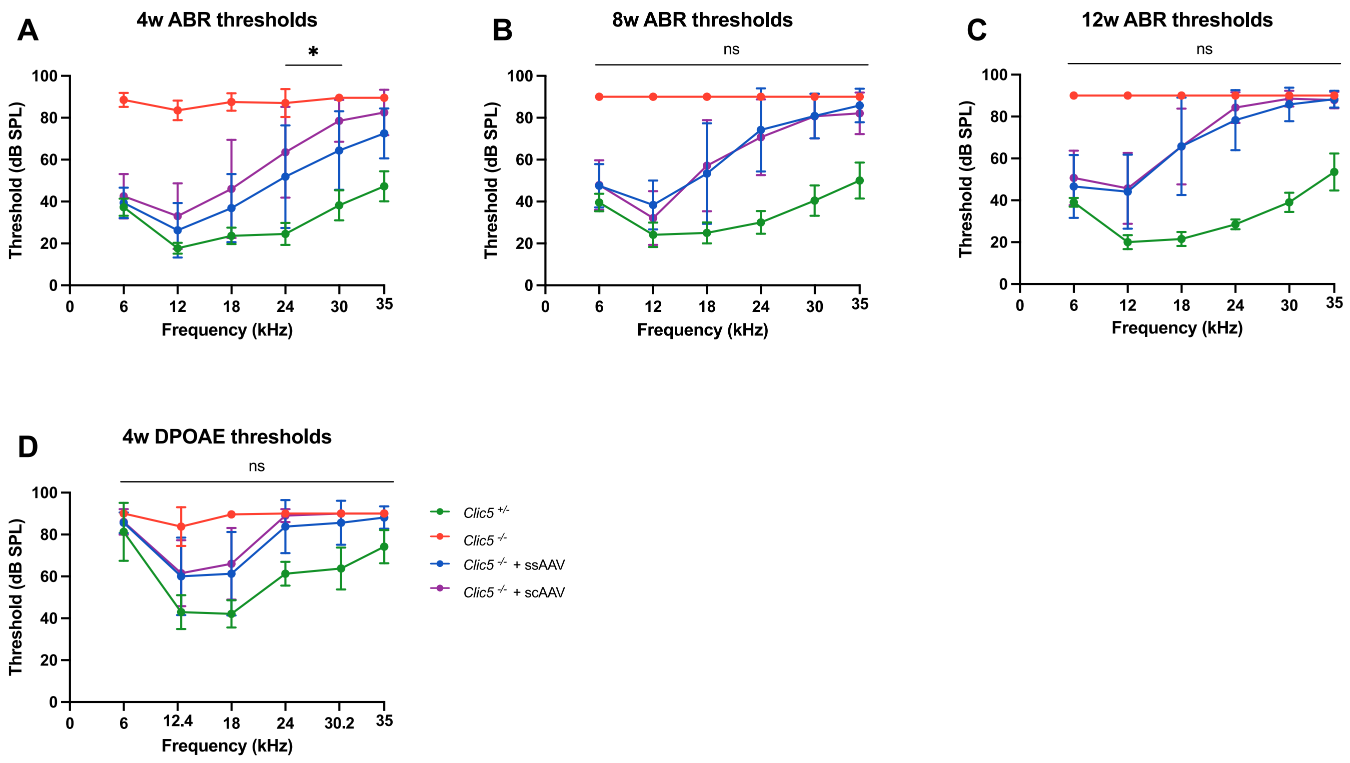

Figure EV4. Comparison of single-stranded and self-complementary vectors for auditory function restoration.

(**A**) ABR thresholds at 4 weeks of *Clic5*$^{+/-}$ ($n = 11$), *Clic5*$^{-/-}$ ($n = 10$), and *Clic5*$^{-/-}$ injected with ssAAV.*Clic5* ($n = 8$) or scAAV.*Clic5* ($n = 10$). (**B**) ABR thresholds at 8 weeks of *Clic5*$^{+/-}$ ($n = 11$), *Clic5*$^{-/-}$ ($n = 11$), and *Clic5*$^{-/-}$ injected with ssAVV.*Clic5* ($n = 6$) or scAAV.*Clic5* ($n = 7$). (**C**) ABR thresholds at 12 weeks of *Clic5*$^{+/-}$ ($n = 10$), *Clic5*$^{-/-}$ ($n = 10$), and *Clic5*$^{-/-}$ injected with ssAAV.*Clic5* ($n = 6$) or scAAV.*Clic5* ($n = 7$). (**D**) DPOAE thresholds at 4 weeks of *Clic5*$^{+/-}$ ($n = 12$), *Clic5*$^{-/-}$ ($n = 12$), and *Clic5*$^{-/-}$ injected with ssAVV.*Clic5* ($n = 8$) or scAAV.*Clic5* ($n = 10$). Data information: Statistical tests were two-way ANOVA with Holm–Sidak correction for multiple comparisons. Plots show mean ± SD. ns = not significant, $^{*}P < 0.05$ (Exact P values are provided in Appendix Table S1). Source data are available online for this figure.

 © The Author(s)

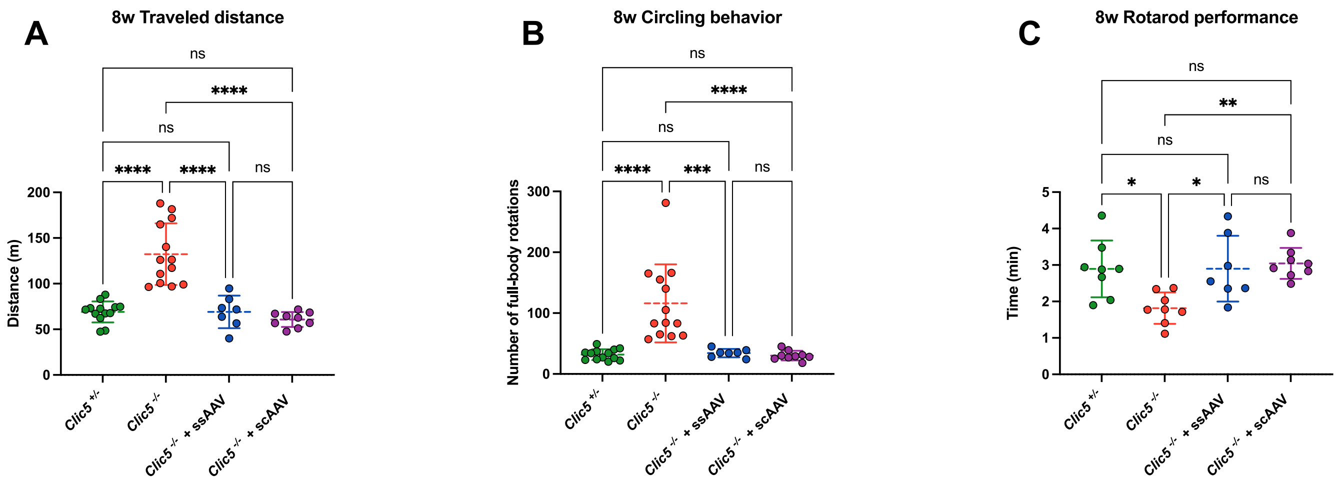

Figure EV5. Comparison of single-stranded and self-complementary vectors for vestibular function restoration.

(**A**) Distance traveled in the open-field test of *Clic5*$^{+/-}$ ($n = 13$), *Clic5*$^{-/-}$ ($n = 13$), and *Clic5*$^{-/-}$ injected with ssAAV.*Clic5* ($n = 7$) or scAAV.*Clic5* ($n = 9$), performed at 8 weeks. (**B**) Quantification of circling behavior during the open field test *Clic5*$^{+/-}$ ($n = 13$), *Clic5*$^{-/-}$ ($n = 13$), and *Clic5*$^{-/-}$ injected with ssAAV.*Clic5* ($n = 7$) or scAAV.*Clic5* ($n = 9$), performed at 8 weeks. (**C**) Quantification of the average duration on the rotarod of *Clic5*$^{+/-}$ ($n = 8$), *Clic5*$^{-/-}$ ($n = 8$), and *Clic5*$^{-/-}$ injected with ssAAV.*Clic5* ($n = 7$) or scAAV.*Clic5* ($n = 8$), performed at 8 weeks. Data information: The statistical test was One-way ANOVA followed by Tukey correction for multiple comparisons. Plots show mean ± SD. ns = not significant, *$P < 0.05$, **$P < 0.01$, ***$P < 0.001$, ****$P < 0.0001$ (Exact *P* values are provided in Appendix Table S1). Source data are available online for this figure.

© The Author(s)
